# Rare-Earth Metal Complexes of the Antibacterial Drug Oxolinic Acid: Synthesis, Characterization, DNA/Protein Binding and Cytotoxicity Studies

**DOI:** 10.3390/molecules25225418

**Published:** 2020-11-19

**Authors:** Ana-Madalina Maciuca, Alexandra-Cristina Munteanu, Mirela Mihaila, Mihaela Badea, Rodica Olar, George Mihai Nitulescu, Cristian V. A. Munteanu, Marinela Bostan, Valentina Uivarosi

**Affiliations:** 1 Department of General and Inorganic Chemistry, Faculty of Pharmacy, Carol Davila University of Medicine and Pharmacy, 6 Traian Vuia St, 020956 Bucharest, Romania; ana-madalina.maciuca@drd.umfcd.ro; 2 Center of Immunology, Stefan S. Nicolau Institute of Virology, 285 Mihai Bravu Ave, 030304 Bucharest, Romania; mirela.mihaila@virology.ro (M.M.); marinela.bostan@yahoo.com (M.B.); 3 Department of Inorganic Chemistry, Faculty of Chemistry, University of Bucharest, 90-92 Panduri Str, 050663 Bucharest, Romania; mihaela.badea@chimie.unibuc.ro (M.B.); rodica.olar@chimie.unibuc.ro (R.O.); 4 Department of Pharmaceutical Chemistry, Faculty of Pharmacy, Carol Davila University of Medicine and Pharmacy, 6 Traian Vuia Str, 020956 Bucharest, Romania; george.nitulescu@umfcd.ro; 5 Department of Bioinformatics and Structural Biochemistry, Institute of Biochemistry of the Romanian Academy (IBRA), 296 Spl. Independenţei, 060031 Bucharest, Romania; cristian.munteanu@biochim.ro

**Keywords:** drug repositioning, quinolone, oxolinic acid, rare-earth metal ions, anticancer, DNA binding, serum proteins

## Abstract

“Drug repositioning” is a current trend which proved useful in the search for new applications for existing, failed, no longer in use or abandoned drugs, particularly when addressing issues such as bacterial or cancer cells resistance to current therapeutic approaches. In this context, six new complexes of the first-generation quinolone oxolinic acid with rare-earth metal cations (Y^3+^, La^3+^, Sm^3+^, Eu^3+^, Gd^3+^, Tb^3+^) have been synthesized and characterized. The experimental data suggest that the quinolone acts as a bidentate ligand, binding to the metal ion via the keto and carboxylate oxygen atoms; these findings are supported by DFT (density functional theory) calculations for the Sm^3+^ complex. The cytotoxic activity of the complexes, as well as the ligand, has been studied on MDA-MB 231 (human breast adenocarcinoma), LoVo (human colon adenocarcinoma) and HUVEC (normal human umbilical vein endothelial cells) cell lines. UV-Vis spectroscopy and competitive binding studies show that the complexes display binding affinities (K_b_) towards double stranded DNA in the range of 9.33 × 10^4^ − 10.72 × 10^5^. Major and minor groove-binding most likely play a significant role in the interactions of the complexes with DNA. Moreover, the complexes bind human serum albumin more avidly than apo-transferrin.

## 1. Introduction

In the context of the emergent resistance of bacteria strains and cancer cell lines to currently approved drugs, one strategy which has proved successful is that of “drug repositioning”. This refers to a process based on finding new applications for existing, failed, no longer in use or abandoned drugs [1]. The growing interest in synthesizing new metal complexes of different antibiotics is due to their new found pharmacological and toxicological properties [2,3].

Quinolones represent a group of synthetic antibacterial agents characterized by a broad spectrum of activity and good bioavailability [4,5] used in the treatment of infections with various localizations [6]. The mechanism is a complex one and implies binding to topoisomerase II (DNA-gyrase) [7,8] and topoisomerase IV [9,10]. This process induces bacteriostasis [11], double-strand breaks, chromosome fragmentation, formation of reactive oxygen species (ROS) [11,12] and eventually cell death. Mg^2+^ ions [13] play a key role in the formation of the quinolone-bacterial DNA-enzyme complex: they coordinate two oxygen atoms from the quinolone molecule and four water molecules in an octahedral manner [14], thus stabilizing the whole structure. Quinolones have a series of properties that make them suitable for repositioning as anti-cancer drugs, such as: immunomodulation [15], pro-apoptotic and antiproliferative properties [16,17,18,19,20,21] and anti-metastatic potential [22,23].

**Figure 1:**
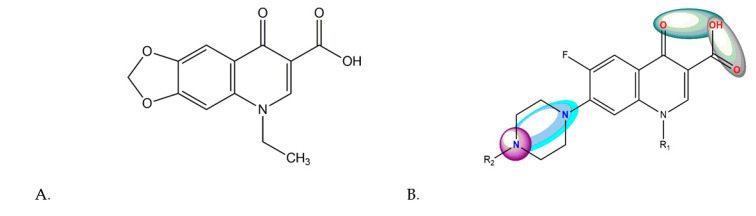
(**A**) Oxolinic acid; (**B**) Metal coordination sites in the general structure of quinolones.

Oxolinic acid (5-ethyl-5,8-dihydro-8-oxo-l,3 dioxolo[4,5-*g*]quinoline-7-carboxylic acid, Figure 1A) is a first generation quinolone, developed by Warner-Lambert, approved by the FDA in the 1970s for the treatment of bacterial urinary infections not associated with bacteremia in adults [24]. The spectrum of activity is rather narrow and includes some Gram-negative bacteria [25,26,27]. Still, it has not proven itself to be more effective or better tolerated than other drugs such as nalidixic acid, ampicillin or trimethoprim-sulfamethoxazole [24,28,29]. Evidence shows that the efficacy of the treatment is not dose-related [24] and there have been some observations about the drug’s natural tendency to give rise to resistant mutants at a daily dose of less than 2 g/day [30]; these are aspects that have limited the use of oxolinic acid in humans. Nevertheless, it has been approved for use in the treatment of enteric infections in various animal species such as poultry, pigs, calves and fish [31] and for the management of pine wilt disease [32].

Several features in their structures (see Figure 1B) render quinolones good candidates for metal binding: the carboxyl moiety in position 3, the carbonyl moiety in position 4 and one or both nitrogen atoms from the piperazinyl ring in position 7, in the case of fluoroquinolones; the working conditions and the nature of the metal [33,34,35] determine which of the moieties will be involved in complex formation. So far, a considerable number of quinolone-metal complexes have been synthesized and characterized from the chemical and biological point of view; evidence that these complexes bind to calf thymus DNA [36,37,38,39] and that numerous complexes exhibit antibacterial [40,41,42,43], antitumoral [40,44,45], antifungal [40,45] and antiparasitic [46] properties have led to a growing interest in these complexes. 

An intention of repurposing quinolones as anti-cancer agents has been observed and it stands on the evidence that some quinolone-metal complexes present antitumoral activity against leukemia [47] and breast cancer cells [44,48]; gold and ruthenium complexes have been found to be effective against metastatic cancer cells [49], without being toxic to normal cells [50], and present varied mechanisms of action [1].

Lanthanide (Ln^3+^) complexes with different drugs are appealing due to several properties [51]: the ability to bind to serum albumin [52], the development of stable MRI contrast agents [53,54,55] and the ability to replace cations such as Ca^2+^, Mg^2+^ or Zn^2+^ as co-factors in metaloenzymes with hydrolytic activity [56,57,58,59,60]. The ability to bind to DNA [61,62] and their luminescent properties [63,64,65] render Ln^3+^ and their complexes as potential candidates for nucleic acid imaging [66]. Lanthanides present a high affinity for ligands with donor atoms in the order O > N > S and coordination numbers that vary from 6 to 12 [67].

Up to date, several metal complexes of oxolinic acid have been reported [68,69,70,71,72,73,74,75,76,77,78,79,80]. Oxolinic acid can be considered a good candidate for the formation of complexes with Ln^3+^ metal ions, due to its adjacent oxygen-bearing moieties and the oxophilicity of the Ln^3+^ ion. We present herein the synthesis and physicochemical characterization of six novel complexes with some rare-earth metal cations and the results of the biological studies.

## 2. Results and Discussion

### 2.1. Syntheses of the Complexes

**Scheme 1:**
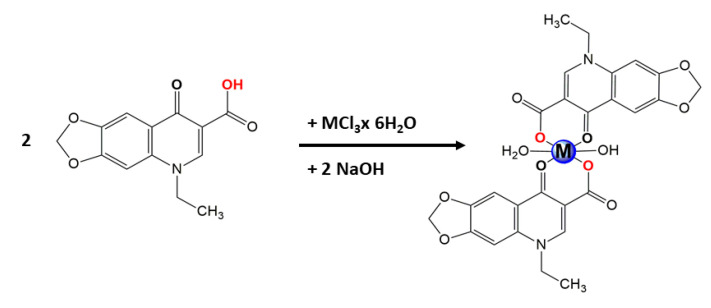
Synthesis scheme for complexes of oxolinic acid; M = Y^3+^, La^3+^, Sm^3+^, Eu^3+^, Gd^3+^, Tb^3+^.

We report the synthesis of six new complexes of M^3+^ (M = Y^3+^, La^3+^, Sm^3+^, Eu^3+^, Gd^3+^, Tb^3+^) with oxolinic acid, in a molar ratio of 1:2. Briefly, the sodium salt of the ligand, together with the metal salt reacted at 150 °C in a microwave oven under continuous stirring (Scheme 1). Based on the experimental and some literature data [81,82], the following formulas were attributed to the complexes: **[Y(oxo)(OH)(H_2_O)] (Y oxo), [La(oxo)(OH)(H_2_O)] (La oxo), [Sm(oxo)(OH)(H_2_O)] · H_2_O (Sm oxo), [Eu(oxo)(OH)(H_2_O)] (Eu oxo), [Gd(oxo)(OH)(H_2_O)]·0.5H_2_O (Gd oxo), [Tb(oxo)(OH)(H_2_O)] · 0.5 H_2_O (Tb oxo)**, where oxo is the deprotonated ligand.

The complexes were obtained as white powders and the solubility of the complexes was tested in various solvents of different polarities; the complexes are soluble in DMSO, slightly soluble in DMF and insoluble in methanol, ethanol, acetone, chloroform, diethyl ether, tetrahydrofuran and water. 

Structural characterization included elemental analysis, spectroscopy studies (UV-VIS-NIR, FT-IR and high-resolution mass spectrometry) and thermal analysis.

### 2.2. Physicochemical Characterization of the Complexes

#### 2.2.1. UV-Vis-NIR Spectra

**Figure 2:**
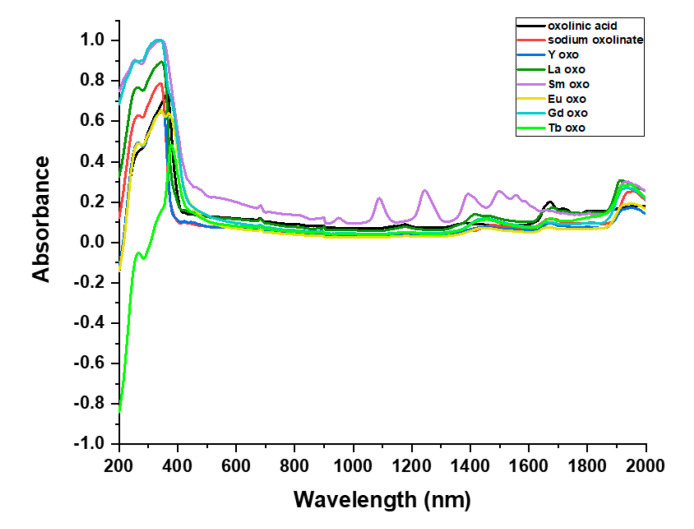
UV-Visible spectra of oxolinic acid, sodium oxolinate and **M oxo** (M = Y^3+^, La^3+^, Sm^3+^, Eu^3+^, Gd^3+^, Tb^3+^).

UV-Vis-NIR spectra bring evidence of metal complex formation (Figure 2). The spectrum of the free ligand presents the characteristic absorption peaks in the 240–300 nm region, due to the absorption of the aromatic ring [83], and the 300–380 nm interval due to the n→π* (HOMO-LUMO) transitions [83]. Upon complexation, it can be observed that the peaks undergo a hypsochromic shift (~20 nm for the Y^3+^, La^3+^, Sm^3+^, Eu^3+^ complexes and 25 nm for the Gd^3+^ complex) due to metal quinolone interaction related to intra-ligand transitions [83]. **Sm oxo** presents specific absorption bands in the NIR region due to f-f transitions from the ground state ^6^H_5/2_ to ^6^F multiplet of Sm^3+^ ion (4f^5^) [84,85] (Figure 2). The wavelengths and corresponding absorbances are summarized in Table S1. The band at ~425 nm in the spectrum of **Y oxo** can be assigned to the ligand-metal charge transfer [86].

#### 2.2.2. FT-IR Spectra

Coordination of the rare-earth metal ions by oxolinic acid was evaluated by means of Fourier transform infrared spectroscopy (Figures S1–S8) by comparing the spectra of the complexes with that of the free ligand; the obtained values are given in detail in Table 1. In order to determine the manner of coordination, specific peaks have been observed; changes in intensity and/or position are due to their implication in the chelation process [68,71,72,73]. The broad bands in the 3800–3300 cm^−1^ confirm the presence of water (lattice or coordinated) and a possible hydroxyl group coordinated to the metal. Two specific bands can be observed in the spectrum of the free ligand: at 1698 cm^−1^ due to the stretching vibration of the νC=O bond of the carboxylic acid moiety (νC=Oc) and at 1632 cm^−1^ due to the stretching vibration of the pyridone bond (νC=Op) [87]. The disappearance of the first band and the shift of the second one to lower values varying from 1594 cm^−1^ (**Y oxo**) to 1562 cm^−1^ (**Tb oxo**) are indicative of a bidentate manner of coordination of the ligand, through one oxygen atom from the carboxyl moiety and one from the carbonyl group. Due to the deprotonation process, two new bands appear in the spectra of the complexes, which can be attributed to the νO-C-O asymmetric (νO-C-Oas ~ 1630 cm^−1^) and νO-C-O symmetric (νO-C-Os-~1340 cm^−1^) stretching vibrations. In order to evaluate the binding mode of the carboxylate moiety to the metal, the Δ (νO-C-Oas - νO-C-Os) value was calculated and compared with the one corresponding to sodium oxalinate; these values vary from 292 (for **Y oxo**, **La oxo**, **Gd oxo**) to 295 (**Tb oxo**) (Table 1). The Δ values are higher than 200 and indicate that the carboxylate moiety coordinates the metal ion in a monodentate way. Overall, the oxolinic acid acts as a bidentate ligand through the carboxylic and the pyridonic oxygen atoms [88]. In the 500–400 cm^−1^ region of the spectra of the complexes (Table 1) new bands can be observed which can be attributed to the stretching vibration of the metal-oxygen bond [87], since they do not appear in the spectra of the free ligand or the sodium salt, confirming, once more, that the chelating process takes place. The complexes present similar wavenumbers for the studied vibrations, confirming that they have similar structures.

#### 2.2.3. Mass Spectra

A typical isotopic distribution for ions of the type [M(oxo)_2_(DMSO)_2_]^+^ can be observed in the mass spectra (Figures S9–S13); due to the sample treatment (the solid powders were dissolved in DMSO and then diluted to a working concentration in methanol) used prior to the injection, the coordinated water molecule and hydroxyl group have been replaced by two DMSO molecules. Generally, we also observed peaks corresponding to a gradual loss of the DMSO molecules in the MS/MS spectra of some complexes such as **Sm oxo**, **Eu oxo** or **Gd oxo** (Figures S10–S12). Peak assignments are given in the Experimental section.

#### 2.2.4. Thermal Behavior

**Figure 3:**
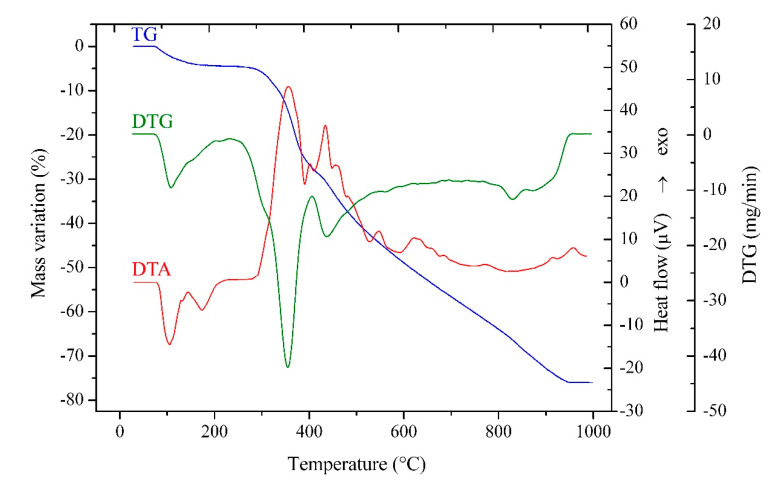
TG, DTG and DTA curves for **Sm oxo**.

The study of thermal behavior of complexes is useful for elucidating certain aspects about their composition, namely the number and nature of water molecules and the stoichiometry [89,90]. The analysis of the thermal decomposition curves brings additional confirmation concerning the structural composition of the complexes. The decomposition process is complex for all species and consists of water elimination as well as the oxidative degradation of the coordinated oxolinate anion (Table 2). The thermogravimetric (TG) curves confirm the number of water molecules from the structure of the metal complexes, both crystallization and coordination water molecules being eliminated together. In accordance with both derivative thermogravimetry (DTG) and differential thermal analysis (DTA) curves, the oxidative degradation of the organic part occurs in several overlapped exothermic processes, as can be seen in Figure 3 for **Sm oxo**. The overall mass loss is consistent with M_2_O_3_ stabilization as final residue, with the exception of **Tb oxo**, for which Tb_4_O_7_ is formed.

### 2.3. DFT Calculations

**Figure 4:**
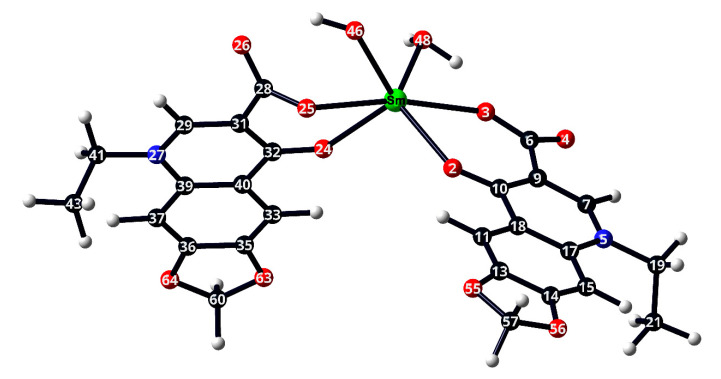
Optimized molecular geometry of **Sm oxo** at the B3LYP/ ECP52MWB (Sm)/ 6-31G(d,p) (H, C, O atoms) level in Gaussian 09 software package; the Sm atom isshown in gree, the O atoms in red, the N atoms in blue, C atoms in black and H atoms in white.

Since all attempts to isolate single crystals of the complexes have failed, the geometry optimization for **Sm oxo** was carried out via density functional theory (DFT) studies. Basis set 6-31G(d,p) was used for all H, C and O atoms and the small core potential ECP52MWB was used for Sm, as previously reported [91]. The fully optimized geometry of the complex is shown in Figure 4. The selected bond lengths, angles and charge densities are presented in Table 3. The coordination sphere around the metal center in the complexes involve the following atoms: O_3_, O_25_ (belonging to the deprotonated carboxylic group), O_2_, O_24_ (belonging to the carbonyl groups) and O_46_, O_48_ (belonging to a hydroxyl group and a water molecule, respectively). The M-O bond lengths are comparable with those previously reported for other complexes with quinolones [91,92,93,94,95]: the bond distances between Sm-O_2_ and Sm-O_3_, Sm-O_24_ and Sm-O_25_ are similar to values of 2.517 and 2.506 Å, 2.582 and 2.519 Å, respectively; still, it can be observed how the bond between Sm^3+^ and the oxygen atoms from the carboxyl moieties are shorter than the ones Sm^3+^ forms with the pyridonic oxygens. The bonds length for Sm-O_46_ is 2.557 Å, while the one for Sm-O_48_ is 2.664 Å. The O-Sm-O bond angles vary from 53.623° (O_46_-Sm-O_25_) to 134.706° (O_3_-Sm-O_48_) and indicate a distorted octahedral geometry. The dihedral angles present values different from 0° or 180°, varying from the only positive value, 87.515 (O_46_-Sm-O_25_-C_28_), to the largest negative one, −130.417 (O_46_-Sm-O_2_-C_10_), indicating that the Sm^3+^ cation and the donating atoms do not lie in the same plane; an exception is made by the O_48_-Sm-O_3_-C_6_ angle which presents a value of −176.277°, close to −180° meaning that the atoms involved are in the same plane. The charges accumulated on the oxygen atoms of de ligand structure vary from −0.530 (O_25_) to −0.594 (O_24_).

Furthermore, we have used the calculated vibrational frequencies to validate the optimized structures of the complexes and to aid the assignment of the experimental IR results. The calculated frequencies agree with the experimental data. The predicted IR spectra for the complex are shown in Figure S5 and the selected data, as well as the assignments, are given in Table 4. In the 3400–3200 cm^−1^ spectral region of the experimental IR spectrum of **Sm oxo**, the O-H stretches give weak bands, which can be found in the predicted spectrum at 3617 (weak) and 3309 (strong) cm^−1^. Moreover, strong bands at 1634 cm^−1^ (experimental spectrum) and 1758 and 1695 cm^−1^ (predicted spectrum), respectively, are due to the νO-C-Oas and νO-C-Os stretching vibrations and confirm the involvement of the carboxylic moiety in coordination. The strong bands at 1576 cm^−1^ (experimental) and 1608 cm^−1^ (predicted spectrum), respectively, also confirm the involvement of the pyridonic carbonyl in metal binding.

### 2.4. Biological Studies

#### 2.4.1. Cytotoxicity Studies

For the evaluation of the cytotoxic effects of the complexes and ligand two human cancer cell lines were used, namely MDA-MB 231 (human breast adenocarcinoma) and LoVo (human colon adenocarcinoma); a normal cell line, human umbilical vein endothelial cells (HUVEC), was used as a reference. The effects were compared to the ones produced by adriamycin (ADR) and Cisplatin (Cis-Pt), respectively, which were used as positive controls. After 24 h and 48 h, respectively, cell viability was evaluated through MTS assay (data shown in Figures S16–S18) and the IC_50_ values were calculated (Table 5).

In terms of IC_50_ values, **Sm oxo** and **Tb oxo** displayed similar activity to the positive control, cisplatin, on LoVo cells. All complexes, however, displayed higher cytotoxic activity as compared to the free ligand. Interestingly, when tested on MDA-MB 231 cells, oxolinic acid showed higher activity in comparison with **La oxo**, **Eu oxo**, **Gd oxo**, **Tb oxo**. Although all compounds displayed lower activity than the positive control, ADR, the lowest IC_50_ values on MDA-MB 231 cells have been obtained for **Y oxo** (33.22 ± 14.92 µM) and **Sm oxo** (40.42 ± 6.27 µM). Altogether, the most promising results have been obtained for **Sm oxo**, which was moderately active on both tested cell lines. Regarding the effects shown on the HUVEC cell line, the IC_50_ values of the complexes are indicative of a lower toxicity compared to Cis-Pt.

#### 2.4.2. Studies on DNA Binding

The stability of the complexes in solution has been investigated by means of UV-visible spectroscopy. The samples were prepared in Tris-HCl buffer as all biological interactions (with DNA and proteins) are tested in this medium. The recorded spectra are shown in Figures S14 and S15. No noticeable changes in absorbance or number and position of the peaks occur (data presented in Table S2). Since significant variation of the absorbances was not observed, the complexes can be considered stable in the given tested solution and time frame.

##### UV-Vis Spectra

Metal complexes interact with the DNA macromolecule through three major processes: (i) through electrostatic interactions with the negatively charged sugar-phosphate structure, (ii) via minor or major groove binding, through van der Waals forces, hydrogen bonds, electrostatic forces, hydrophobic interactions and (iii) by intercalation between the base-pairs, processes stabilized by the π-π* stacking interactions between the aromatic systems of the ligand and the DNA bases. Effective binding results in modifications of the UV-Vis spectra of the DNA sample, such as: hypochromic, hypsochromic (blue-shift) or bathochromic (red-shift) changes [84,96,97]. Other oxolinic acid metal complexes have been reported to possess DNA-binding properties [71,72,73], hence it has been hypothesized that DNA could act as a target for these new complexes as well.

**Figure 5:**
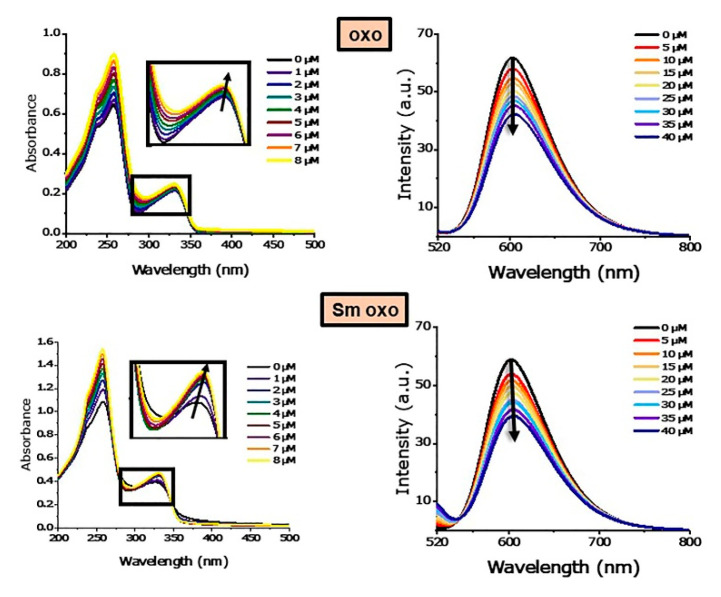
**Left:** Absorption spectra of **oxo** and **Sm oxo** in the absence and presence of increasing amounts of DNA. [**compound**] = 20 μM; [**DNA**] = 0, 5, 10, 15, 20, 25, 30, 35, 40 μM. The arrow shows the absorption changes upon the increase of the DNA concentration. **Right:** Fluorescence spectra of the EB-DNA system in the absence and presence of increasing amounts of the tested compounds. λ_ex_ = 500 nm, [**EB**] = 2 μM, [**DNA**] = 10 μM, [**compound**] = 0, 5, 10, 15, 20, 25, 30, 35, 40 μM. Arrows indicate the changes in fluorescence intensities upon the increase of the concentrations of the tested compounds.

Changes in the UV spectra of the seven studied compounds (Figure 5 and Figure S19) upon the addition of increasing concentrations of CT-DNA have been analyzed. The maximum shift observed for the band centered at ~330.5 nm is of 2 nm (Figure S19—Gd oxo). The values of the binding constant (K**_b_** shown in Table 6 and Figure S20) are useful in evaluating the magnitude of the binding strength; a maximum value is observed for **Eu oxo** (10.72 (± 2.47) × 10^5^ L∙mol^−1^) with a decrease in the K_b_ values in the following order: **Eu oxo** > **Tb oxo** > **Gd oxo** > **Y oxo** > **La oxo** (2.57 (± 0.17) × 10^5^ L∙mol^−1^ ), dropping to **Sm oxo** which presents a value of 9.33 (± 1.46) × 10^4^ L∙mol^−1^ (Table 6). The free ligand presents a K**_b_** value in the range of 10^4^, suggesting that complexation slightly improves the affinity of oxolinic acid for DNA. Similar changes are observed in the UV-vis spectra, suggesting similar binding modes for all complexes. The K**_b_** values suggest a relatively moderate binding of the complexes to CT-DNA [71]. These results suggest that all complexes interact with the DNA macromolecule, although they do not give information about the type of binding.

##### Competitive Binding Assay with Ethidium Bromide (EB) through Fluorescence Spectroscopy

The manner of binding to the DNA macromolecule can be investigated through competitive binding studies with ethidium bromide (3,8-diamino-5-ethyl-6-phenylphenanthridinium bromide), a fluorescent dye; due to its aromatic ring, it can intercalate between two adjacent base pairs in the DNA structure and form a complex which emits fluorescent light; displacing EB results in a quenching effect [84,98]. The Stern-Volmer constant (K**_SV_**) and the dissociation constant (K**_50_**) have been calculated from the resulting data.

The emission spectra of the EB-DNA system recorded in the absence and presence of increasing concentrations of the studied compounds are presented in Figure S21. Quenching of fluorescence can be observed for all the compounds. While the spectra suggest that displacement of EB from EB-DNA-system takes place (Table 6) [72], the K**_SV_** values (Figure S22) suggest a moderate interaction, most likely through groove binding; still, an intercalative binding mode cannot be completely ruled out. **La oxo** presents the highest K**_SV_** value (1.27 (±0.03) × 10^4^), affinities dropping in the order of **Eu oxo** > **Sm oxo** > **Gd oxo** = **Tb oxo** > oxolinic acid > **Y oxo** (8.38 (±0.29) × 10^3^). Interestingly, K**_50_** values (Figure S23) suggest that the strongest interaction (resulting in the most stable complex) occurs between **Tb oxo** and DNA, with a value of 20.28 ± 1.15 µM; values rise in the following order: **Y oxo** < **La oxo** < **Eu oxo** < **Sm oxo** < oxolinic acid < **Gd oxo** (47.75 ± 25.22 µM).

#### 2.4.3. Interactions with Human Serum Albumin (HSA) and Apo-Transferrin (apo-Tf)

More evidence emerges indicating that the main transport proteins for metal complexes in the human body are albumin and transferrin. Human serum albumin (HSA) is the most abundant protein in the human circulatory system, being responsible for 80% of the colloid osmotic pressure [99]; amongst proteins, it possesses the highest affinity for drugs, influencing their pharmacokinetic and pharmacodynamic profiles. Apo-transferrin (apo-Tf) is mainly involved in the transport of iron (mainly Fe^3+^) and various xenobiotics [100,101]. It exhibits a significant tropism for tumor cells on account of the numerous specific receptors expressed on their surface [100,102,103] and because of this it has been investigated as a potential drug carrier for targeted delivery into tumor cells [104].

Insight into the nature of the interactions between proteins and complexes can be obtained by analyzing their fluorescence spectra; the tryptophane residue in position 214 (Trp-214) is highly sensitive to changes in the polarity of the medium [84,105]; due to the tryptophane residue, a strong peak is observed at ~340 nm when HSA samples are excited at 280 nm. Similarly, when excited at 295 nm, apo-Tf samples present a maximum emission peak at ~327 nm. These peaks can undergo changes upon interaction with different compounds.

##### Studies Regarding the Fluorescence Quenching Mechanism

**Figure 6:**
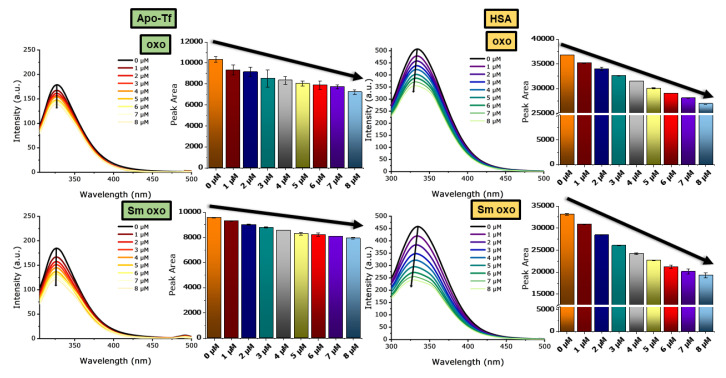
The fluorescence intensities of the free proteins (apo-Tf and HSA) decrease upon the addition of increasing amounts of **Sm oxo**. [**apo-Tf**] = 1 μM, [**HSA**] = 2.5 μM, [**Sm oxo**] = 0, 1, 2, 3, 4, 5, 6, 7, 8 μM. Black arrows indicate that the peak areas decrease with increasing concentrations of the complex. The effects are more prevalent for **Sm oxo** than for the free ligand, as well as for HSA as compared to apo-Tf, indicating a stronger affinity of the compounds for the former protein.

The HSA peak recorded at 335 nm suffered slight hypsochromic shifts (Figure 6 and Figure S24), indicating a diminished exposure of the Trp-214 residue to the solvent. A moderate quenching effect can be observed varying from ~47% (**Gd oxo**) to ~58% (**Eu oxo**). The apo-Tf peak recorded at 327 nm, on the other hand, suffers no shift (Figure 6 and Figure S25) and the observed quenching effect is smaller than that in the case of HSA, varying from 35 % (**Y oxo**) to 50% (**Eu oxo**). Judging by this observation, **Eu oxo** seems to present the highest affinity for both proteins; it is followed, in both cases, by **Tb oxo** (52.63% and 47.62% for HAS and apo-Tf, respectively). Peak areas decrease with increasing concentrations of the tested compounds (Figures S26 and S27). The effects are more prevalent for the complexes than for the free ligand, as well as for HSA as compared to apo-Tf, indicating a stronger affinity of the compounds for the former protein.

More in-depth information was obtained by building and analyzing Stern–Volmer plots (F_0_/F vs. [Q]) and modified Stern-Volmer plots (F_0_/(F_0_-F) vs. 1/[Q]) for both HSA (Figure S28) and apo-Tf (Figure S29) interactions; the K_SV_ (Stern–Volmer quenching constant), K_q_ (bimolecular quenching constant) and K_a_ (association binding constant) were calculated and interpreted.

A number of processes can explain the observed quenching effect, such as molecular collision, energy transfer, excited-state reaction or ground-state complex formation. However, three main processes are relevant for small molecules: static quenching (which involves the formation of a nonfluorescent complex between the quencher and the fluorophore in the ground state), dynamic quenching (the excited state of the fluorophore loses energy via a collisional process with the quencher) and combined static and dynamic quenching. 

The calculated values for K_SV_ and K_q_ (Table 7) give information about the static or dynamic nature of the quenching phenomenon. In the case of apo-Tf, the K_SV_ constant reaches its maximum value for **Tb oxo** (19.45 (± 1.10) × 10^4^ M^−1^) and decreases in the order **Gd oxo** > oxolinic acid > **Eu oxo** > **La oxo** > **Sm oxo** > **Y oxo** (6.52 (± 0.56) × 10^4^ M^−1^), while for HSA the order in which the values decrease is **Eu oxo** (11.25 (± 0.92) × 10^4^ M^−1^) > **Y oxo** > **La oxo** > **Sm oxo** > **Gd oxo** > oxolinic acid (1.95 (± 0.38) × 10^4^ M^−1^). The K_q_ values follow the same pattern and are higher than diverse kinds of quenchers for biopolymers fluorescence (2.0 × 10^10^ M^−1^s^−1^), suggesting a static quenching mechanism [106].

The association binding constant, K_a_ (M^−1^), and the number of binding sites, n, were also calculated using Equation (7) (Table 7); K_a_ values indicate good HSA and apo-Tf binding, but a higher affinity towards HSA. **Tb oxo** (6.83(±1.33) × 10^5^ M^−1^) and **Y oxo** (4.40(±1.09) × 10^4^ M^−1^) present the highest affinities towards HSA and apo-Tf, respectively; in the case of both proteins, the lowest K_a_ values (Figures S30 and S31) correspond to the free ligand and are 10 times smaller than the ones for the complexes, indicating a rise in affinity that comes with coordination. The number of binding sites (n) has been found to be ~1 for both HSA and apo-Tf and all of the tested compounds.

Interestingly, plots of F_0_/F vs. [Q] (the Stern–Volmer plots) present a slightly downward curvature, concave towards the x-axis in the case of **Sm oxo** interacting with HSA (Figure S28) and oxolinic acid, **Gd oxo** and **Tb oxo** when interacting with apo-Tf (Figure S29). This could be explained by a selective quenching phenomenon of accessible versus inaccessible or partially accessible tryptophane residue [107].

K_d_ constants (Figures S30 and S31) have been calculated from Equation (8) and have been found to be less than 9 µM for both HSA and apo-Tf. Most of the calculated Hill coefficient (n) values are close to the value of 2, indicating positively cooperative binding. However, there are several exceptions (e.g., **Eu oxo** interacting with both proteins) where the coefficient is closer to the value of 1, indicating a negatively cooperative binding [108]. Overall, the values obtained for the binding affinities (in the micromolar range) are optimal and indicate that the complexes can be carried by the serum proteins throughout the human body. Moreover, the low micromolar values obtained for K_d_ constants are not high enough to cause significant drug retention and, therefore, to decrease the concentration and distribution of the free drug species to the cellular targets, and, hence, their efficacy.

##### Studies on Conformational Changes of HSA and Apo-Tf Due to the Interaction with the Tested Compounds

Structural changes due to interaction with drug molecules can be studied by measuring the synchronous spectra; using the established wavelength intervals Δλ = 15 nm and Δλ = 60 nm, where Δλ = λ_em_ − λ_ex_, the tyrosine or tryptophane residues, respectively, can be highlighted. The tryptophane residue is sensitive to any change in hydrophobicity in its vicinity; any shift of the peak is due to a change in polarity of the medium surrounding the aminoacid: a blue shift is due to a reduction in exposure to the solvent, implying a more hydrophobic environment, whilst a red shift is determined by an increased exposure to the solvent and a more polar environment [105,109].

The synchronous spectra at Δλ = 15 nm and Δλ = 60 nm for HSA and apo-Tf, respectively, are shown in Figures S32–S35, respectively. There are no changes observed in the synchronous spectra recorded at Δλ = 15 nm for either of the two proteins. On the other hand, bathochromic shifts can be observed in the synchronous Δλ = 60 nm for both proteins; these changes imply that the proteins adopt a conformation which increases the exposure of the tryptophane residue to the solvent, creating a more polar environment in its vicinity.

## 3. Materials and Methods

### 3.1. Materials

All reagents and solvents were of analytical reagent grade and were used without further purification. Oxolinic acid, YCl_3_·6H_2_O, LaCl_3_, EuCl_3_·6H_2_O, GdCl_3_·6H_2_O, SmCl_3_·6H_2_O, TbCl_3_·6H_2_O, human transferrin, human serum albumin and double-stranded calf-thymus DNA were purchased from Sigma Aldrich Chemical Co. (Schnelldorf, Germany). The following abbreviations were used to designate signal intensity: b = broad, w = weak, m = medium, s = sharp.

### 3.2. Synthesis

The synthesis method requires two steps: (1) synthesis of the sodium salt of the oxolinic acid and (2) synthesis of the lanthanide complexes. For (1), oxolinic acid (0.9 mmol; 0.2351 g) were dissolved in NaOH solution (900 µL, 1 M), under continuous magnetic stirring and heating (50 °C); the solvent was removed, yielding a residual white solid of sodium oxolinate. Step (2) involved a microwave-assisted method: in a microwave vial, the sodium oxalinate, 0.3 mmols of hexahydrate (for the Y^3+^, 0.0910 g, Eu^3+^, 0.1099 g, Gd^3+^, 0.1115 g, Sm^3+^, 0.1094 g, and Tb^3+^, 0.1120 g, complexes) or anhydrous (for the La^3+^ complex, 0.0735 g) metal chloride and distilled water (2 mL) were added. The vile was kept in the microwave apparatus for 30 min, under stirring, at 165 °C. The yellow precipitates formed were filtered, washed with water (3 × 10 mL) and then dried in the dessicator.

**Y oxo**—Y(C_26_H_20_N_2_O_10_)(OH)(H_2_O), MW = 644.37 g/mol; Elemental analysis found (calculated): %C 48.97 (48.46), %H 3.53 (3.60), N% 3.89 (4.35); Molar conductance (DMSO, Ω^−1^∙cm^2^∙mol^−1^): 2.1; MS (ESI+): *m*/*z*: 761.04 ([Y(oxo)2(DMSO)2]^+^); UV-vis (nm): 1945, 345, 265; FT-IR (cm^−1^): 3724 (w, ν(O-H) coordinated water molecule), 3055 (w, (ν(O-H) lattice water), 2973 (w, ν(C-H)), 2924 (ν(C-H)), 1630 (s, ν(O-C-O)as), 1594 (s, ν(C=O)pyridone), 1338 (s, ν(O-C-O)s).
**La oxo**—La(C_26_H_20_N_2_O_10_)(OH)(H_2_O), MW = 694.37 g/mol; Elemental analysis found (calculated): %C 44.95 (44.97), %H 3.08 (3.34), N% 4.16 (4.03); Molar conductance (DMSO, Ω^−1^∙cm^2^∙mol^−1^): 6.6; MS (ESI+): *m*/*z*: 815.05 ([La(oxo)_2_(DMSO)_2_]^+^); UV-vis (nm): 1910, 345, 266; FT-IR (cm^−1^): 3392 (w, ν(O-H) coordinated water molecule), 3060 (w, ν(C-H)), 2982 (w, ν(C-H)), 1632 (s, ν(O-C-O)as), 1577 (s, ν(C=O)pyridone), 1340 (s, ν(O-C-O)s).
**Sm oxo**—[Sm(C_26_H_20_N_2_O_10_)(OH)(H_2_O) · H_2_O, MW = 723.84 g/mol; Elemental analysis found (calculated): %C 41.13 (43.14), %H 3.19 (3.48), N% 4.47 (3.87); Molar conductance (DMSO, Ω^−1^∙cm^2^∙mol^−1^): 2.7; MS (ESI+): m/z: 828.05 ([Sm(oxo)_2_(DMSO)_2_]^+^), 750.04 ([Sm(oxo)_2_(DMSO)]^+^), 672.02 ([Sm(oxo)_2_]^+^); UV-vis (nm): 1940, 1930, 1555, 1500, 1390, 1245, 1090, 345, 255; FT-IR (cm^−1^): 3345 (w, ν(O-H) coordinated water molecule), 2917 (w, ν(C-H)), 1634 (s, ν(O-C-O)as), 1576 (s, ν(C=O)pyridone), 1340 (s, ν(O-C-O)s).
**Eu oxo**—Eu(C_26_H_20_N_2_O_10_)(OH)(H_2_O), MW = 707.43 g/mol; Elemental analysis found (calculated): %C 43.91 (44.14), %H 2.92 (3.28), N% 4.24 (3.96); Molar conductance (DMSO, Ω^−1^∙cm^2^∙mol^−1^): 3.5; MS (ESI+): *m*/*z*: 829.05 ([Eu(oxo)_2_(DMSO)_2_]^+^), 751.04 ([Eu(oxo)_2_(DMSO)]^+^), 671.02 ([Eu(oxo)_2_]^+^); UV-vis (nm): 1945, 345, 270; FT-IR (cm^−1^): 3389 (w, ν(O-H) coordinated water molecule), 2984 (w, ν(C-H)), 1633 (s, ν(O-C-O)as), 1579 (s, ν(C=O)pyridone), 1340 (s, ν(O-C-O)s).
**Gd oxo**—[Gd(C_26_H_20_N_2_O_10_)(OH)(H_2_O)] · 0.5H_2_O, MW = 721.72 g/mol; Elemental analysis found (calculated): %C 43.52 (43.26), %H 3.17 (3.35), N% 4.17 (3.88); Molar conductance (DMSO, Ω^−1^∙cm^2^∙mol^−1^): 2.1; MS (ESI+): *m*/*z*: 834.05 ([Gd(oxo)_2_(DMSO)_2_]^+^), 756.04 ([Gd(oxo)_2_(DMSO)]^+^), 676.03 ([Gd(oxo)_2_]^+^); UV-vis (nm): 1950, 1940, 340, 260; FT-IR (cm^−1^): 3378 (w, ν(O-H) coordinated water molecule), 3059 (w, (ν(O-H) lattice water), 1633 (s, ν(O-C-O)as), 1579 (s, ν(C=O)pyridone), 1341 (s, ν(O-C-O)s).
**Tb oxo**—[Tb(C_26_H_20_N_2_O_10_)(OH)(H_2_O)] ·0.5H_2_O, MW = 723.40 g/mol; Elemental analysis found (calculated): %C 43.31 (43.16), %H 3.39 (3.35), N% 4.18 (3.87); Molar conductance (DMSO, Ω^−1^∙cm^2^∙mol^−1^): 3.2; MS (ESI+): *m*/*z*: 835.07 ([Tb(oxo)_2_(DMSO)_2_]^+^); UV-vis: 1945, 380; FT-IR (cm^−1^): 3391 (w, ν(O-H) coordinated water molecule), 3057 (w, (ν(O-H) lattice water), 2976 (w, ν(C-H)), 2921 (w, ν(C-H)), 1631 (s, ν(O-C-O)as), 1562 (s, ν(C=O)pyridone), 1336 (s, ν(O-C-O)s).

### 3.3. Physico-Chemical Characterization

Elemental analysis of C, H and N was performed on a PE 2400 analyzer (Perkin Elmer) (Billerica, MA, USA). Infrared spectra were recorded on a FT-IR VERTEX 70 spectrometer (Bruker: Billerica, MA, USA), using KBr pellets. UV-visible-NIR spectra were recorded on solid probes, by diffuse reflectance method, using spectralon as a reference sample and without dilution, in the range of 200–2000 nm, on a V-670 spectrophotometer (Jasco: Tokyo, Japan). The conductivity was measured with a Consort C830 (Turnhout, Belgium) conductometer with an SK10T platinum electrode embedded in glass (cell constant 1.0 cm^−1^) for 10^−3^ M solutions in DMSO. Fluorescence spectra were recorded using a Jasco FP 6500 spectrofluorometer. Thermal behavior (TG and DTA) was analyzed using a Labsys 1200 Setaram instrument, with a sample average mass of 12 mg, over the temperature range of 20–1000 °C, with a heating rate of 10 °C/min; measurements were carried out in a synthetic air atmosphere with a flow rate of 16.66 cm^3^/min by using alumina crucible. For high-resolution mass spectrometry (HRMS) the complexes were dissolved in DMSO and 1:10 (*v*:*v*) dilutions in MeOH were injected and analyzed on LTQ-Orbitrap Velos Pro with a nanoESI interface. The instrument was controlled using LTQ Tune v2.7 with manual data acquisition.

### 3.4. Geometry Optimization Study

With the aid of the Gaussian 09 software (version A.02) [110], the optimized structure of **Sm oxo** were obtained using the B3LYP DFT functional; the small core potential ECP52MWB was used for Sm and 6-31G(d,p) basis set was used for all H, C, O atoms. Doublet spin multiplicity was used, and the self-consistent field (SCF) method was employed in order to obtain the optimized geometries corresponding to the minimum on the potential energy surface. The optimized geometries were computed under no symmetry restrictions and the absence of imaginary frequencies in the vibrational analysis was verified in order to confirm that each structure corresponds to a true minimum. Gauss View 6.0.16 [111] has been used for data visualization and Mercury 4.0 [112] software for image capture.3.4. Biological studies.

#### 3.4.1. Cytotoxicity

The cytotoxicity assay is based on the ability of metabolically active cells to reduce MTS, a yellow tetrazolium salt, to the colored formazan, soluble in the culture medium; the concentration of formazan can be spectrophotometrically determined. A total of 15 × 10^3^ cells/well were cultured in 100 µL culture medium; culture supernatants were discarded and then cells were treated for 24 h and 48 h, respectively, with increasing concentrations of drugs. At the end of the incubation time, 20 µL reagent were added to each well, reagent containing MTS, [3-(4,5-dimethylthiazol-2-yl)-5-(3-carboxymethoxy-phenyl)-2-(4-sulfophenyl)-2*H*-tetrazolium, inner salt], and phenazine ethosulfate (PES); PES is a cationic dye with high chemical stability that binds to MTS and forms a stable solution. The plates were incubated with the coloring solution for 4 h at 37 °C, while being mildly agitated every 15 min. The reduced MTS to formazan was spectrophotometrically measured at λ = 492 nm. The test was run on two cancer cell lines, LoVo and MDA-MB 231, and on a normal cell line, HUVEC. Data were expressed as cell viability in comparison with untreated cells considered 100% viable, using the following formula:

(1) $$\mathrm{Cell}\;\mathrm{viability}\;\left(\%\right)=\frac{\mathrm{absorbance}\;\mathrm{of}\;\mathrm{treated}\;\mathrm{cells}\;-\;\mathrm{absorbance}\;\mathrm{of}\;\mathrm{culture}\;\mathrm{medium}}{\mathrm{absorbance}\;\mathrm{of}\;\mathrm{untreated}\;\mathrm{cells}-\;\mathrm{absorbance}\;\mathrm{of}\;\mathrm{culture}\;\mathrm{medium}}\;\times \;100$$

Cell viability data were obtained in triplicate (n = 3) and expressed as mean values ± standard deviations (SD). Statistical analysis was carried out using one-way analysis of variance (ANOVA) and Tukey’s test; a *p* value ≤ 0.05 was considered statistically significant. A parallel assay for the evaluation of DMSO cytotoxicity was conducted using the same experimental conditions; no cell cytotoxicity was observed for concentrations lower than 1%.

All assays were performed in 96-well microtiter plates with flat bottom (Falcon), using CellTiter 96 Aqueous One Solution Cell Proliferation Assay (Promega), an MTS-based colorimetric assay. The formazan was spectrophotometrically measured using the Tecan GENios Spectrophotometer.

#### 3.4.2. Studies on DNA Binding

The stability of the new complexes was tested before the study of interactions with DNA and proteins. An amount of 10^−3^ M solutions of the tested compounds were prepared in DMSO; further dilution of the samples was carried out in Tris-HCl buffer in order to obtain solutions of 40 μM. The UV-visible spectra were recorded at different time intervals (t = 0, 10, 20, 30, 60, 120 min.), in order to observe the possible variation of the absorbances; a control sample containing only DMSO and buffer was used.

##### UV-Vis Spectra

DNA binding studies were carried out by recording the UV-Vis spectra of the 1.8 mL samples containing 20 µM of the studied complex or free ligand and increasing amounts (0–40 µM with an increment of 5 µM) of DNA stock solution. The samples were prepared in Tris-HCl buffers (5 mM Tris-HCl/50 mM NaCl with a pH adjusted to 7.4 using a 0.1 M HCl solution). The concentration of the DNA stock solution was determined by measuring the absorption intensity at 260 nm and using a value of 6600 M^−1^ cm^−1^ for the molar extinction coefficient. Another necessary step was confirming that the DNA stock solution was sufficiently free of protein by calculating the ratio between the absorption at 260nm and 280nm, respectively, a ratio which had to be between 1.8 and 2 [98]; the value obtained was 1.8. These studies were carried out using a Jasco 650 spectrophotemeter, in 1 cm quartz cells, at room temperature. Three control samples were used, one containing DNA and DMSO, one with the studied complex and the last one with DMSO.

Equation (2) was used to calculate the binding constant (**K_b_**).

The equation used to evaluate the data is:

(2) $$\frac{\left[DNA\right]}{\varepsilon_{a}-\varepsilon_{f}}=\frac{\left[DNA\right]}{\varepsilon_{b}-\varepsilon_{f}}+\frac{1}{K_{b}\left(\varepsilon_{b}-\varepsilon_{f}\right)}$$

where **[DNA]** is the concentration of DNA in the sample, expressed in nucleobases, **ε_a_, ε_b_, ε_f_,** are the apparent absorption coefficient, the extinction coefficient for the complex in totally bound form and the extinction coefficient for the free complex, respectively, and **K_b_** is the intrinsic binding constant. **K_b_** was determined from the ratio of the slope (**1**/(**ε_b_** − **ε_f_**)) to the intercept (**1**/**K_b_**(**ε_b_** − **ε_f_**)) [113].

##### Competitive Binding Assay with Ethidium Bromide (EB) through Fluorescence Spectroscopy

Competitive binding studies were carried out on a Jasco FP 6500 fluorometer at room temperature, using quartz cells of 1 cm path-length. The fluorescence spectra were recorded on 1.6 mL samples containing 10 µM CT-DNA and 2 µM EB in the absence and presence of increasing amounts (0–40 µM with an increment of 5 µM) of complexes and free ligands, respectively, after being kept in contact for 10 min at room temperature, under continuous stirring and in the dark. The excitation wavelength used was 500 nm and the spectra was recorded in the 520–800 nm range. Two control samples were used, the first one containing DMSO, DNA and EB and the second one just the complex.

Data from the assay can be interpreted using the classical Stern-Volmer equation (Equation (3)) [114]:

(3) $$\frac{F_{0}}{F}=1+\;K_{SV}\cdot \left[Q\right]$$

where **F_0_** and **F** are the fluorescence intensities of the EB-DNA samples in the absence and presence of the tested compounds and **[Q]** is the concentration of the compound; **K_SV_** is the Stern-Volmer constant and can be calculated from the plot of **F_0_/F** vs. **[Q]** as the slope.

The ratio of binding relative to the control sample exposed only to DMSO was plotted against the logarithm of concentration. The data points were fitted using the modified Hill function with offset (Origin^®^) (Equation (4)):

(4) $$y=A_{1}+\frac{(A_{2}-A_{1})\times x^{n}}{K_{50}{}^{n}+x^{n}}$$

where **y** is the ratio of binding (**F/F_0_**) and **x** is the concentration, **A_1_** is the minimum of **y** values, **A_2_** is the maximum of **y** values, **K_50_** is the concentration corresponding to 50% binding and **n** is the Hill coefficient [115].

#### 3.4.3. Interactions with Human Serum Albumin (HSA) and Apo-Transferrin (apo-Tf)

##### Studies on Fluorescence Quenching Mechanism

Fluorescence spectra of HSA and apo-Tf in the presence and absence of the studied compounds were recorded using a Jasco FP 6500 spectrofluorometer, in quartz cells of 1 cm path-length. Some 2.5 mL samples containing 2.5 µM HSA or 1 µM apo-Tf, respectively, were titrated by successively adding 5 µL of the tested compound, corresponding to an increment of 1 µM, ranging from 0–8 µM. The samples were shaken for 3 min at room temperature and the corresponding spectra were recorded in the emission wavelength ranges 300–500 nm (HSA) and 305–500 nm (apo-Tf), using the excitation wavelengths of 280 nm (HSA) and 295 nm (apo-Tf). Experiments were carried out in Tris-HCl buffer at pH = 7.4 (5 mM Tris-HCl/50 mM NaCl for HSA and 5 mM Tris-HCl/50 mM NaCl/25 mM NaHCO_3_ for apo-Tf, respectively). Solutions containing the protein and DMSO and, respectively, the tested compounds alone, at the concentrations used in this study, were used as controls. The modified Stern-Volmer equation has been used to calculate the Stern-Volmer constant (Equation (5)):

(5) $$\frac{F_{0}}{F_{0}-F}=\frac{1}{f_{a}\;K_{SV}\left[Q\right]}+\;\frac{1}{f_{a}}$$

where **F_0_** and **F** are the fluorescence intensities in the absence and presence, respectively, of the tested compounds, **f_a_** is the fraction of fluorophore accessible to the quencher (the tested compounds), **[Q]** is the concentration of the quencher and **K_SV_** (M^−1^) is the Stern-Volmer constant; from the plot of **F_0_/(F_0_-F)** against **1/[Q]**, **K_SV_** and **f_a_** can be determined as the slope and the y intercept, respectively (Equation (6)):

(6) $$K_{SV}=\;K_{q}\tau_{0}$$

where **K_q_** (M^−1^ s^−1^) is the bimolecular quenching rate constant and **τ_0_** is the lifetime of the fluorophore in the absence of the studied compounds [109,116]. The value of **K_q_** is useful when trying to determine whether the type of mechanism underlying the quenching phenomenon is static or dynamic [98].

The affinity constant (**K_a_**) and number of binding sites (**n**) have been calculated using the following equation (Equation (7)):

(7) $$\mathrm{lg}\frac{F_{0}-F}{F}=\;\mathrm{lg}K_{a}+n\mathrm{lg}Q$$

In order to calculate the binding affinity, **K_d_**, the following quadratic function can be used (Equation (8)):

(8) $$\frac{F_{0}-F}{F_{0}-F_{b}}=\;\frac{{\left[P\right]}_{t}+\left[Q\right]+K_{d}-\sqrt{{\left({\left[P\right]}_{t}+\left[Q\right]+K_{d}\right)}^{2}-4{\left[P\right]}_{t}\left[Q\right]}}{2{\left[P\right]}_{t}}$$

where **F** and **F_0_** are the fluorescence intensities of the protein in the presence and absence of the quencher, respectively, **F_b_** is the fluorescence of a fully bound protein–quencher, **[P]_t_** is the total concentration of the protein, and **[Q]** is the concentration of added quencher. To quantify the binding affinities, each binding isotherm was plotted as a function of bound to free protein (**F/F_0_**) versus the total concentration of the quencher in solution. Then, the isotherms were fitted to the following nonlinear regression function (Equation (9)):

(9) $$\frac{F}{F_{0}}=\;F_{1}+\left(F_{2}-F_{1}\right)\frac{K_{d}^{n}}{K_{d}^{n}+{\left[Q\right]}^{n}}$$

where **n** denotes the Hill coefficient, and **F_2_** and **F_1_** are the vertical and horizontal asymptotes, respectively. The **K_d_** in Equation (9) is derived from the quadratic binding function (Equation (8)) [115]. Data were obtained in triplicate, averaged and expressed as mean ± standard deviation (SD). All graphs fitting models were defined and developed applying OriginPro 2018.

##### Studies on Conformational Changes of HSA and Apo-Tf Conformation Due to the Interaction with the Tested Compounds

These studies were carried out by measuring the synchronous fluorescence spectra of the two proteins in the presence and absence of the studied complexes and free ligands; the spectra were recorded on the same samples as for the binding studies, but using Δλ = 60 nm and Δλ = 15 nm, respectively, in the 250–400 nm interval. Two control samples were used, one containing the studied protein and DMSO and the other containing the complex.

## 4. Conclusions

Six new neutral complexes of oxolinic acid with rare-earth metal ions have been synthesized and further characterized. Structural characterization has comprised data obtained by means of elemental analysis, spectroscopy studies (UV-VIS-NIR, FT-IR, high-resolution mass spectrometry) and thermal analysis. The cytotoxic effects of the complexes, as well as the free ligand, were tested on two cancer cell lines, MDA-MB 231 (human breast adenocarcinoma), and LoVo (human colon adenocarcinoma) and the normal human umbilical vein endothelial cells (HUVEC) cell line; the complexes display very low cytotoxic effects towards the HUVEC cell line and satisfactory anticancer activities, some of the complexes presenting IC_50_ values being lower than the positive controls.

Regarding the DNA interaction potential, K_b_ values are consistent with moderate interactions; competitive binding assay also suggests a moderate capacity for displacing EB from EB-DNA complex. HSA and apo Tf binding studies reveal good binding affinities for both proteins, with relatively high binding constants. The complexes displayed an overall higher affinity towards HSA, the interaction occurring via a static quenching mechanism.

The potential use as anticancer agents of the rare-earth metal complexes could be further explored in future studies by obtaining ternary complexes with oxolinic acid and various ligands suitable for DNA intercalation. This would be an opportunity to explore ways of altering the DNA-interaction mechanism, with the aim of improving their antitumoral properties. Another direction of expanding this study is to investigate the magnetic properties of the lanthanide complexes and the possibility to be used as MRI contrast agents for theranostic applications.

## Figures and Tables

**Table 1:** Assignments of the recorded bands in the IR spectra of the studied compounds.

Oxo	Oxo^−^Na^+^	Y oxo	La oxo	Sm oxo	Eu oxo	Gd oxo	Tb oxo	Assignments
-	-	3725 vw 3399 w	3622 w 3359 w	3345 wb	3389 wb	3378 wb	3391 wb	ν(O-H); COOH, H_2_O
3061 w	3280 w,b	3056 w	3057 w	3059 w	3059 w	3060 w	3057 w	ν(C-H) aromatic
2983 w 2930 w	2975 w 2910 w	2974 w 2925 w	2909 w	2916 w	2984 w	-	2976 w 2921 w	ν (C-H) aliphatic
1698 ms	-	-	-	-	-	-		ν(C=O) COOH
-	1634 vs	1631 s	1632	1634	1633	1633	1632 s	ν as (COO−)
1632 ms	1592 s	1594 s	1577 s	1576 s	1578 s	1579 s	1582 s	ν(C=O) pyridone
1438 vs	1498 ms 1473 s	1497 s 1469 s	1496 w 1469 vs	1495 m 1465 s	1493 s 1479 s 1465 vs	1496 s 1466 vs	1496 s 1468 vs	ν (-CH); δ (-CH_2_)
1384 m	1391 s	1387 m	1413 m	1412 m	1416 m	1416 m	1387 m	δ (C-H) methyl group
	1338 vs	1339 s	1341 s	1340 s	1340	1341 m	1336 s	ν s(COO-)
-	297	292	292	294	293	292	295	Δ = ν as (COO-)- ν s(COO-)
1147 m	1188 ms	1192 m	1193	1191 m	1194 m	1194 m	1193 m	ν (C-N)
1075 m 1093 m	1091 mw	1088 w	1089 w	1084 w	1092 w	1092 w	1088 w	ν (O-CH_2_-O)
772 m 754 m	768 m 742	770 s 744	771 744	772 s 745 m	773 ms 744 m	773 ms 744 m	770 ms 743 m	δ (COO-)
645 s	639 m	649 m	640 m	646 m	643 m	644 m	647 m	ring deformation
606 m	607 m	611 m	608 m	610 m	609 m	609 m	610 m	
556 m	547 m	559 m	553 m	557 m	556 m	556 m	558 m	
-	513 m	-	-	-	-	-	-	
499 m	499 m	500 m	497 s	500 m	-	-	-	
448 w	459 w	-	-	-	453 m	445 m	459 mw	
431 w	-	428 m	431 w	-	-	-	-	
418 m	416 m	416 m	-	-	-	413 ms	412 m	
-	-	-	408 s	407 s	410 s	-	-	
-	-	401 m	400 s	-	-	-	-	
-	-	512 s	-	513 s	512 s	511 s	511 s	ν (M-O)
-	-	-	485 s	484 m	483 s	483 s	499 s	
-	-	468 w	456 m	456 m	-	-	-	
-	-	-	-	-	421 s	423 m	422 m	

**Table 2:** Thermal decomposition data (in air flow) for the complexes.

Complex	Step	Thermal Effect	Temperature Range/°C	Δ*m*_exp_/%	Δ*m*_calc_/%
**Y oxo**	1	Endothermic	125–280	2.40	2.80
	2	Exothermic	280–900	79.90	79.70
	Residue (1/2 Y_2_O_3_)			17.70	17.50
**La oxo**	1	Endothermic	75–150	2.70	2.60
	2	Exothermic	150–900	73.50	73.90
	Residue (1/2 La_2_O_3_)			23.80	23.50
**Sm oxo**	1	Endothermic	75–220	4.60	5.00
	2	Exothermic	220–950	71.40	70.90
	Residue (1/2 Sm_2_O_3_)			24.00	24.10
**Eu oxo**	1	Endothermic	125–240	2.60	2.50
	2	Exothermic	240–875	72.40	72.60
	Residue (1/2 Eu_2_O_3_)			25.00	24.90
**Gd oxo**	1	Endothermic	110–250	3.80	3.70
	2	Exothermic	250–880	70.80	71.20
	Residue (1/2 Gd_2_O_3_)			25.40	25.10
**Tb oxo**	1	Endothermic	60–160	3.60	3.70
	2	Exothermic	280–980	70.50	70.50
	Residue (1/4 Tb_4_O_7_)			25.90	25.80

**Table 3:** Geometric parameters: bond lengths, bond angles, dihedral angles, charge density, total energy of **Sm oxo** as resulted from DFT calculations at the B3LYP/ ECP52MWB (Sm)/ 6-31G(d,p) (H, C, O atoms) level in Gaussian 09 software package.

**Bond Length (Å)**	**Sm oxo**	**Charge**	**Sm oxo**
Sm-O2	2.517	Sm	1.092
Sm-O3	2.506	O2	−0.586
Sm-O24	2.582	O3	−0.536
Sm-O25	2.519	O4	−0.504
Sm-O46	2.557	O24	−0.594
Sm-O48	2.664	O25	−0.530
**Bond angle (°)**	**Sm oxo**	O26	−0.498
O2-Sm-O3	69.233	O46	−0.480
O24-Sm-O25	68.477	O48	−0.593
O46-Sm-O48	102.286	N5	−0.435
O46-Sm-O25	53.623	N7	−0.437
O3-Sm-O48	134.706	C5	0.116
O2-Sm-O24	110.618	C9	0.116
Sm-O25-C28	119.094	C12	−0.023
Sm-O3-C6	144.027	**Total energy** (a.u.)	−2045.743
**Dihedral angle**	**Sm oxo**		
O46-Sm-O25-C28	87.515		
O48-Sm-O3-C6	−176.277		
O48-Sm-O24-C32	−116.492		
O46-Sm-O2-C10	−130.417		
Sm-O25-C28-O26	−101.279		
Sm-O3-C6-O4	169.368		

**Table 4:** IR selected data for the **Sm oxo** complex (experimental vs. calculated for the optimized structure.

Assignments	Experimental Data	B3LYP/6-31G(d,p)/ ECP52MWB
ν(O-H); COOH, H_2_O	3345 w, b	3617 w 3309 s
ν(C-H) aromatic	3059 w	3134 w 3097 w
ν(C-H) aliphatic	2916 w	3060 w 3059 w 3043 w 3034 w
ν as(COO-)	1634	1758 s 1695 s
ν(C=O) pyridone	1576 s	1608 s
ν(-CH); δ(-CH2)	1495 m 1465 s	1527 s 1510 m
δ(C-H) methyl group	1412 m	1433 w 1428 w
ν s(COO-)	1340 s	1360 s
ν(C-N)	1191 m	1224 w
ν (O-CH_2_-O)	1084 w	1073 w
δ(COO-)	772 m 745 m	784 w 757 w
ring deformation	646 m	637 m
	610 m	614 w
	557 m	517 w
	500 m	503 w
	407 s	-
ν (M-O)	513 s	513 w
	484 m	503 w
	456 m	425 w
	513 s 484 m 456 m	513 w 503 w 425 w

**Table 5:** IC_50_ values (μM) for **oxo**, **M oxo** and cisplatin/adryamicin.

Compound	IC_50_ (μM)		
	LoVo (Human Colon Adenocarcinoma)	MDA-MB 231 (Human Breast Adenocarcinoma)	HUVEC (Normal, Human Umbilical Vein Endothelial Cells)
**oxo**	>200 μM	43.49 ± 4.94	>200 μM
**Y oxo**	90.41 ± 9.30	33.22 ± 14.92	>200 μM
**La oxo**	52.10 ± 5.54	>100 μM	>200 μM
**Sm oxo**	41.51 ± 15.28	40.42 ± 6.27	>200 μM
**Eu oxo**	106.60 ± 16.04	73.65 ± 19.80	>200 μM
**Gd oxo**	56.49 ± 3.83	73.79 ± 18.50	>200 μM
**Tb oxo**	40.59 ± 7.43	>200 μM	>200 μM
**Cis-Pt**	40.15 ± 13.94	-	28.46 ± 6.28
**ADR**	-	7.85 ± 0.70	-

IC_50_ value represents the concentration of the tested compound required to inhibit 50% of cell growth with respect to control sample, in the absence of the compound. Cytotoxicity was assessed by MTS assay after 48 h of incubation with each compound. Data obtained are based on the average of three independent experiments and the reported errors are the corresponding standard deviations.

**Table 6:** Binding constants (K**_b_**), which characterize the interactions of the tested compounds with DNA, and quenching constants (K**_SV_**) for the EB-DNA-tested compounds systems; binding constants characterizing the DNA-tested compounds interactions calculated from the EB displacement assays.

	K_b_ (L∙mol^−1^)	K_sv_ (×10^4^)	EB Displacement Assay	
Compound			K_50_ (µM)	n
**oxo**	(0.46 ± 0.14) × 10^4^	(1.05 ± 0.02) × 10^4^	42.07 ± 18.48	2.98 ± 1.14
**Y-oxo**	(4.02 ± 1.41) × 10^5^	(8.38 ± 0.29) × 10^3^	28.07 ± 4.44	1.92 ± 0.45
**La-oxo**	(2.57 ± 0.17) × 10^5^	(1.27 ± 0.03) × 10^4^	37.05 ± 8.86	3.44 ± 0.94
**Sm-oxo**	(9.33 ± 1.46) × 10^4^	(1.21 ± 0.03) × 10^4^	41.55 ± 19.32	2.44 ± 0.82
**Eu-oxo**	(10.72 ± 2.47) × 10^5^	(1.26 ± 0.02) × 10^4^	40.15 ± 11.82	2.65 ± 0.73
**Gd-oxo**	(5.77 ± 1.07) × 10^5^	(1.18 ± 0.01) × 10^4^	47.75 ± 25.22	1.66 ± 0.47
**Tb-oxo**	(9.84 ± 3.83) × 10^5^	(1.18 ± 0.00) × 10^4^	20.28 ± 1.15	2.68 ± 0.19

**Table 7:** Binding constants and thermodynamic parameters calculated for the interaction of the complexes with apo-Tf and HSA, respectively.

Complex	T (K)	Ksv (M^−1^)	Kq (M^−1^∙s^−1^)	Ka (M^−1^)	n (Number of Binding Sites)	Kd (μM)	n (Hill Coefficient)
**Apo-Tf**							
**oxo**	298	(17.45 ± 0.95) × 10^4^ (f_a_ = 0.36±0.01)	17.45 × 10^12^	(0.73 ± 0.07) × 10^3^	0.67 ± 0.00	8.11 ± 7.34	1.89 ± 1.35
**Y-oxo**	298	(6.52 ± 0.56) × 10^4^ (f_a_ = 1.15 ± 0.07)	6.52 × 10^12^	(4.40 ± 1.09) × 10^4^	0.94 ± 0.01	8.34 ± 1.71	1.58 ± 0.23
**La-oxo**	298	(14.13 ± 1.46) × 10^4^ (f_a_ = 0.68 ± 0.03)	14.13 × 10^12^	(0.53 ± 0.23) × 10^4^	0.77 ± 0.03	8.89 ± 2.11	1.71 ± 0.21
**Sm-oxo**	298	(9.34 ± 0.66) × 10^4^ (f_a_ = 0.85 ± 0.05)	9.34 × 10^12^	(4.16 ± 1.09) × 10^4^	0.95 ± 0.02	5.73 ± 0.55	2.03 ± 0.32
**Eu-oxo**	298	(16.55 ± 0.94) × 10^4^ (f_a_ = 0.81 ± 0.02)	16.55 × 10^12^	(2.74 ± 0.41) × 10^4^	0.88 ± 0.01	8.27 ± 4.17	0.95 ± 0.21
**Gd-oxo**	298	(17.51 ± 1.06) × 10^4^ (f_a_ = 0.73 ± 0.02)	17.51 × 10^12^	(0.58 ± 0.25) × 10^4^	0.76 ± 0.03	8.80 ± 6.57	1.24 ± 0.45
**Tb-oxo**	298	(19.45 ± 1.10) × 10^4^ (f_a_ = 0.77 ± 0.01)	19.45 × 10^12^	(1.28 ± 0.17) × 10^4^	0.81 ± 0.01	5.57 ± 1.22	1.57 ± 0.33
**HSA**							
**oxo**	298	(1.95 ± 0.38) × 10^4^ (f_a_ = 1.92 ± 0.35)	1.95 × 10^12^	(1.08 ± 0.22) × 10^4^	0.90 ± 0.01	7.26 ± 2.66	1.62 ± 0.39
**Y-oxo**	298	(10.53 ± 0.95) × 10^4^ (f_a_ = 1.07 ± 0.05)	10.53 × 10^12^	(2.04 ± 0.74) × 10^5^	1.44 ± 0.03	6.30 ± 2.47	1.52 ± 0.47
**La-oxo**	298	(6.82 ± 0.74) × 10^4^ (f_a_ = 1.26 ± 0.11)	6.82 × 10^12^	(5.80 ± 2.01) × 10^5^	1.14 ± 0.02	6.22 ± 1.05	1.74 ± 0.21
**Sm-oxo**	298	(6.56 ± 0.90) × 10^4^ (f_a_ = 1.44 ± 0.15)	6.56 × 10^12^	(4.28 ± 2.12) × 10^5^	1.11 ± 0.04	4.69 ± 0.87	1.55 ± 0.37
**Eu-oxo**	298	(11.25 ± 0.92) × 10^4^ (f_a_ = 0.94 ± 0.04)	11.25 × 10^12^	(4.75 ± 0.80) × 10^5^	1.11 ± 0.01	5.56 ± 0.86	1.33 ± 0.18
**Gd-oxo**	298	(5.77 ± 0.84) × 10^4^ (f_a_ = 1.39 ± 0.15)	5.77 × 10^12^	(1.98 ± 0.82) × 10^5^	1.06 ± 0.03	5.32 ± 0.69	1.81 ± 0.26
**Tb-oxo**	298	(2.84 ± 0.70) × 10^4^ (f_a_ = 2.13 ± 0.45)	2.84 × 10^12^	(6.83 ± 1.33) × 10^5^	1.18 ± 0.01	5.97 ± 0.89	1.58 ± 0.20

## Supplementary Materials

The following are available online, Table S1: Wavelengths and absorbance (A) values observed in the UV-Vis-NIR spectra of oxolinic acid and complexes, Table S2: Absorbances of tested compounds in UV-vis, in DMSO-TrisHCl buffer, [compound]= 40μM, Figure S1: FT-IR spectrum of oxolinic acid, Figure S2: FT-IR spectrum of sodium oxolinate, Figure S3: FT-IR spectrum of Y oxo, Figure S4: FT-IR spectrum of La oxo, Figure S5: Experimental vs. predicted IR spectrum of Sm oxo, Figure S6: FT-IR spectrum of Eu oxo, Figure S7: FT-IR spectrum of Gd oxo, Figure S8: FT-IR spectrum of Tb oxo, Figure S9: MS spectrum for Y oxo (left) and La oxo (right), Figure S10: MS, MS/MS and MS/MS/MS spectra for Sm oxo, Figure S11: MS, MS/MS and MS/MS/MS spectra for Eu oxo, Figure S12: MS, MS/MS and MS/MS/MS spectra for Gd oxo, Figure S13: MS spectrum for Tb oxo, Figure S14: Stability assay-UV-vis spectra of the complexes in DMSO-Tris Cl buffer mixture, Figure S15: The variation of the absorbances of the studied complexes during the stability assay, in DMSO-Tris-HCl buffer mixture, Figure S16: Cell viability (%) after 24h of incubation with tested compounds: A. on MDA-MB 231 cell line, B. LoVo cell line, Figure S17: Cell viability (%) after 48h of incubation with tested compounds: A. on MDA-MB 231 cell line, B. LoVo cell line, Figure S18: Normal cell viability (%) after A. 24h and B. 48h of incubation with tested compounds, Figure S19: Absorption spectra of the tested compounds in the absence and presence of increasing amounts of DNA. [compound] = 20 μM; [DNA] = 0, 5, 10, 15, 20, 25, 30, 35, 40 μM. The arrows show the absorption changes upon increasing the DNA concentration, Figure S20: Representation of the DNA-binding constant (K_b_) of the studied compounds, Figure S21: Fluorescence spectra of the EB - DNA system in the absence and presence of increasing amounts of the tested compounds. λex = 500 nm, [EB] = 2 μM, [DNA] = 10 μM, [compound] = 0, 5, 10, 15, 20, 25, 30, 35, 40 μM. Arrows indicate the changes in fluorescence intensities upon increasing the concentrations of the tested compounds, Figure S22: Graphical representation of the KSV constants of the studied compounds, Figure S23: Representation of the K_50_ constant of the studied compounds, Figure S24: Changes in fluorescence intensity of free HSA vs. compound-HSA systems; the black arrows indicate a decrease of the intensity upon the addition of increasing amounts of compound; [HSA] = 2.5 μM, [compound] = 0, 1, 2, 3, 4, 5, 6, 7, 8 μM, Figure S25: Changes in fluorescence intensity of free apo Tf vs. compound-apo Tf systems; the black arrows indicate a decrease of the intensity upon the addition of increasing amounts of compound. [apo-Tf] = 1 μM, [compound] = 0, 1, 2, 3, 4, 5, 6, 7, 8 μM, Figure S26: Changes in the peak areas for the compound-HSA systems. [HSA] = 2.5 μM, [compounds] = 0, 1, 2, 3, 4, 5, 6, 7, 8 μM, Figure S27: Variation of the apo-Tf fluorescence peak area upon adding increasing amounts of studied compounds. [apo-Tf] = 1 μM, [compound] = 0, 1, 2, 3, 4, 5, 6, 7, 8 μM, Figure S28: Classical (left) and modified (right) Stern-Volmer plots for each of the HSA interaction studies, Figure S29: Classical (left) and modified (right) Stern-Volmer plots for the apo-Tf interaction assay, Figure S30: Ka and Kd for each of the studied compound-HSA systems, Figure S31: Representation of Ka and Kd constants for each of the apo-Tf interaction systems, Figure S32: Synchronous spectra for the HSA interaction systems recorded at Δλ= 15 nm. [HSA] = 2.5 μM, [Sm oxo] = 0, 1, 2, 3, 4, 5, 6, 7, 8 μM, Figure S33: Synchronous spectra for the HSA interaction systems recorded at Δλ = 60 nm. [HSA] = 2.5 μM, [Sm oxo] = 0, 1, 2, 3, 4, 5, 6, 7, 8 μM, Figure S34: Synchronous spectra of the tested compounds - apo-Tf systems recorded at Δλ = 15 nm. [apo-Tf] = 1 μM, [compound] = 0, 1, 2, 3, 4, 5, 6, 7, 8 μM, Figure S35: Synchronous spectra of the tested compounds-apo-Tf interaction systems recorded at Δλ = 60 nm. [apo-Tf] = 1 μM, [compound] = 0, 1, 2, 3, 4, 5, 6, 7, 8 μM.
