# Supplementary material for: Rare-Earth Metal Complexes of the Antibacterial Drug Oxolinic Acid: Synthesis, Characterization, DNA/Protein Binding and Cytotoxicity Studies
Source: Molecules. 2020 Nov 19;25(22):5418. doi: 10.3390/molecules25225418 (PMC7699381; doi:10.3390/molecules25225418)
Supplement: Supplementary file 1 [file molecules-25-05418-s001.pdf]

Rare-earth metal complexes of the antibacterial drug oxolinic acid: Synthesis, characterization, DNA/protein binding and cytotoxicity studies

Ana-Madalina Maciuca[1], Alexandra-Cristina Munteanu[1*], Mirela Mihaila[2], Mihaela Badea[3], Rodica Olar[3], George Mihai Nitulescu[4], Cristian V. A. Munteanu[5], Marinela Bostan[2] and Valentina Uivarosi[1*]

[1]Department of General and Inorganic Chemistry, Faculty of Pharmacy, Carol Davila University of Medicine and Pharmacy, 6 Traian Vuia St, Bucharest 020956, Romania;
[2]Center of Immunology, Stefan S. Nicolau Institute of Virology, 285 Mihai Bravu Ave, Bucharest 030304, Romania
[3]Department of Inorganic Chemistry, Faculty of Chemistry, University of Bucharest, 90-92 Panduri Str, Bucharest 050663, Romania
[4] Department of Pharmaceutical Chemistry, Faculty of Pharmacy, Carol Davila University of Medicine and Pharmacy, 6 Traian Vuia Str, Bucharest 020956, Romania
[5] Romanian Academy-Institute of Biochemistry (IBAR), 296 Spl. Independenţei, 060031 Bucharest, Romania
* Correspondence: alexandra.ticea@umfcd.ro; valentina.uivarosi@umfcd.ro
Tel.: +4-021-318-0742; Fax: +4-021-318-0750

Abstract: "Drug repositioning" is a current trend which proved useful in the search of new applications for existing, failed, no longer in use or abandoned drugs, particularly when addressing issues such as bacterial or cancer cells resistance to current therapeutic approaches. In this context, six new complexes of the first-generation quinolone oxolinic acid with rare-earth metal cations (Y^{3+}, La^{3+}, Sm^{3+}, Eu^{3+}, Gd^{3+}, Tb^{3+}) have been synthesized and characterized. The experimental data suggest that the quinolone acts as a bidentate ligand, binding to the metal ion *via* the keto and carboxylate oxygen atoms; these findings are supported by DFT calculations for the Sm^{3+} complex. The cytotoxic activity of the complexes, as well as the ligand, has been studied on MDA-MB 231 (human breast adenocarcinoma) and LoVo (human colon adenocarcinoma) cell lines. UV-Vis spectroscopy and competitive binding studies show that the complexes display binding affinities (K_b) towards double stranded DNA in the range of 9.33×10^4 - 10.72×10^5. Major and minor groove-binding most likely play a significant role in the interactions of the complexes with DNA. Moreover, the complexes bind human serum albumin more avidly than apo-transferrin.

Keywords: drug repositioning; quinolone; oxolinic acid; rare-earth metal ions; anticancer; DNA binding; serum proteins.

**Table S1.** Wavelengths and absorbance (A) values observed in the UV-Vis-NIR spectra of oxolinic acid and complexes.	4
**Table S2**- Absorbances of tested compounds in UV-vis, in DMSO-TrisHCl buffer, [**compound**]= 40μM	20
**Figure S1.** FT-IR spectrum of oxolinic acid	5
**Figure S2.** FT-IR spectrum of sodium oxolinate	6
**Figure S3.** FT-IR spectrum of **Y oxo.**	7
**Figure S4.** FT-IR spectrum of **La oxo.**	8
**Figure S5.** Experimental *vs.* predicted IR spectrum of **Sm oxo.**	9
**Figure S6.** FT-IR spectrum of **Eu oxo.**	10
**Figure S7.** FT-IR spectrum of **Gd oxo.**	11
**Figure S8.** FT-IR spectrum of **Tb oxo.**	12
**Figure S9.** MS spectrum for **Y oxo** (left) and **La oxo (**right).	13
**Figure S10.** MS, MS/MS and MS/MS/MS spectra for **Sm oxo.**	14
**Figure S11.** MS, MS/MS and MS/MS/MS spectra for **Eu oxo**.	15
**Figure S12.** MS, MS/MS and MS/MS/MS spectra for **Gd oxo**.	16
**Figure S13.** MS spectrum for **Tb oxo**.	17
**Figure S14.** Stability assay-UV-vis spectra of the complexes in DMSO-Tris Cl buffer mixture.	18
**Figure S15.** The variation of the absorbances of the studied complexes during the stability assay, in DMSO-Tris-HCl buffer mixture.	19
**Figure S16.** Cell viability (%) after 24h of incubation with tested compounds: **A.** on MDA-MB 231 cell line, **B.** LoVo cell line.	20
**Figure S17.** Cell viability (%) after 48h of incubation with tested compounds: **A**. on MDA-MB 231 cell line, **B**. LoVo cell line.	21
**Figure S18.** Normal cell viability (%) after **A**. 24h and **B**. 48h of incubation with tested compounds.	21
**Figure S19.** Absorption spectra of the tested compounds in the absence and presence of increasing amounts of DNA. [**compound**] = 20 μM; [DNA] = 0, 5, 10, 15, 20, 25, 30, 35, 40 μM. The arrows show the absorption changes upon increasing the DNA concentration.	22
**Figure S20.** Representation of the DNA-binding constant (K_b) of the studied compounds	23
**Figure S21.** Fluorescence spectra of the EB - DNA system in the absence and presence of increasing amounts of the tested compounds. λ_{ex} = 500 nm, [EB] = 2 μM, [DNA] = 10 μM, [compound] = 0, 5, 10, 15, 20, 25, 30, 35, 40 μM. Arrows indicate the changes in fluorescence intensities upon increasing the concentrations of the tested compounds.	24
**Figure S22.** Graphical representation of the K_{SV} constants of the studied compounds.	25
**Figure S23.** Representation of the K_{50} constant of the studied compounds.	26

**Figure S24.** Changes in fluorescence intensity of free HSA *vs.* compound-HSA systems; the black arrows indicate a decrease of the intensity upon the addition of increasing amounts of compound; [**HSA**] = 2.5 μM, [**compound**] = 0, 1, 2, 3, 4, 5, 6, 7, 8 μM.	27
**Figure S25.** Changes in fluorescence intensity of free apo Tf *vs.* compound-apo Tf systems; the black arrows indicate a decrease of the intensity upon the addition of increasing amounts of compound. **[apo-Tf]** = 1 μM, [**compound**] = 0, 1, 2, 3, 4, 5, 6, 7, 8 μM.	28
**Figure S26.** Changes in the peak areas for the compound-HSA systems. [**HSA**] = 2.5 μM, [**compounds**] = 0, 1, 2, 3, 4, 5, 6, 7, 8 μM.	29
**Figure S27.** Variation of the apo-Tf fluorescence peak area upon adding increasing amounts of studied compounds. [**apo-Tf**] = 1 μM, [**compound**] = 0, 1, 2, 3, 4, 5, 6, 7, 8 μM.	30
**Figure S28.** Classical (left) and modified (right) Stern-Volmer plots for each of the HSA interaction studies.	31-33
**Figure S29.** Classical (left) and modified (right) Stern-Volmer plots for the apo-Tf interaction assay.	34-36
**Figure S30**. Ka and Kd for each of the studied compound-HSA systems.	37-39
**Figure S31.** Representation of Ka and Kd constants for each of the apo-Tf interaction systems.	40-42
**Figure S32.** Synchronous spectra for the HSA interaction systems recorded at $\Delta\lambda$= 15 nm. [**HSA**] = 2.5 μM, [**Sm oxo**] = 0, 1, 2, 3, 4, 5, 6, 7, 8 μM.	43
**Figure S33.** Synchronous spectra for the HSA interaction systems recorded at at $\Delta\lambda$= 60 nm. [**HSA**] = 2.5 μM, [**Sm oxo**] = 0, 1, 2, 3, 4, 5, 6, 7, 8 μM.	44
**Figure S34.** Synchronous spectra of the tested compounds - apo-Tf systems recorded at $\Delta\lambda$=15 nm. [**apo-Tf**] = 1 μM, [**compound**] = 0, 1, 2, 3, 4, 5, 6, 7, 8 μM.	45
**Figure S35.** Synchronous spectra of the tested compounds - apo-Tf interaction systems recorded at $\Delta\lambda$=60 nm. [**apo-Tf**] = 1 μM, [**compound**] = 0, 1, 2, 3, 4, 5, 6, 7, 8 μM.	46

Table S1. Wavelengths and absorbance (A) values observed in the UV-Vis-NIR spectra of oxolinic acid and complexes.

Compound	Acid oxolinic								
**λ(nm)**	270	365	-	-	-	1675	1725	-	-
**A**	0,457	0,731	-	-	-	0,201	0,165	-	-
**Sodium oxolinate**									
**λ(nm)**	265	340	1185	-	-	1475	1665	-	1950
**A**	0,629	0,787	0,046	-	-	0,086	0,117	-	0,253
**Y-oxo**									
**λ(nm)**	265	345	425	-	-	1455	1670	1945	-
**A**	0,496	0,654	0,105	-	-	0,077	0,096	0,174	-
**La-oxo**									
**λ(nm)**	265	345	-	-	1415	-	1685	1910	-
**A**	0,767	0,896	-	-	0,139	-	0,174	0,307	-
**Sm-oxo**									
**λ(nm)**	255	345	1090	1245	1390	1500	1555	1930	1940
**A**	0,905	1,002	0,219	0,259	0,239	0,254	0,235	0,302	0,303
**Eu-oxo**									
**λ(nm)**	270	345	370	-	-	1450	1675	1945	-
**A**	0,492	0,656	0,641	-	-	0,071	0,073	0,192	-
**Gd-oxo**									
**λ(nm)**	260	340	-	-	-	1450	1675	1940	1950
**A**	0,897	1,005	-	-	-	0,118	0,099	0,272	0,268
**Tb-oxo**									
**λ(nm)**	-	380	-	-	-	1455	1675	1825	1945
**A**	-	0.481	-	-	-	0.113	0.118	0.129	0.285

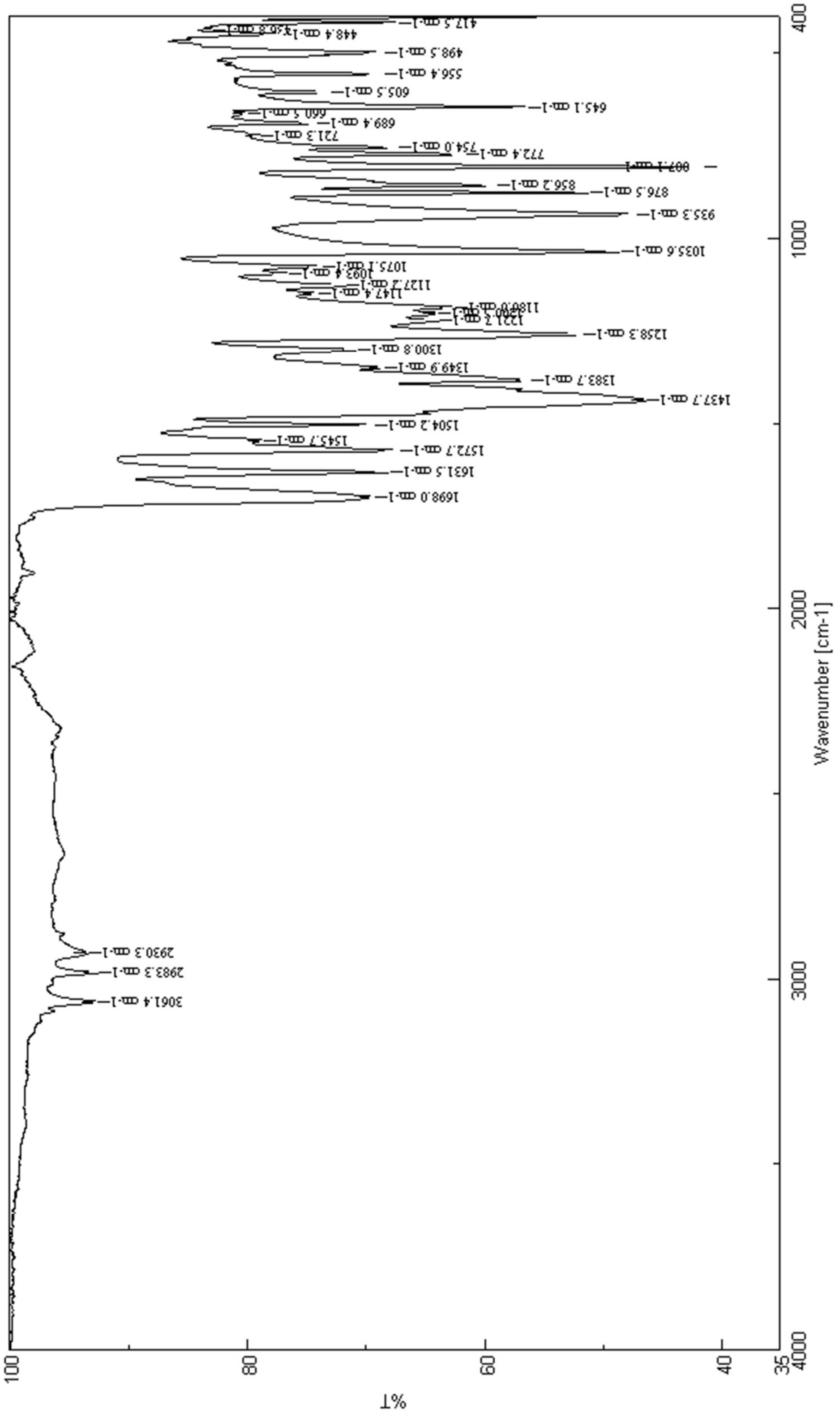

Figure S1. FT-IR spectrum of oxolinic acid.

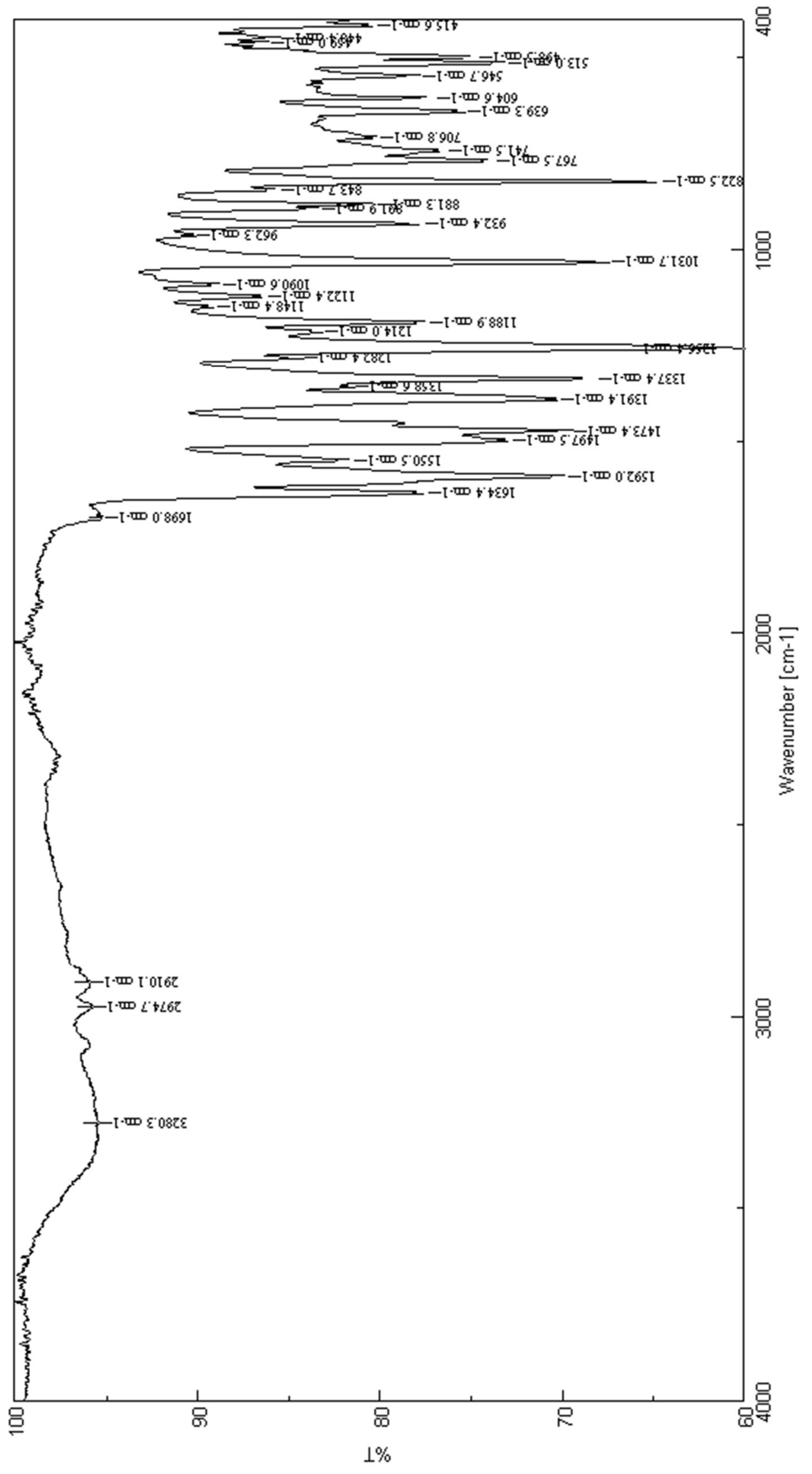

Figure S2. FT-IR spectrum of sodium oxolinate.

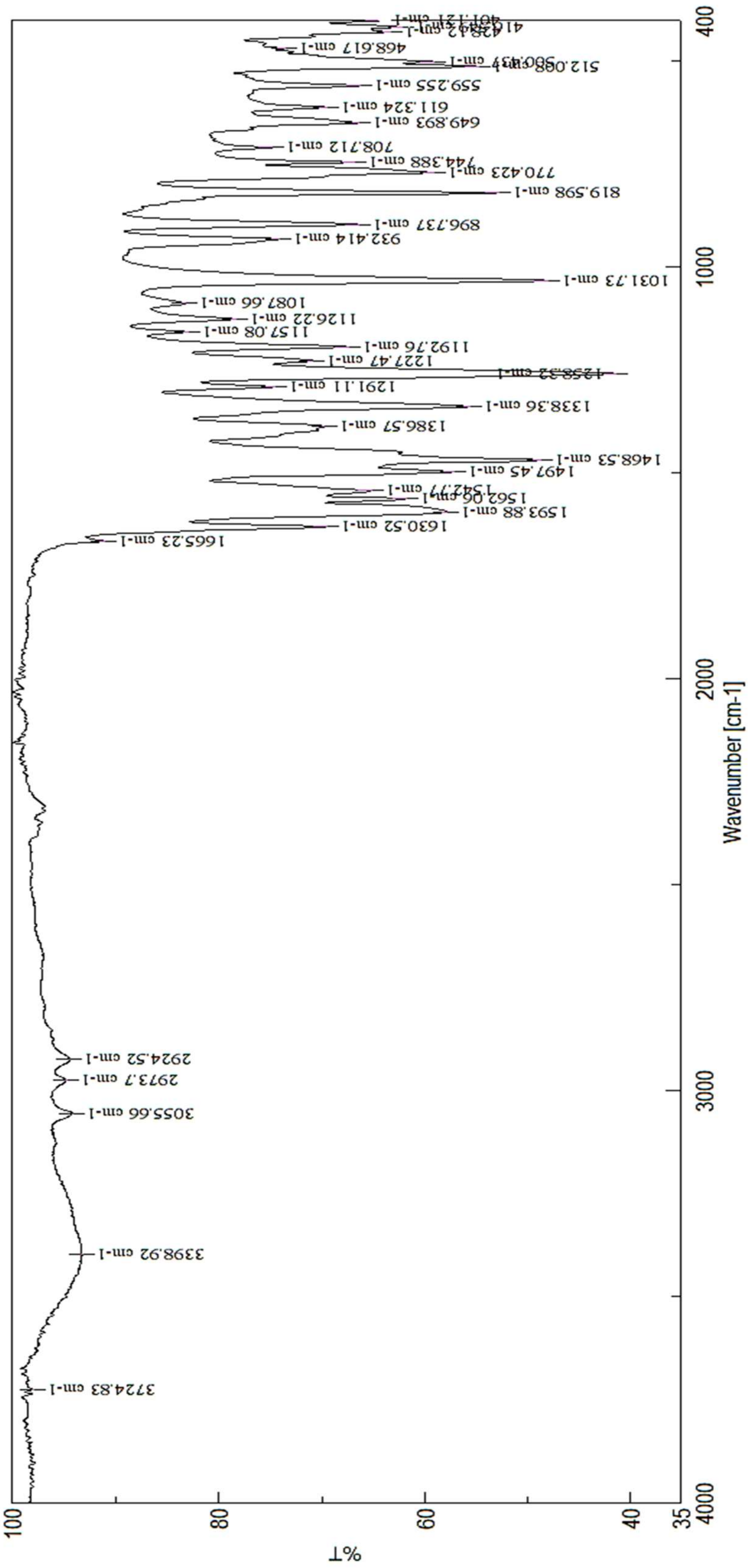

Figure S3. FT-IR spectrum of **Y oxo.**

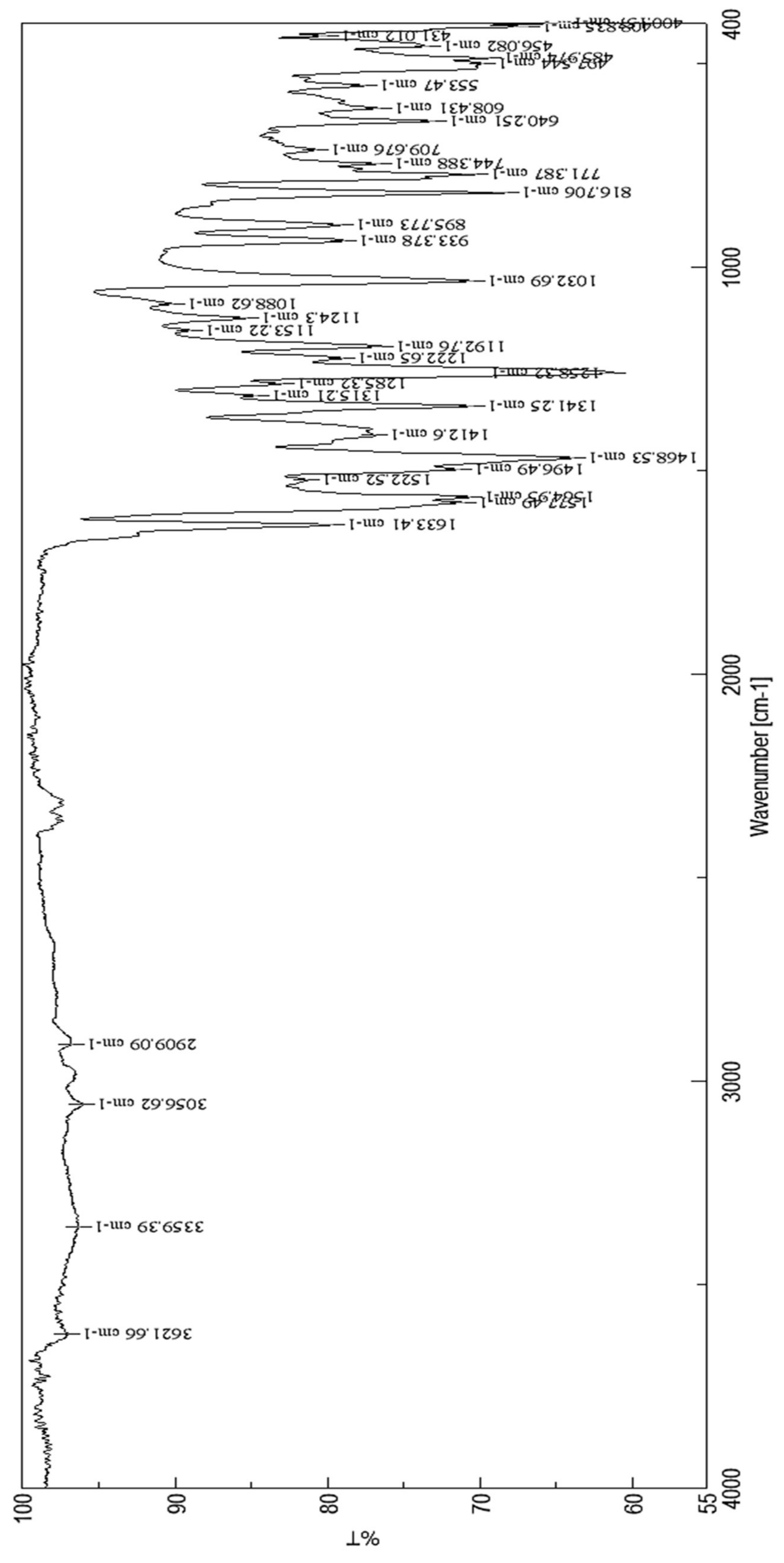

Figure S4. FT-IR spectrum of **La oxo.**

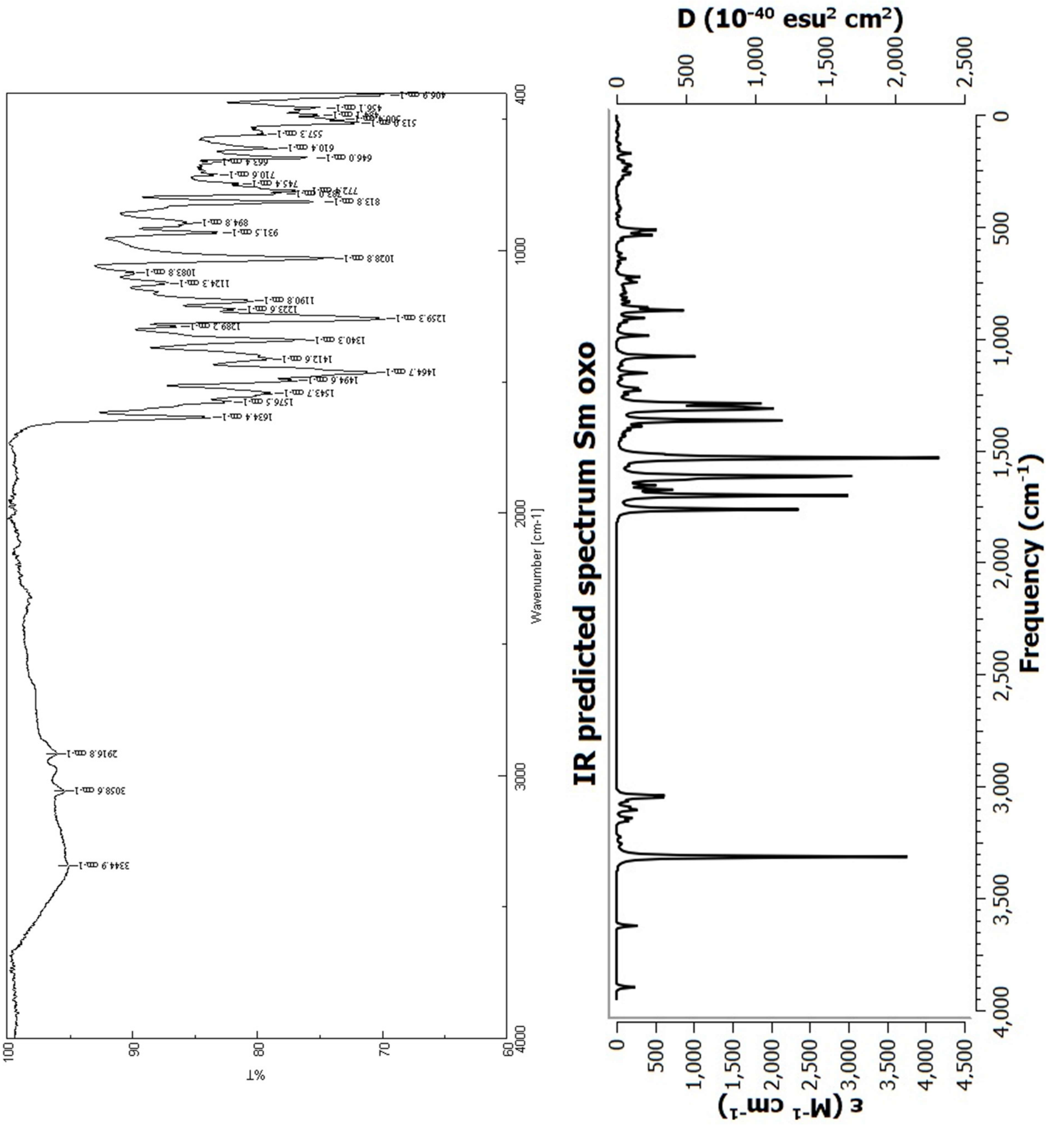

Figure S5. Experimental *vs.* predicted IR spectrum of **Sm oxo.**

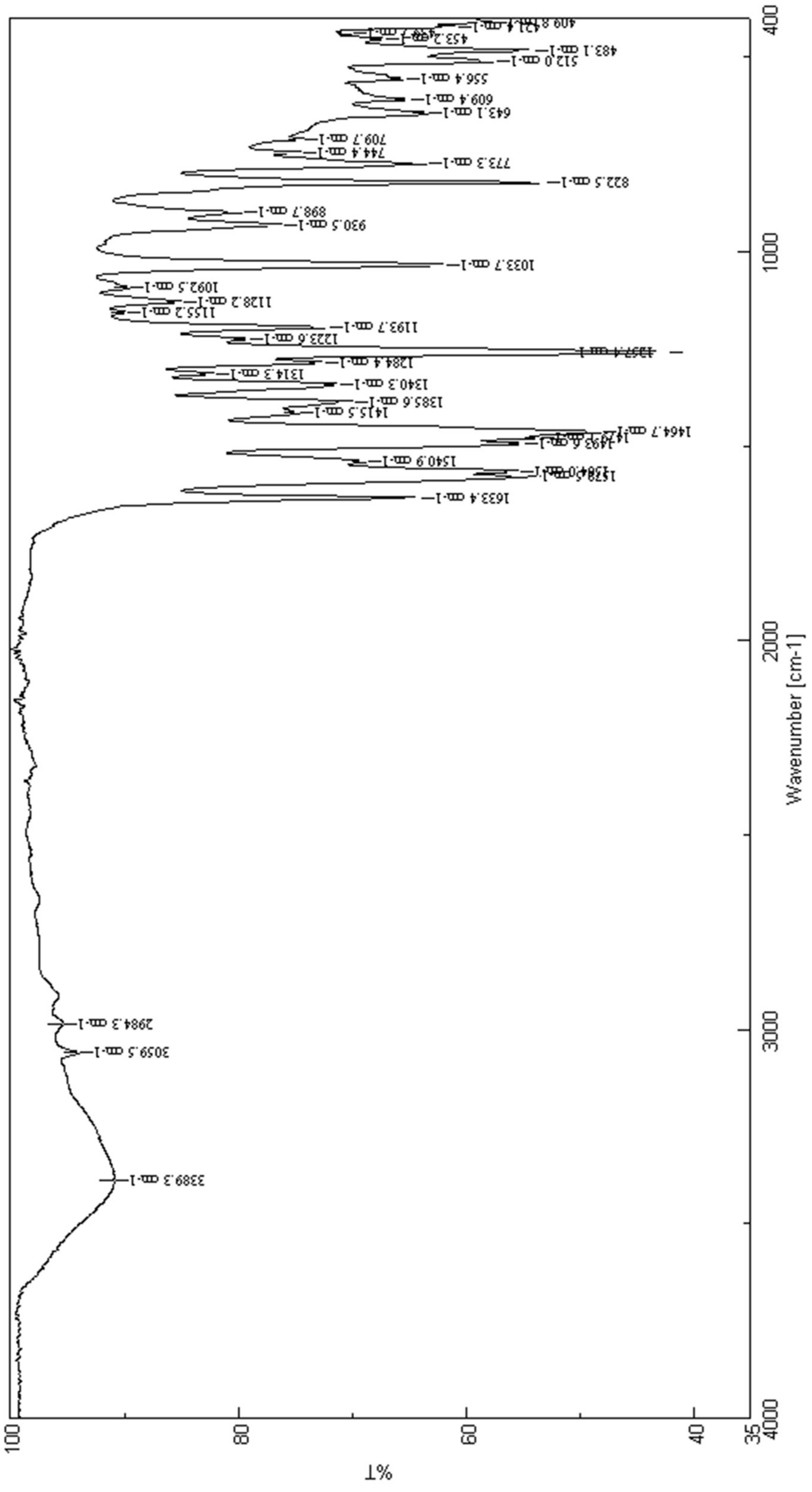

Figure S6. FT-IR spectrum of **Eu oxo.**

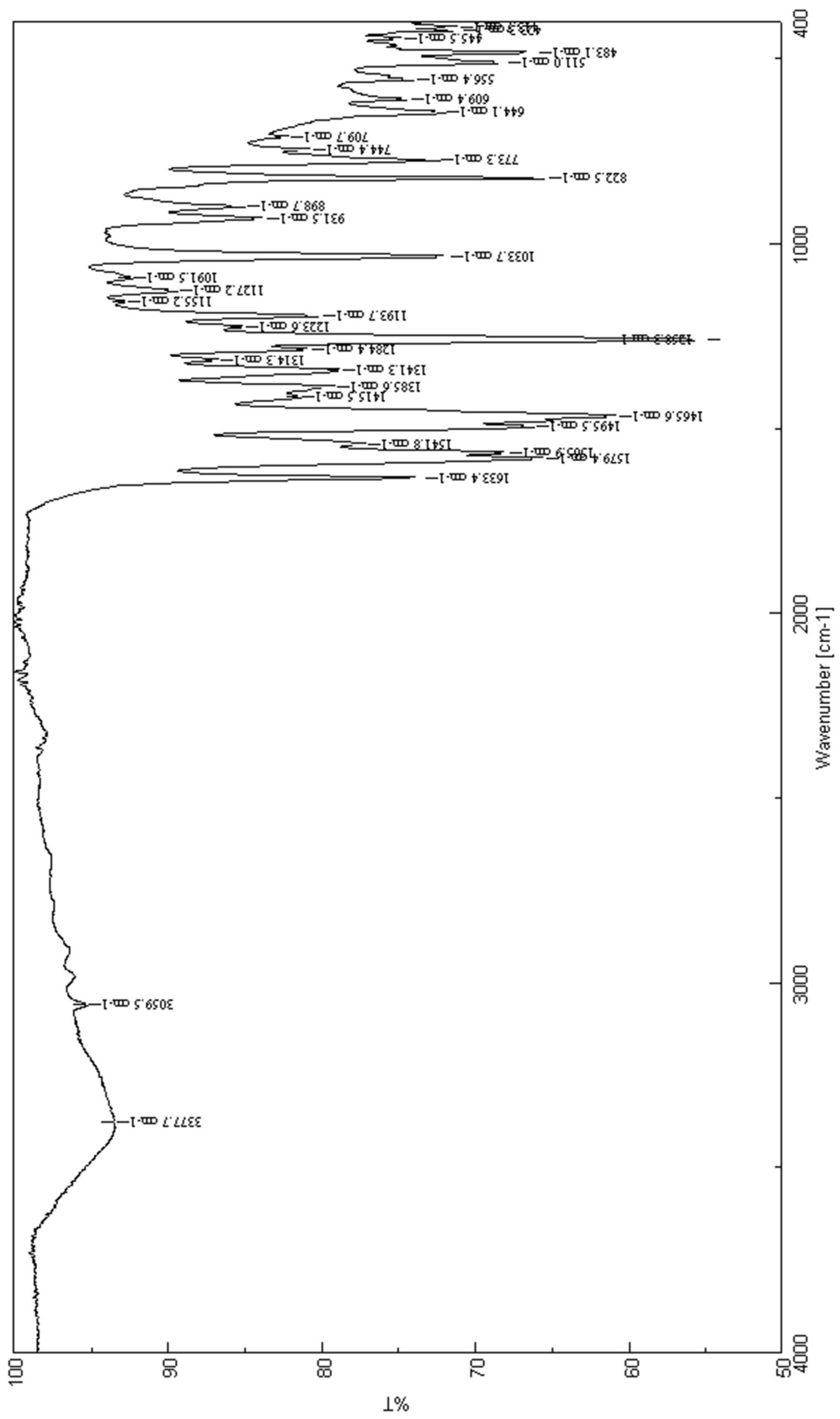

Figure S7. FT-IR spectrum of **Gd oxo.**

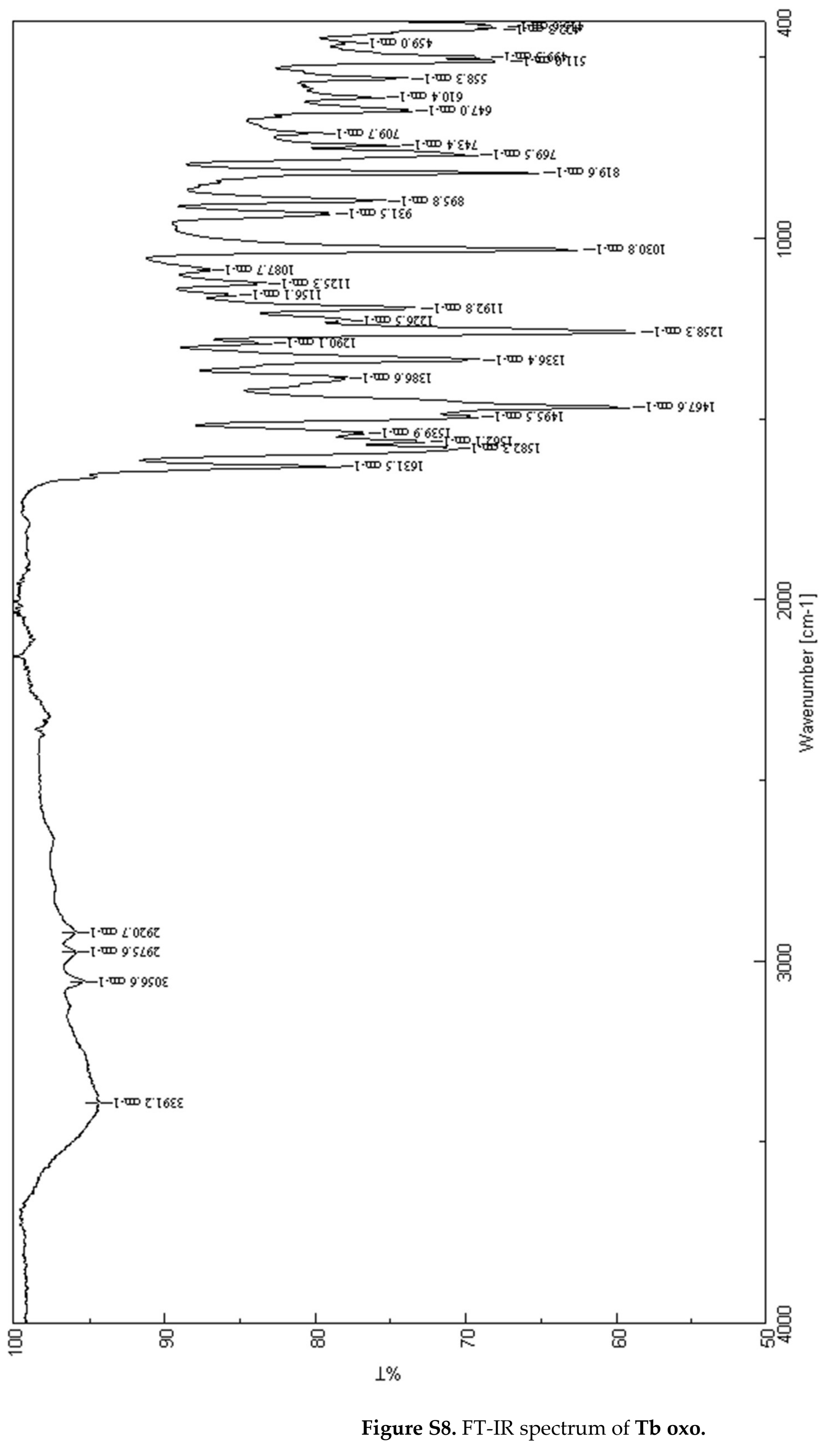

Figure S8. FT-IR spectrum of **Tb oxo.**

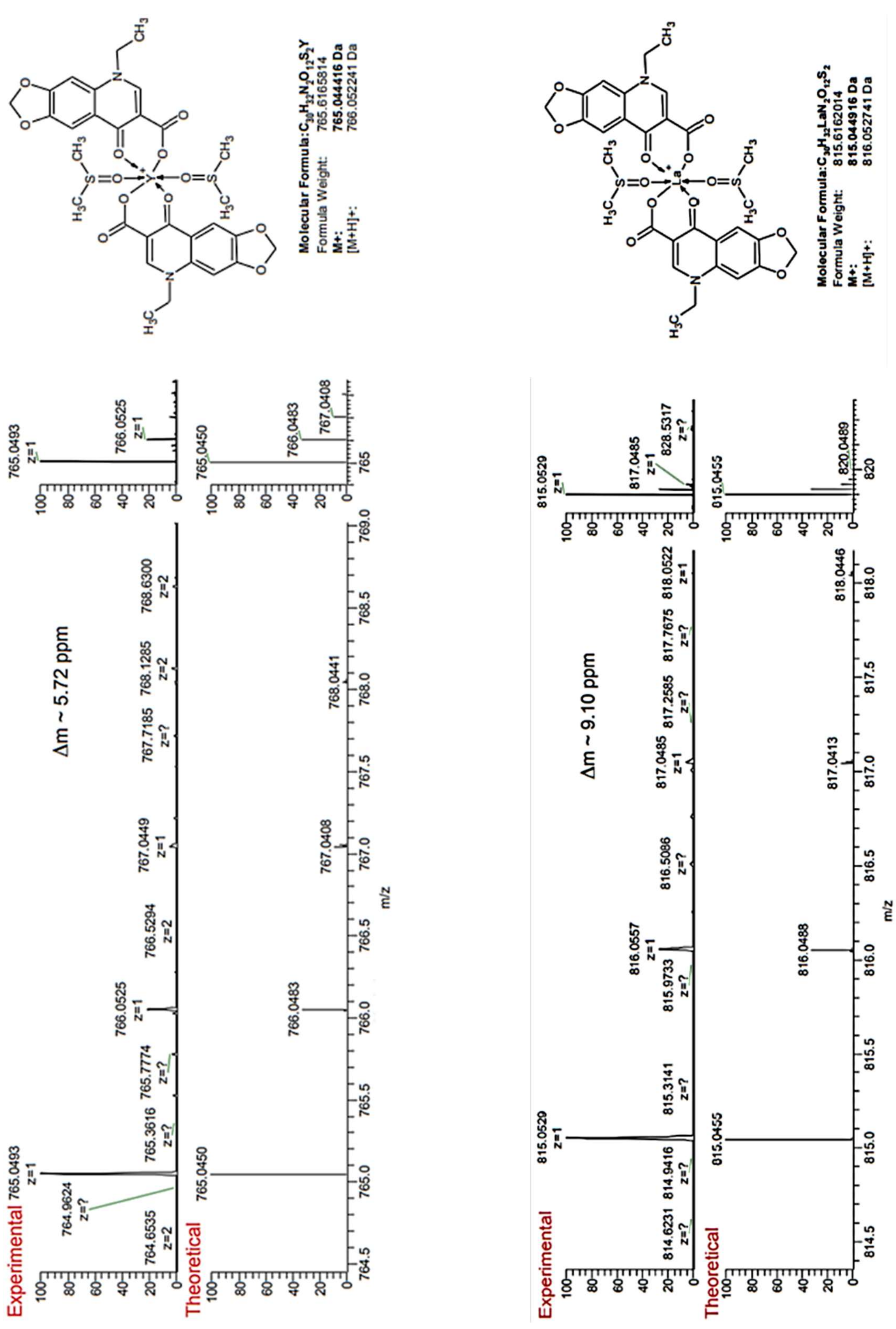

Figure S9. MS spectrum for **Y oxo** (left) and **La oxo (**right).

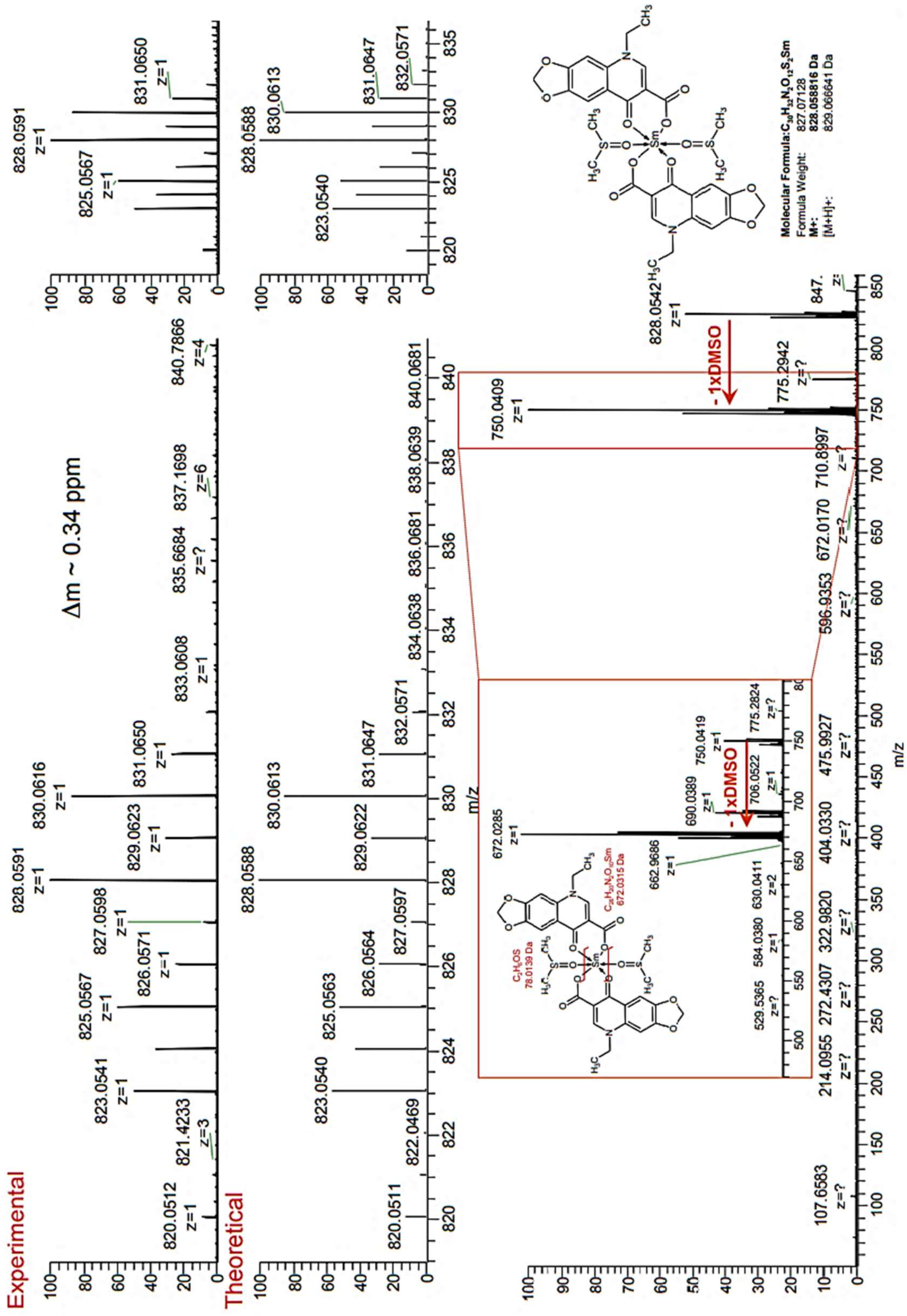

Figure S10. MS, MS/MS and MS/MS/MS spectra for **Sm oxo.**

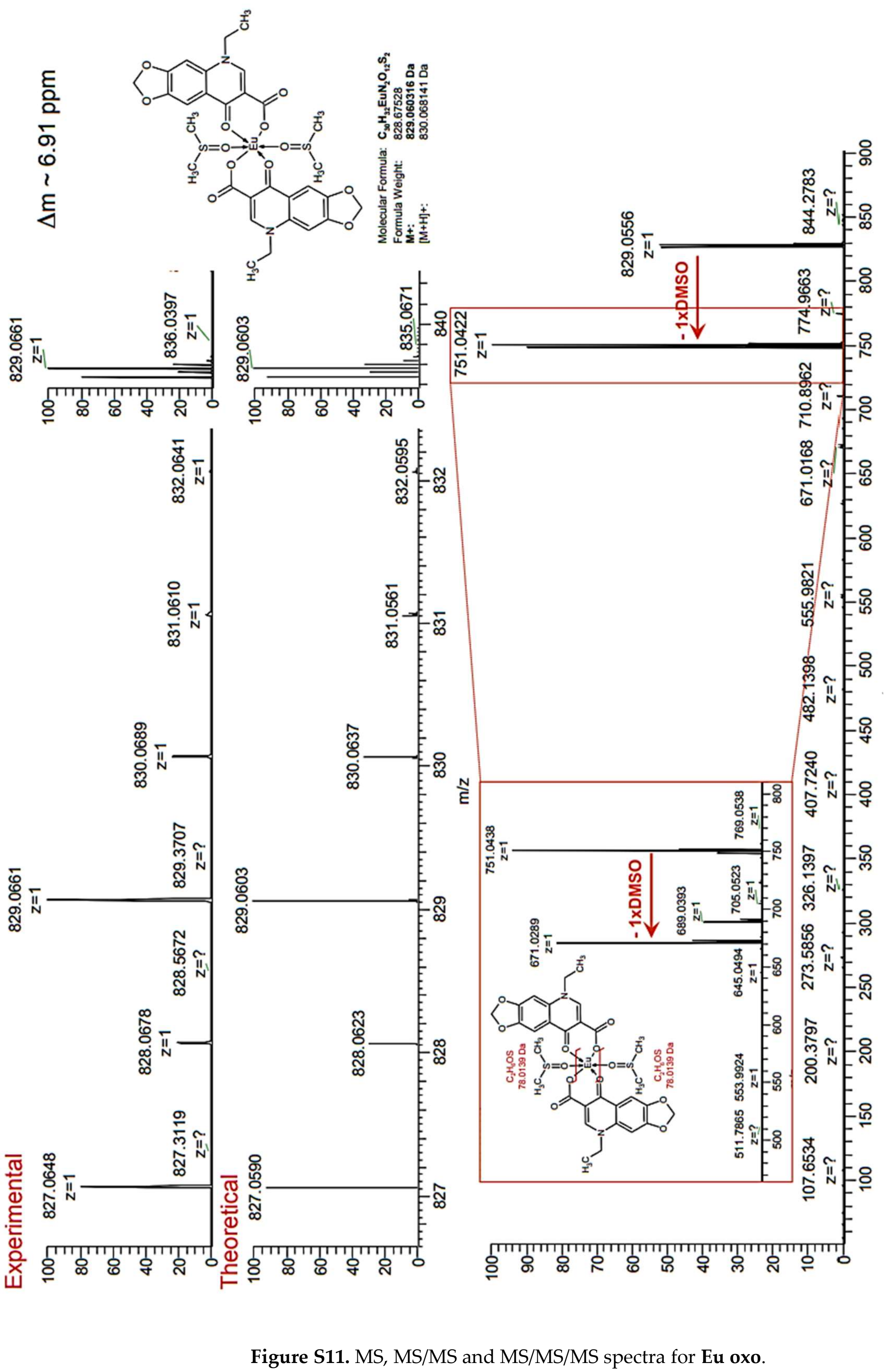

Figure S11. MS, MS/MS and MS/MS/MS spectra for **Eu oxo**.

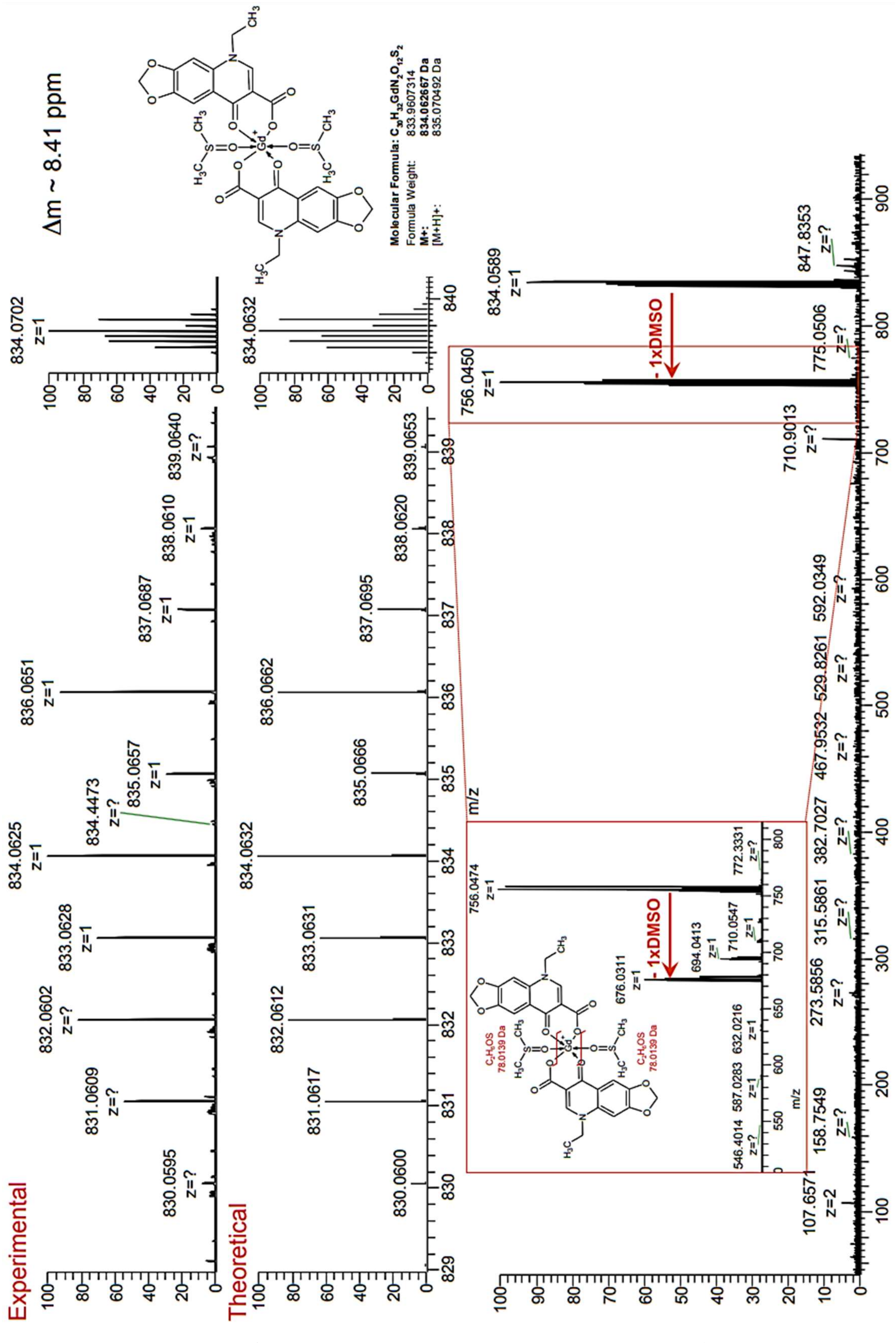

Figure S12. MS, MS/MS and MS/MS/MS spectra for **Gd oxo**.

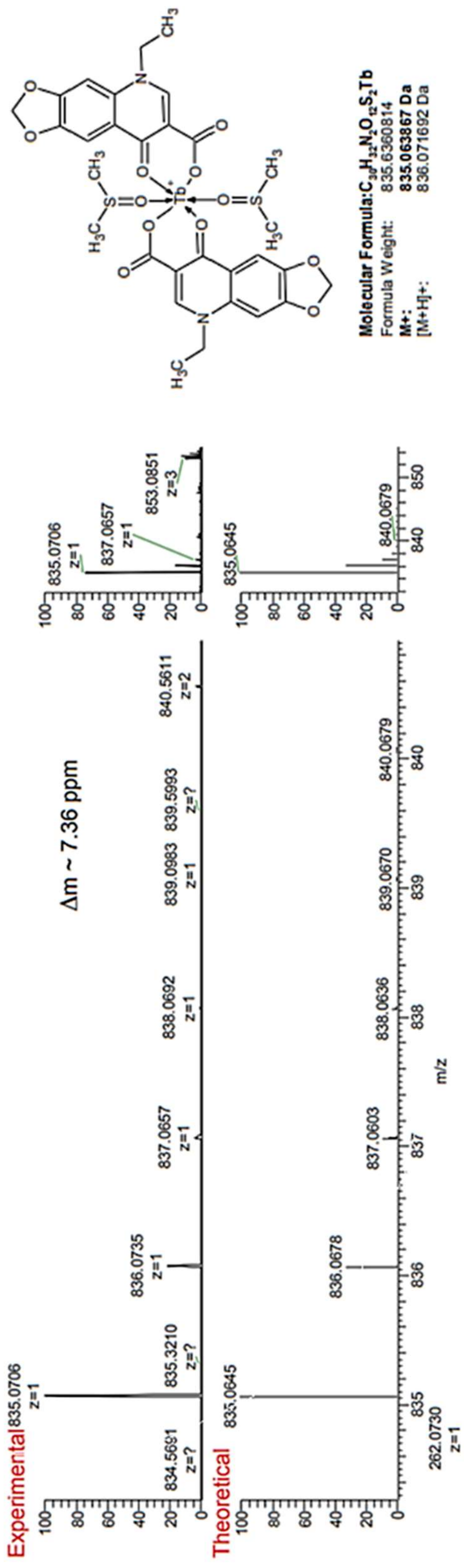

Figure S13. MS spectrum for **Tb oxo**.

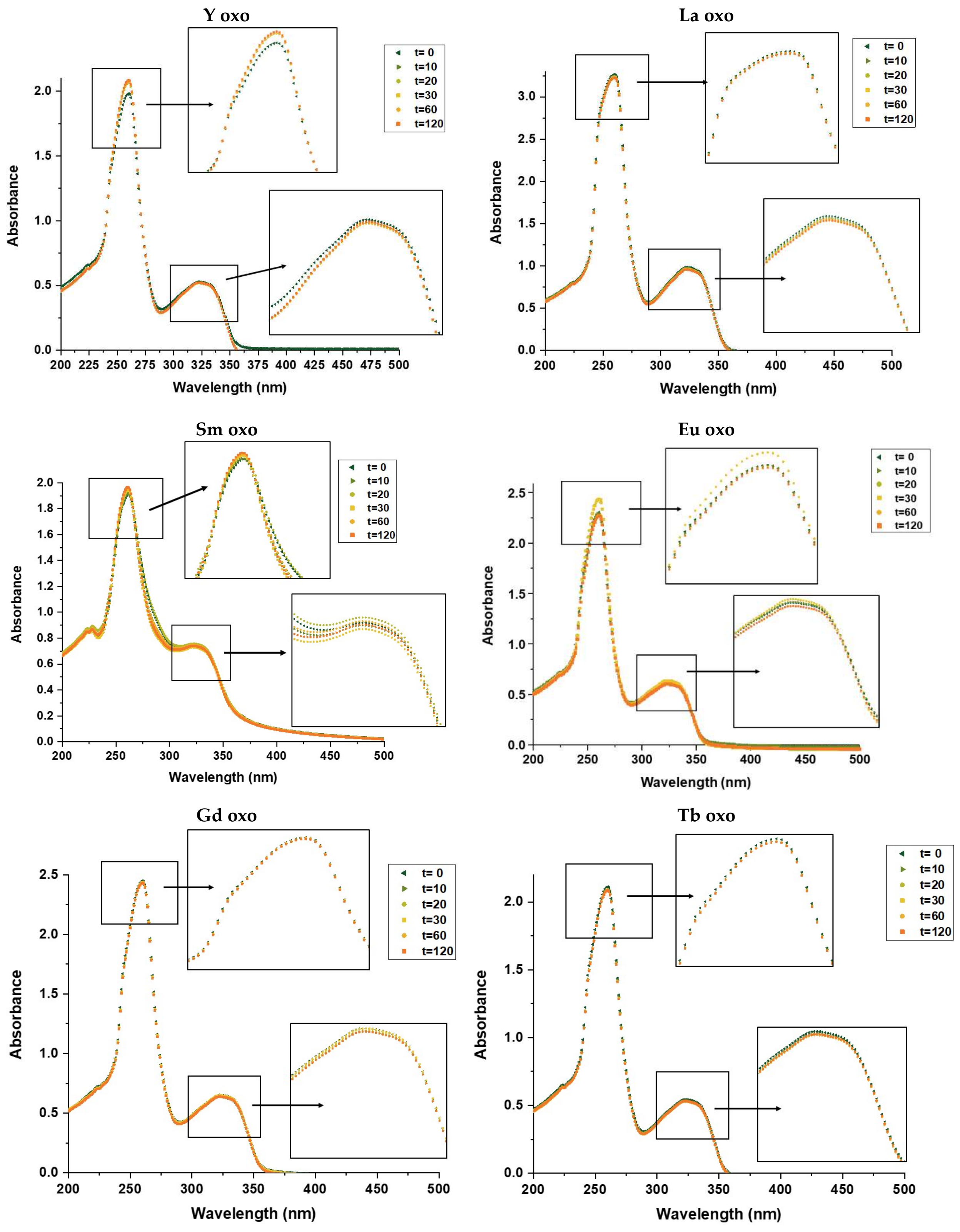

Figure S14. Stability assay-UV-vis spectra of the complexes in DMSO-Tris Cl buffer mixture.

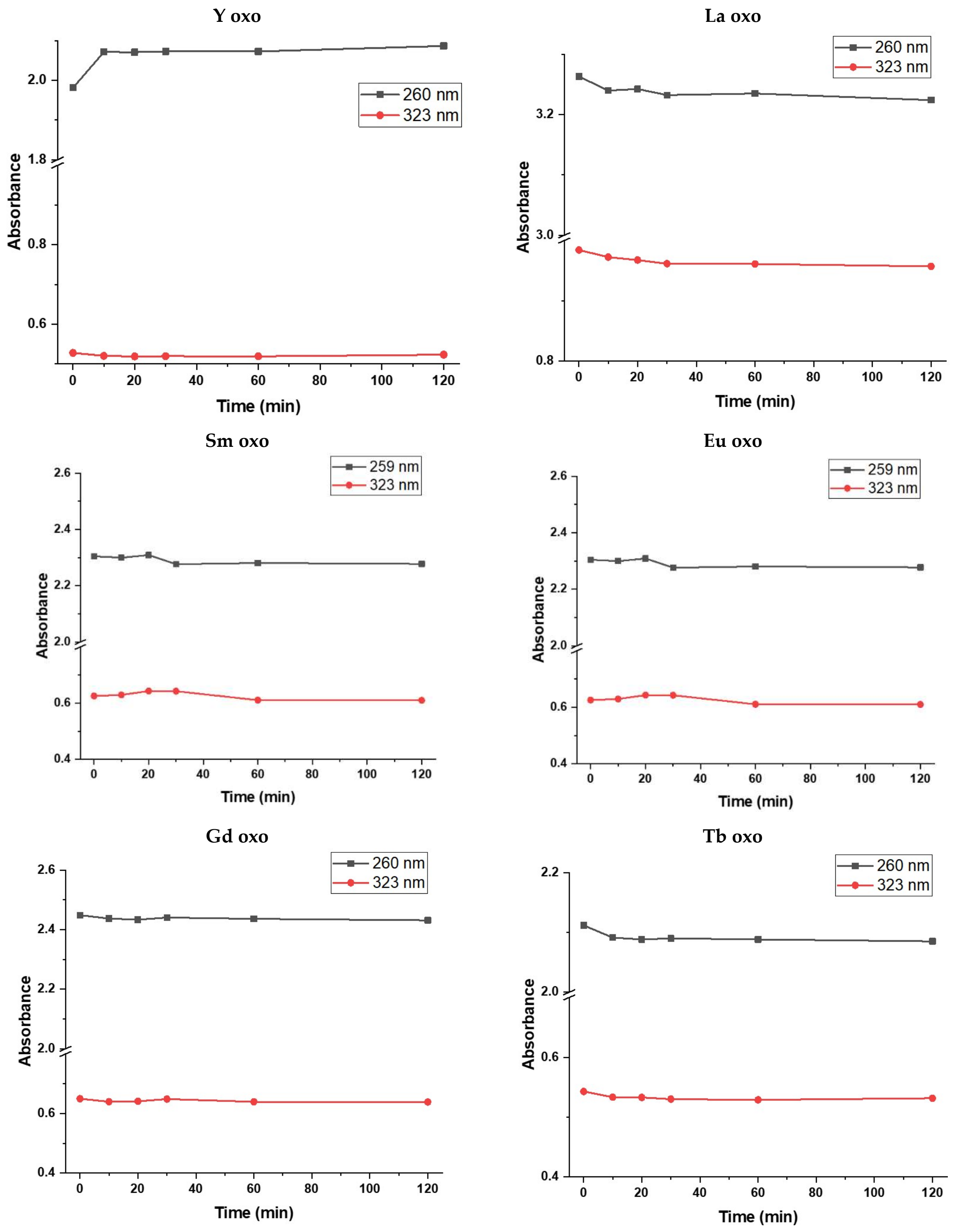

Figure S15. The variation of the absorbances of the studied complexes during the stability assay, in DMSO-Tris-HCl buffer mixture.

Table S2- Absorbances of tested compounds in UV-vis, in DMSO-TrisHCl buffer, [**compound**]= 40μM.

t (min)		0	10	20	30	60	120
Y oxo	**260**	1.9814	2.0715	2.0710	2.0729	2.0729	2.0867
	**232**	0.5276	0.5200	0.5187	0.5193	0.5190	0.5230
La oxo	**260**	3.2632	3.2398	3.2424	3.2324	3.2350	3.2240
	**323**	0.9843	0.9726	0.9677	0.9616	0.9612	0.9573
Sm oxo	**262**	1.9142	1.9169	1.9435	1.9368	1.9529	1.9629
	**322**	0.7445	0.7385	0.7566	0.7301	0.7482	0.7407
Eu oxo	**259**	2.3047	2.2995	2.4447	2.4430	2.2808	2.2775
	**323**	0.6255	0.6292	0.6431	0.6424	0.6105	0.6101
Gd oxo	**260**	2.4489	2.4373	2.4334	2.4409	2.4364	2.4316
	**323**	0.6497	0.6396	0.6411	0.6486	0.6394	0.6383
Tb oxo	**260**	2.1118	2.0912	2.0883	2.0900	2.0883	2.0850
	**323**	0.5428	0.5334	0.5329	0.5300	0.5288	0.5317

A.

B.

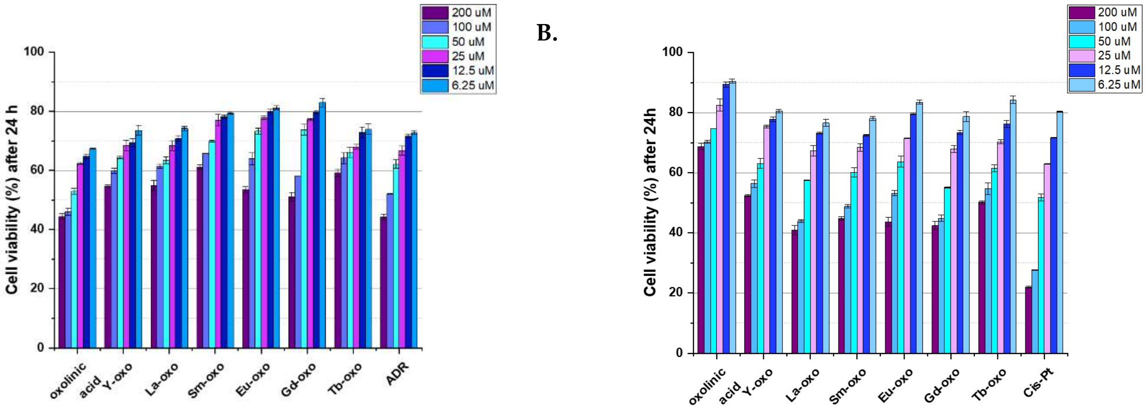

Figure S16. Cell viability (%) after 24h of incubation with tested compounds: **A.** on MDA-MB 231 cell line, **B.** LoVo cell line.

A. B.

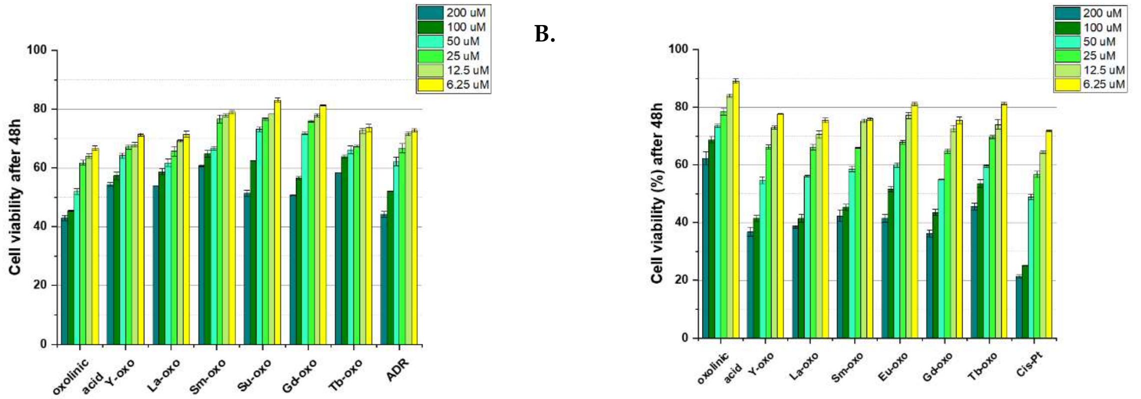

Figure S17. Cell viability (%) after 48h of incubation with tested compounds: **A**. on MDA-MB 231 cell line, **B**. LoVo cell line.

A. B.

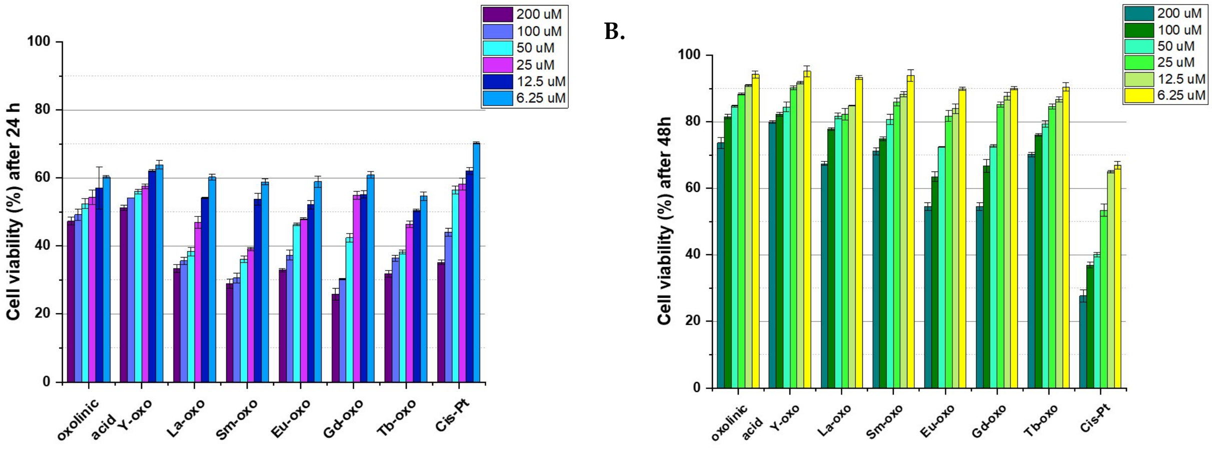

Figure S18. Normal cell viability (%) after **A**. 24h and **B**. 48h of incubation with tested compounds.

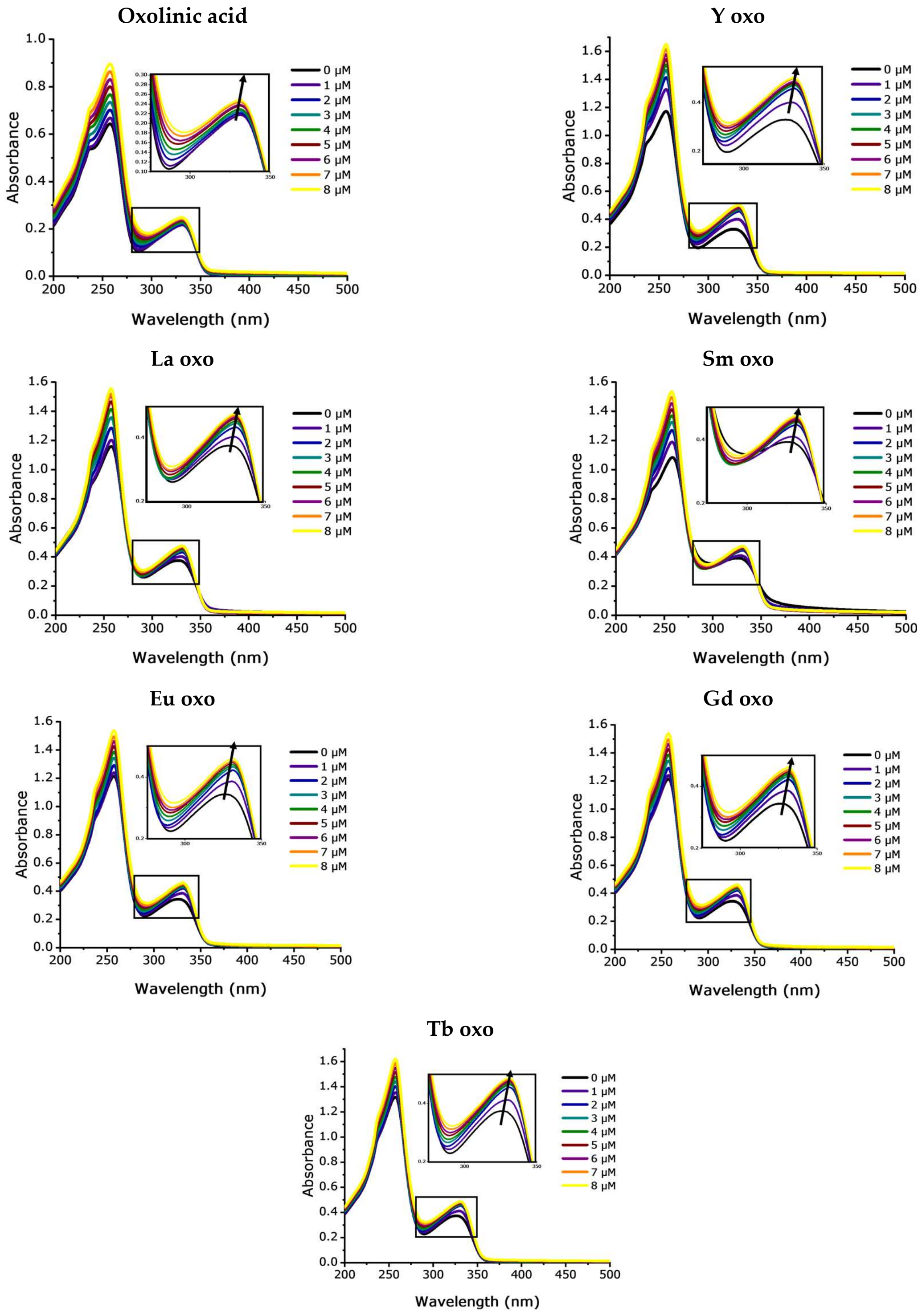

Figure S19. Absorption spectra of the tested compounds in the absence and presence of increasing amounts of DNA. [**compound**] = 20 μM; [DNA] = 0, 5, 10, 15, 20, 25, 30, 35, 40 μM. The arrows show the absorption changes upon increasing the DNA concentration.

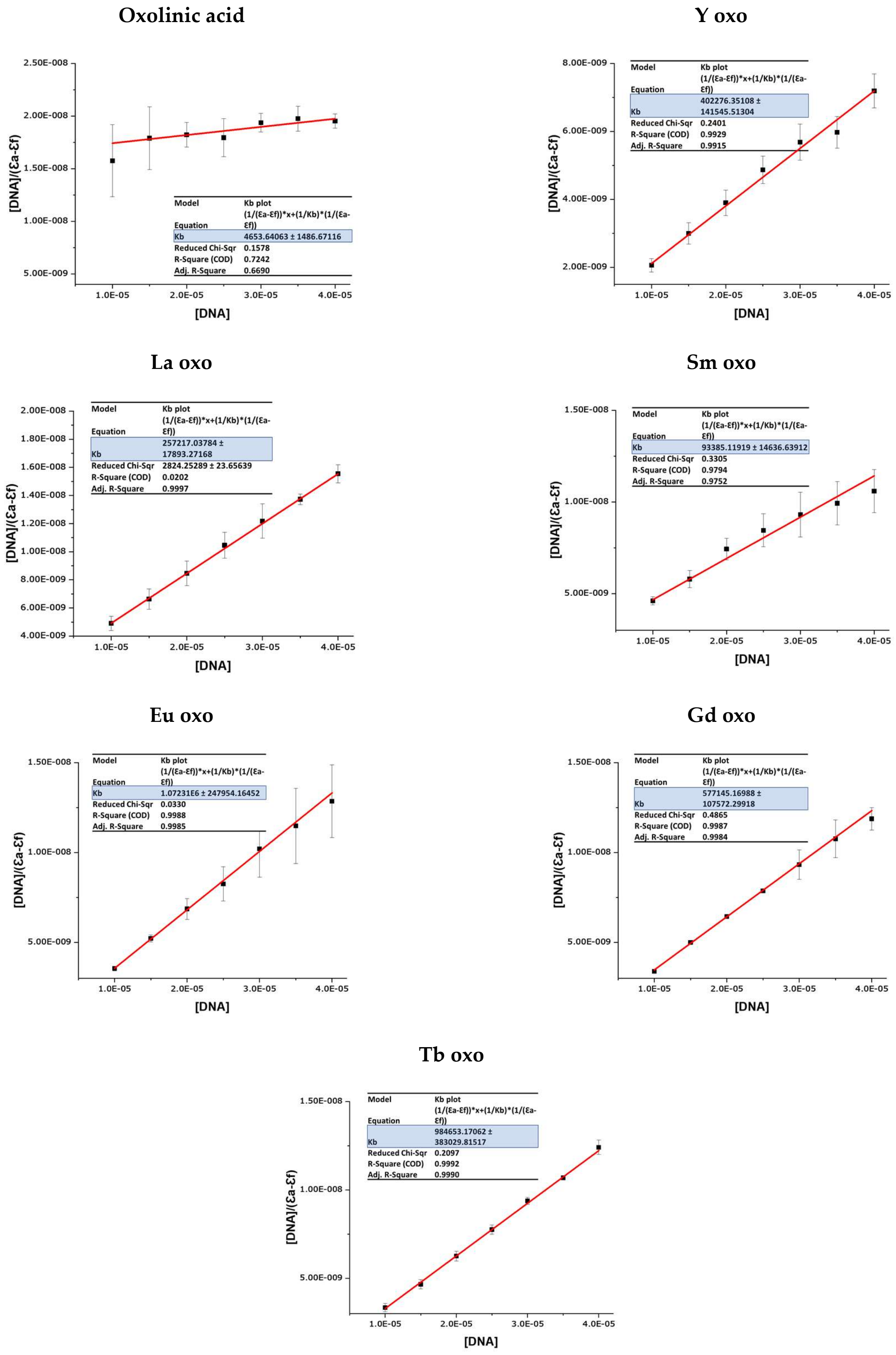

Figure S20. Representation of the DNA-binding constant (K_b) of the studied compounds.

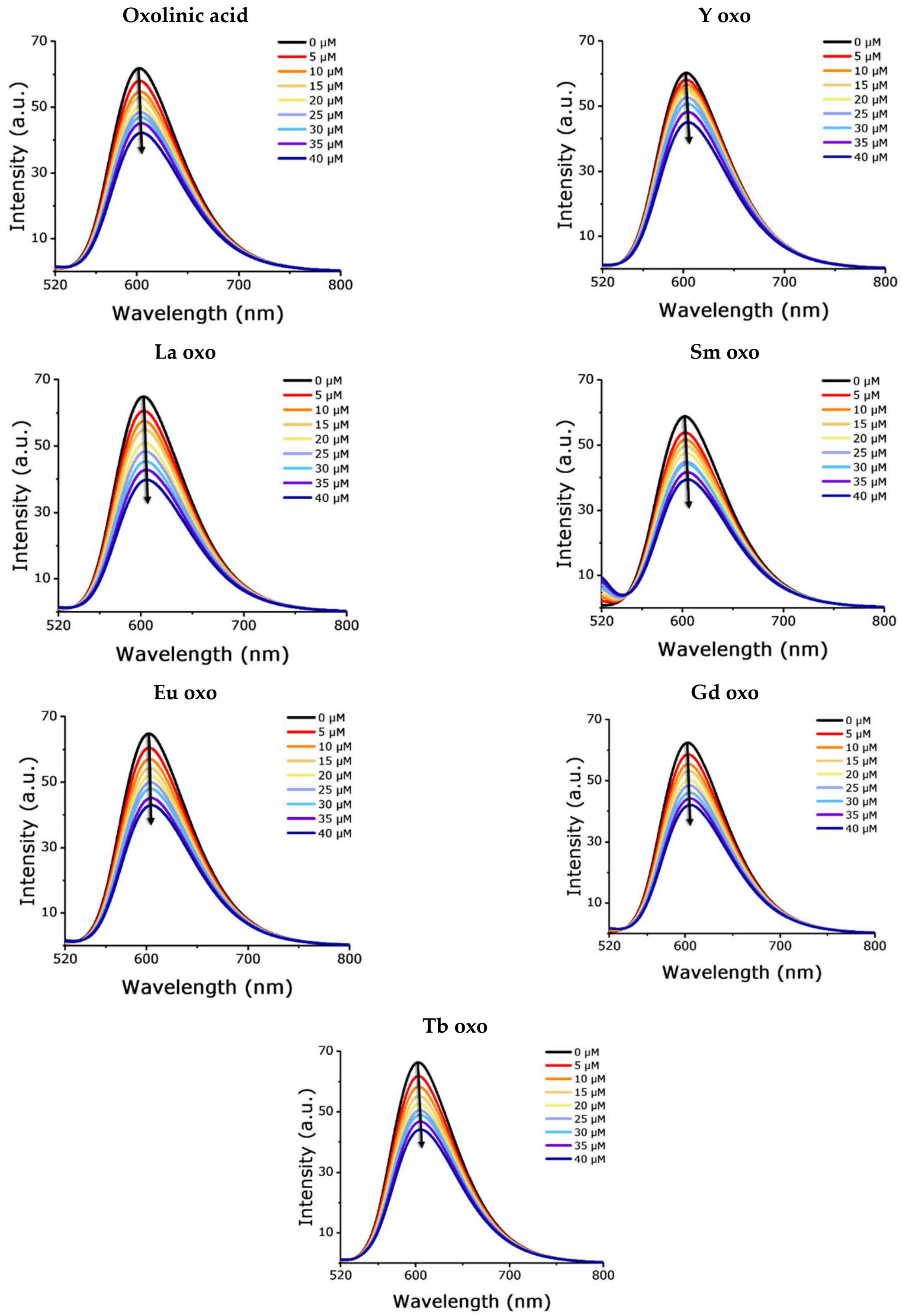

Figure S21. Fluorescence spectra of the EB - DNA system in the absence and presence of increasing amounts of the tested compounds. λ_{ex} = 500 nm, [EB] = 2 μM, [DNA] = 10 μM, [compound] = 0, 5, 10, 15, 20, 25, 30, 35, 40 μM. Arrows indicate the changes in fluorescence intensities upon increasing the concentrations of the tested compounds.

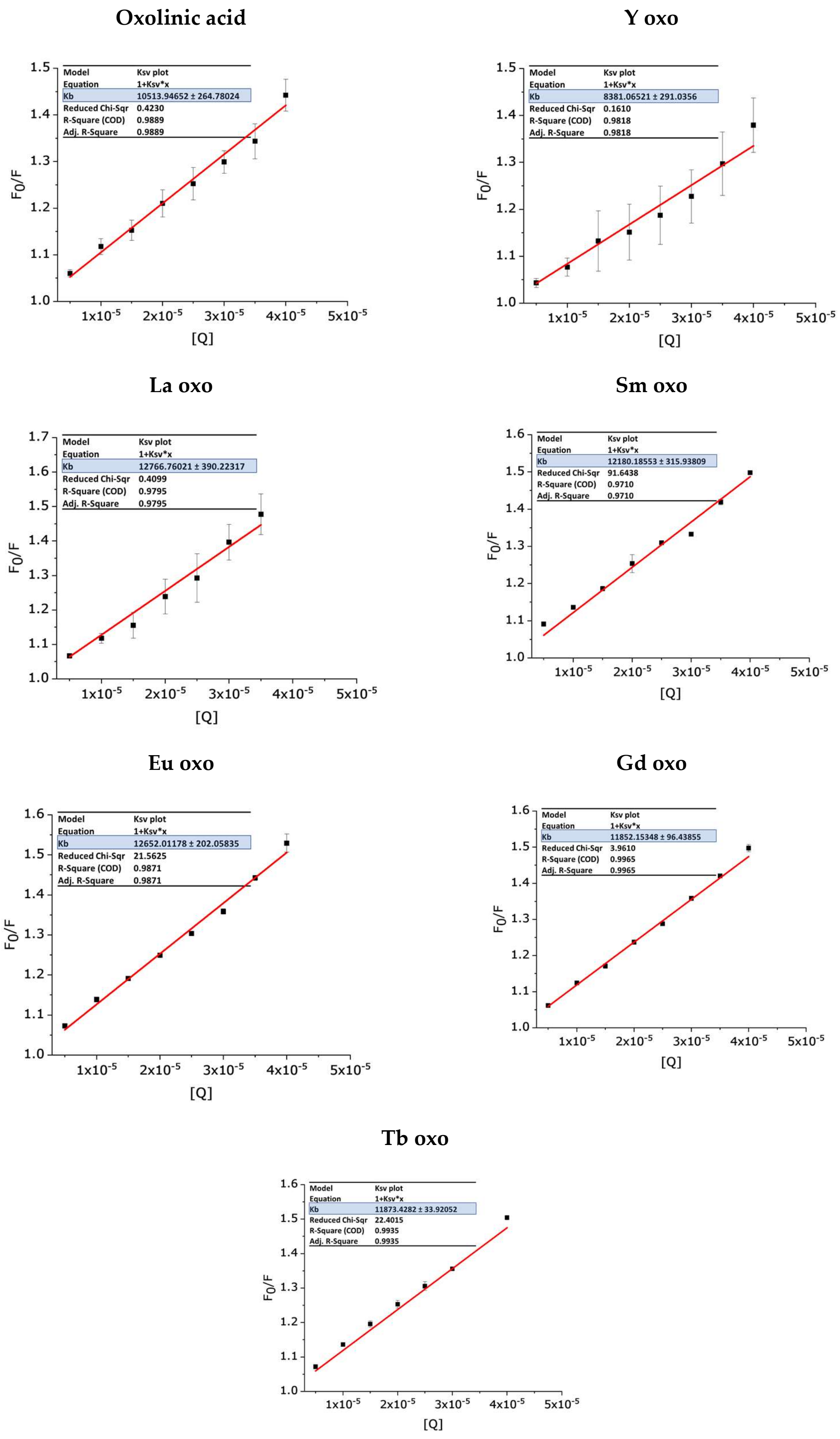

Figure S22. Graphical representation of the K_{SV} constants of the studied compounds.

Oxolinic acid

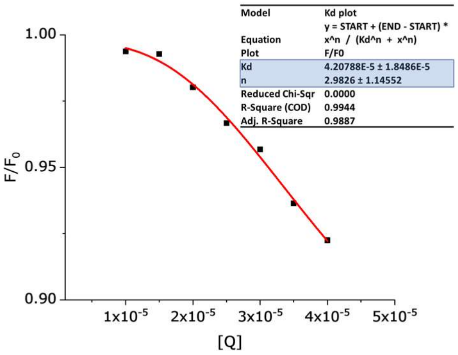

Y oxo

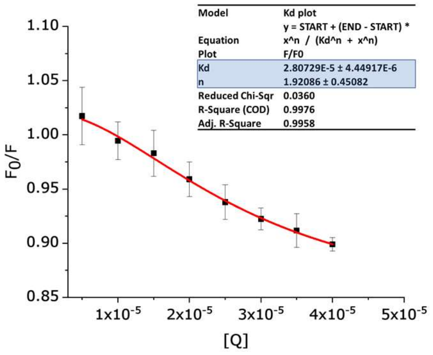

La oxo

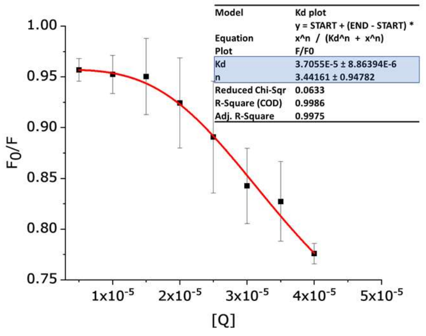

Sm oxo

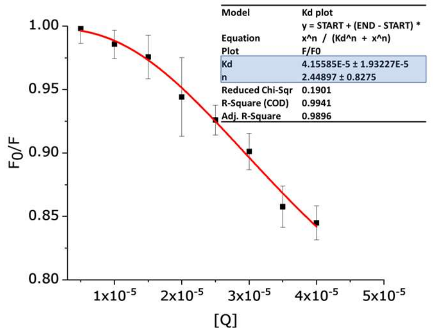

Eu oxo

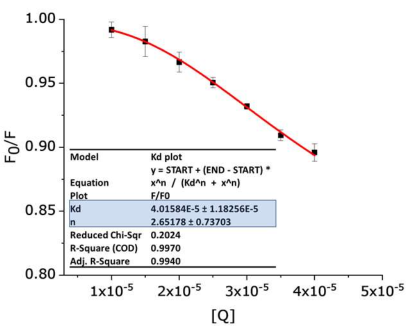

Gd oxo

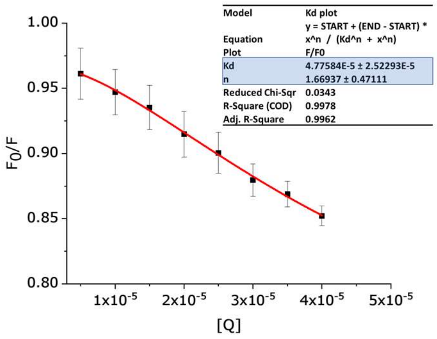

Tb oxo

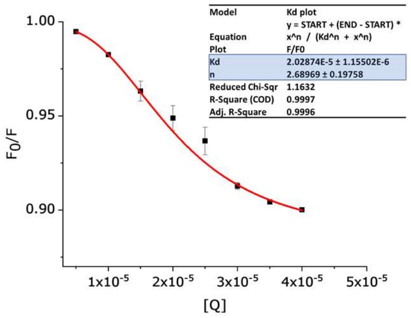

Figure S23. Representation of the K_{50} constant of the studied compounds.

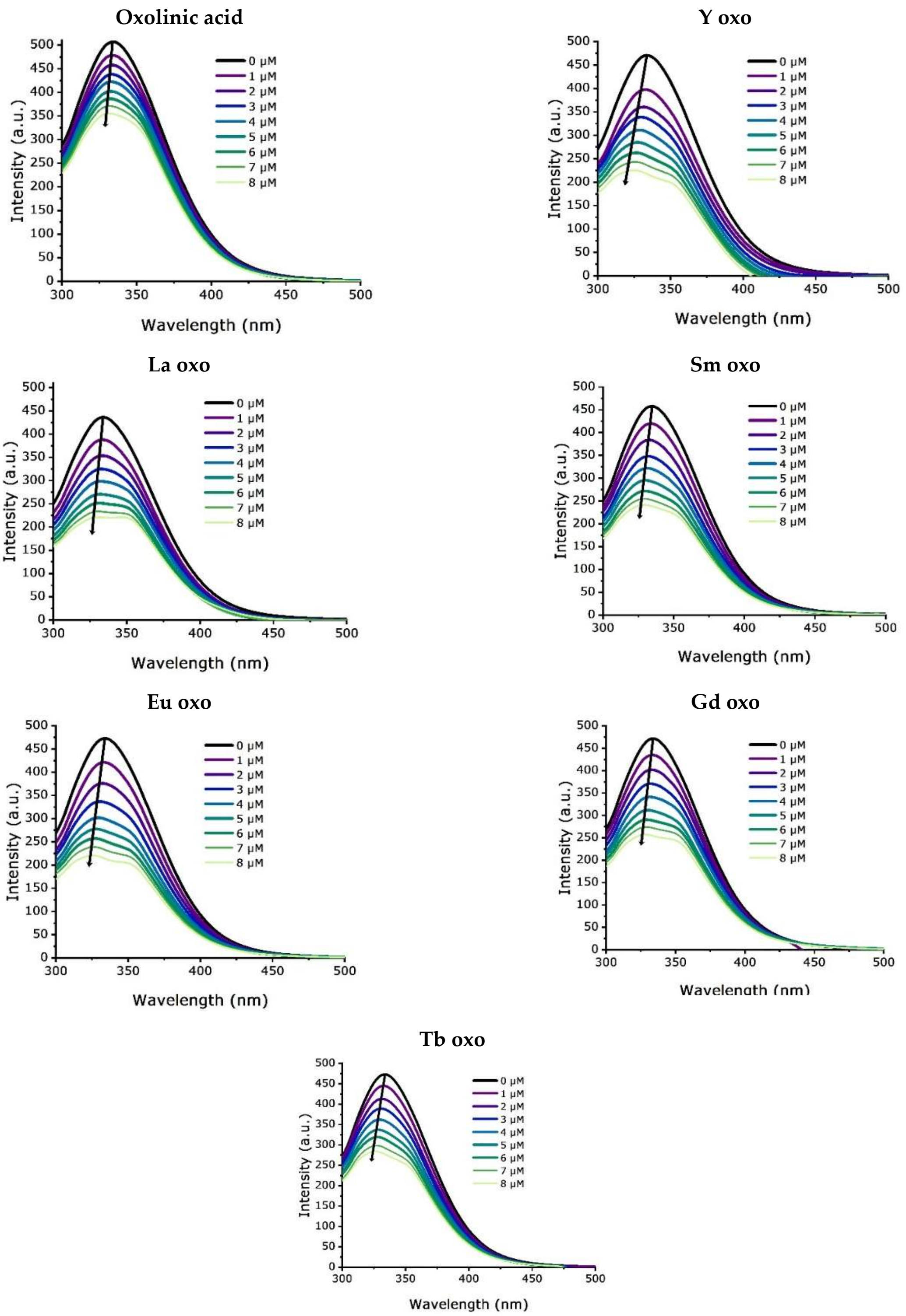

Figure S24. Changes in fluorescence intensity of free HSA *vs.* compound-HSA systems; the black arrows indicate a decrease of the intensity upon the addition of increasing amounts of compound; [**HSA**] = 2.5 μM, [**compound**] = 0, 1, 2, 3, 4, 5, 6, 7, 8 μM.

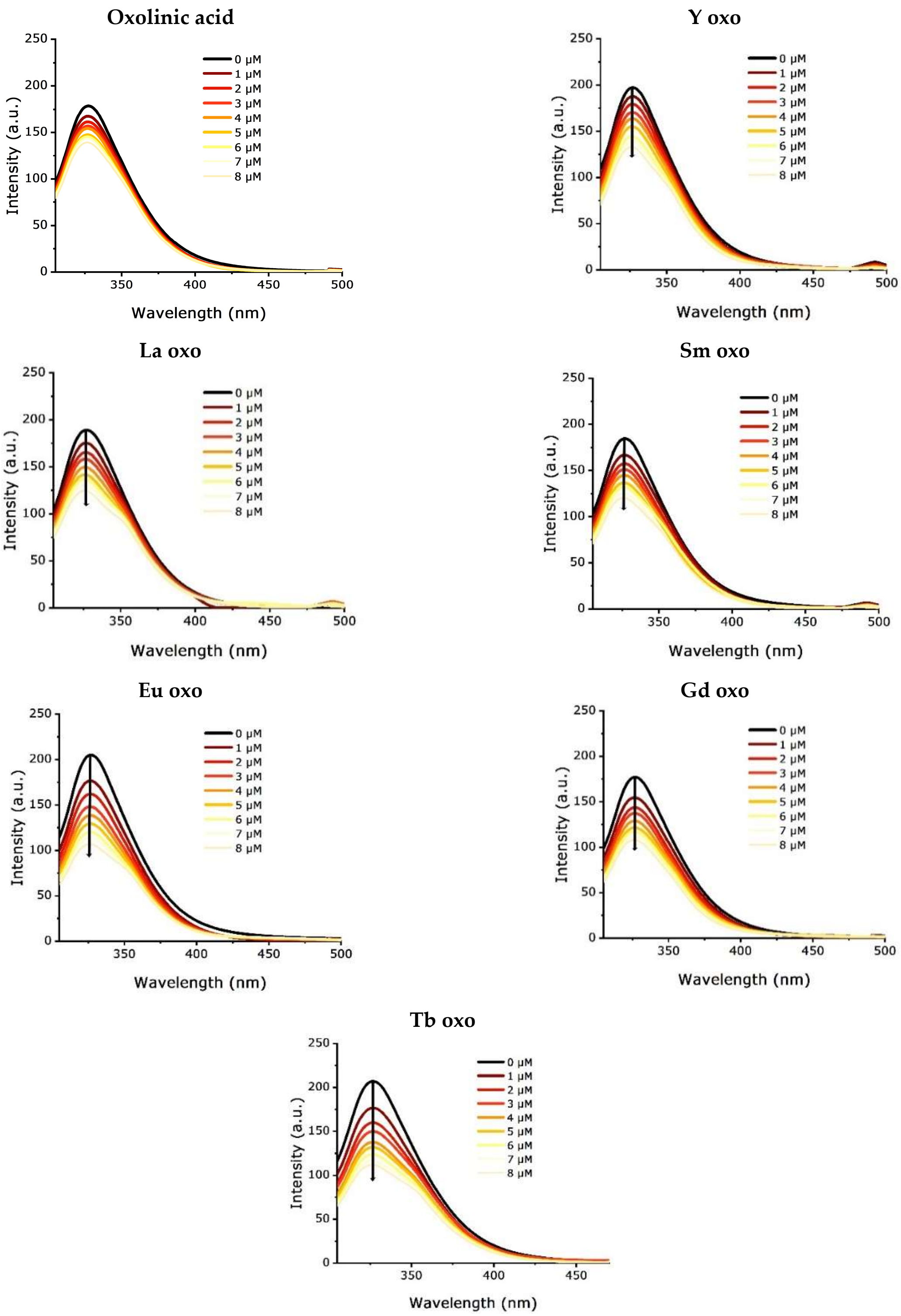

Figure S25. Changes in fluorescence intensity of free apo Tf *vs.* compound-apo Tf systems; the black arrows indicate a decrease of the intensity upon the addition of increasing amounts of compound. **[apo-Tf]** = 1 μM, [**compound**] = 0, 1, 2, 3, 4, 5, 6, 7, 8 μM.

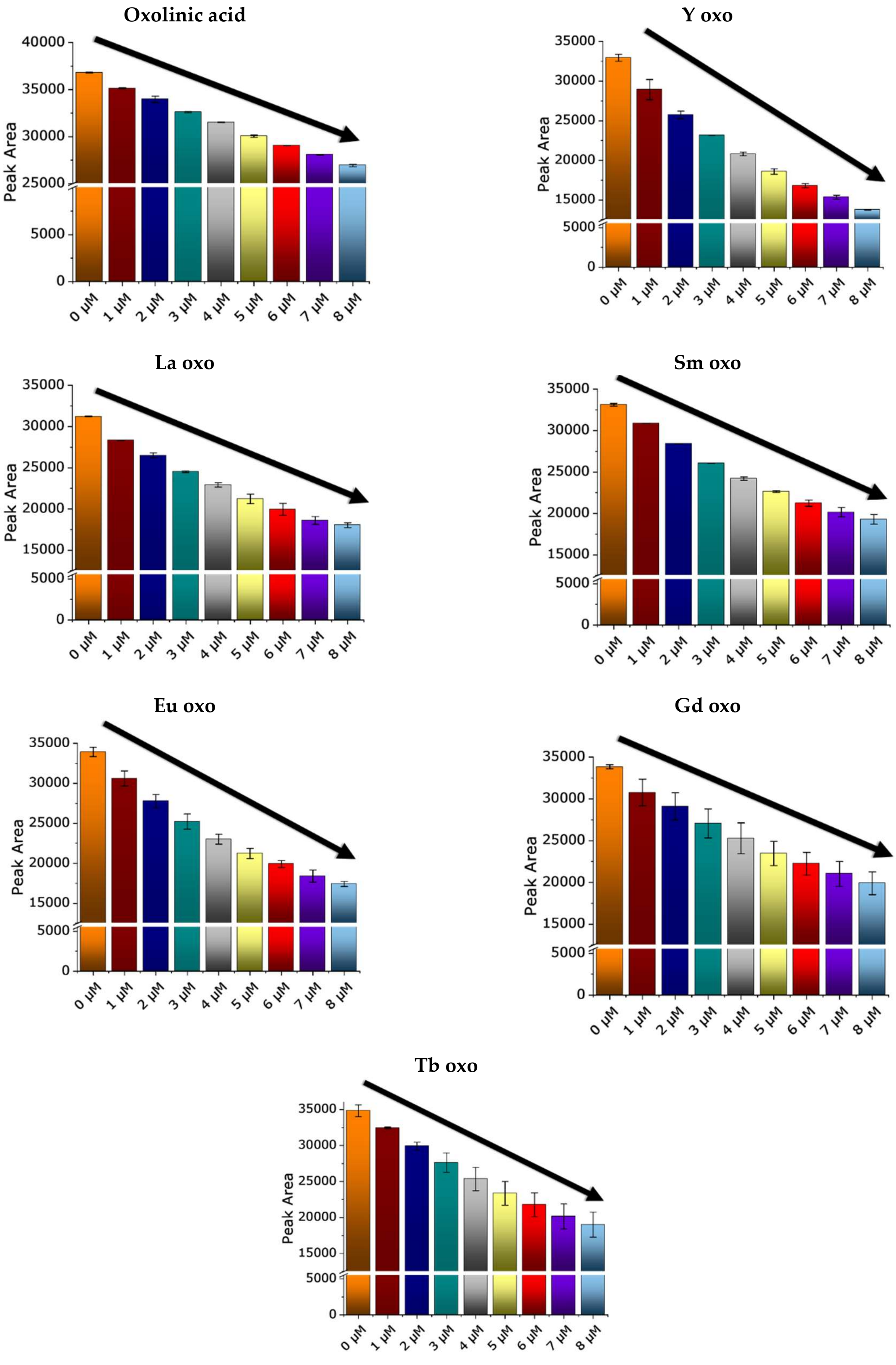

Figure S26. Changes in the peak areas for the compound-HSA systems. [**HSA**] = 2.5 µM, [**compounds**] = 0, 1, 2, 3, 4, 5, 6, 7, 8 µM.

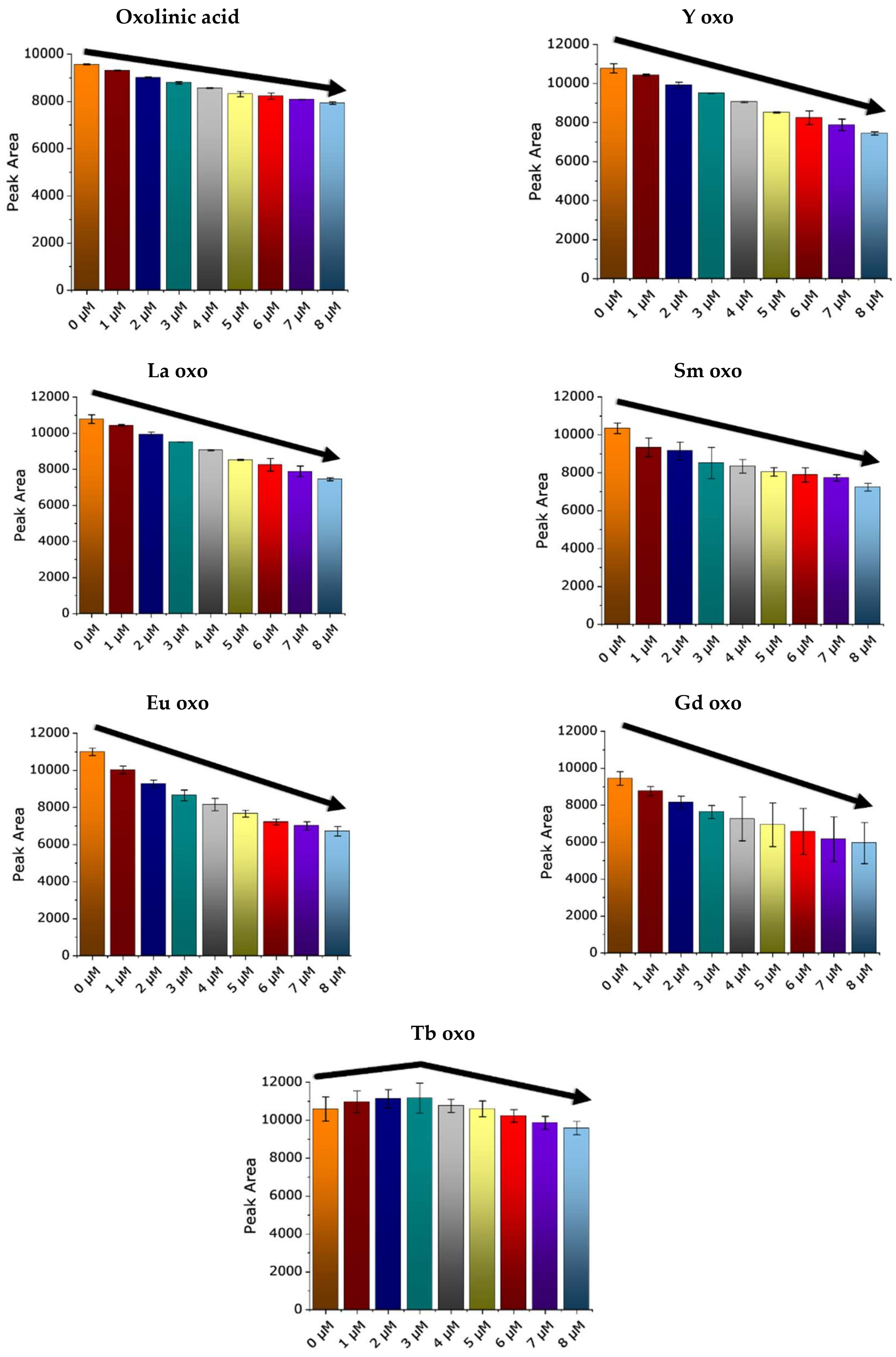

Figure S27. Variation of the apo-Tf fluorescence peak area upon adding increasing amounts of studied compounds. [**apo-Tf**] = 1 μM, [**compound**] = 0, 1, 2, 3, 4, 5, 6, 7, 8 μM.

Oxolinic acid

Classical Stern-Volmer plot	Modified Stern-Volmer plot

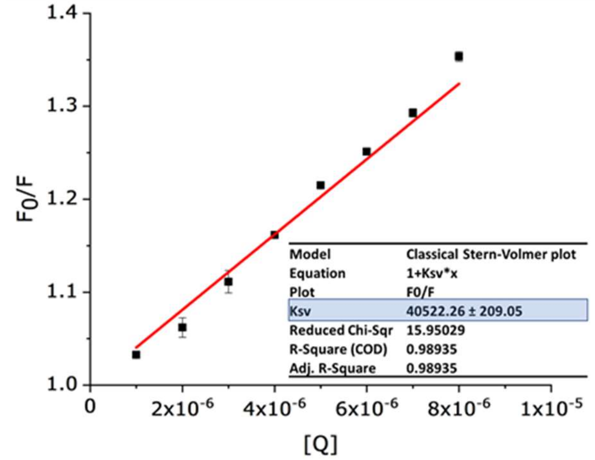

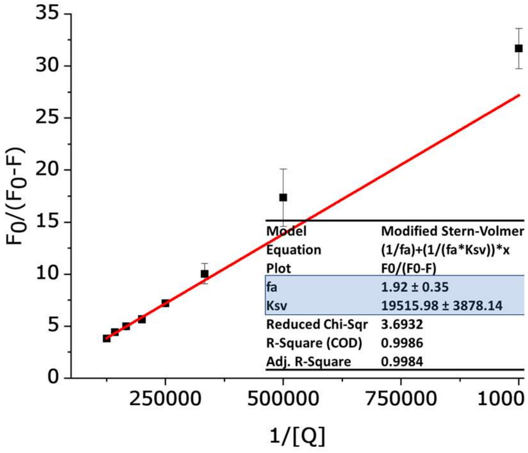

Y oxo

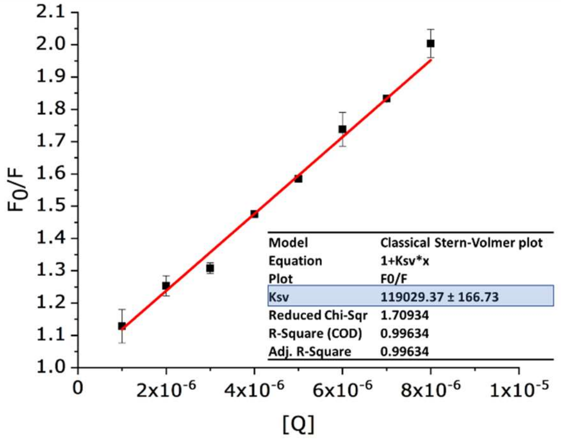

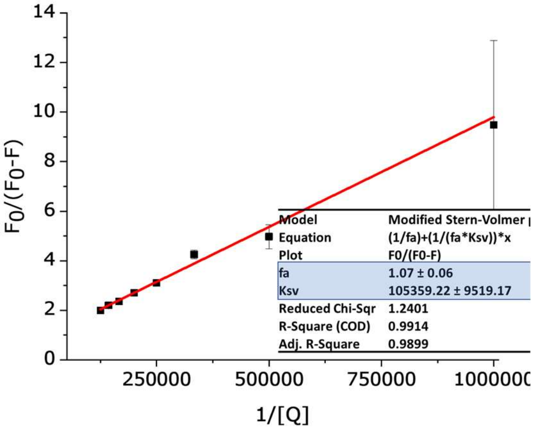

La oxo

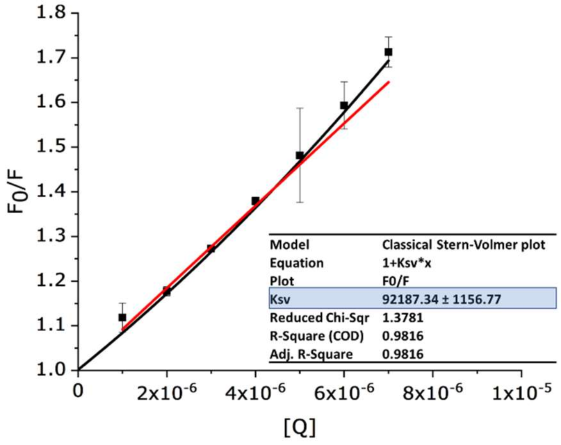

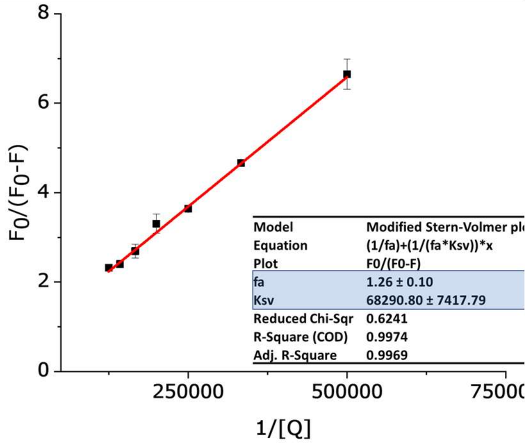

Sm oxo

Classical Stern-Volmer plot	Modified Stern-Volmer plot

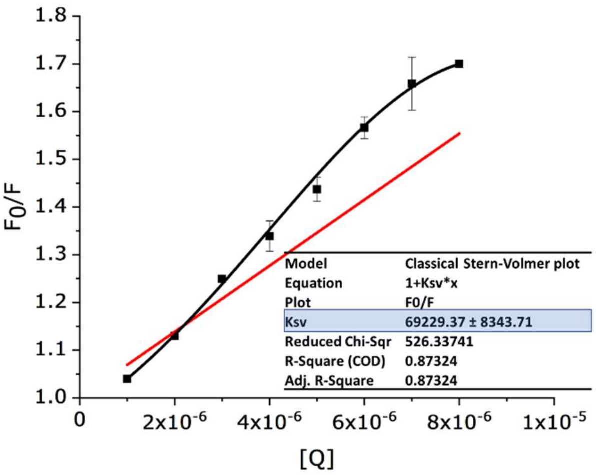

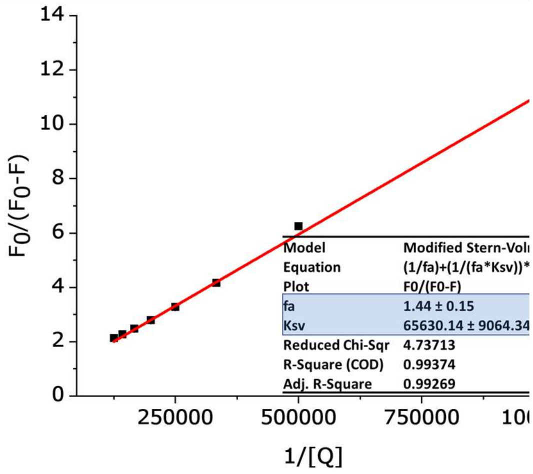

Eu oxo

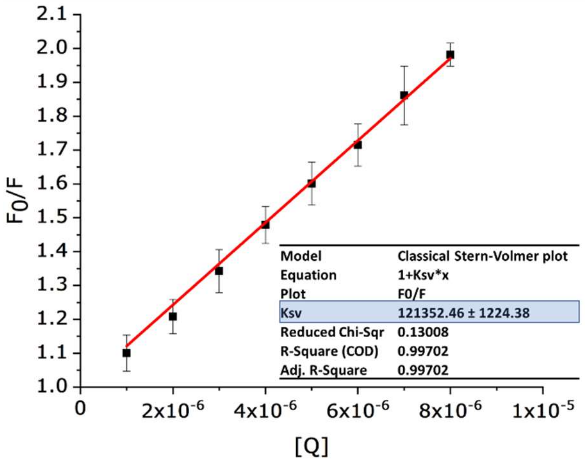

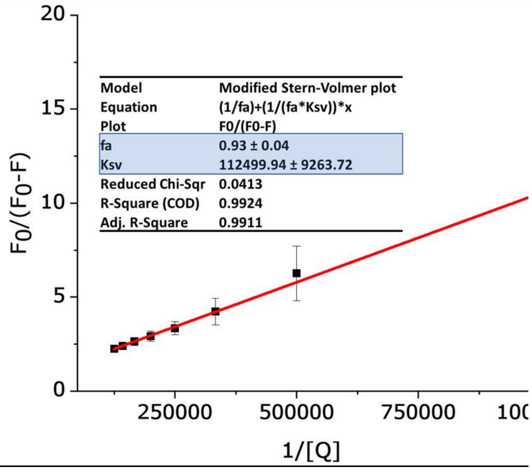

Gd oxo

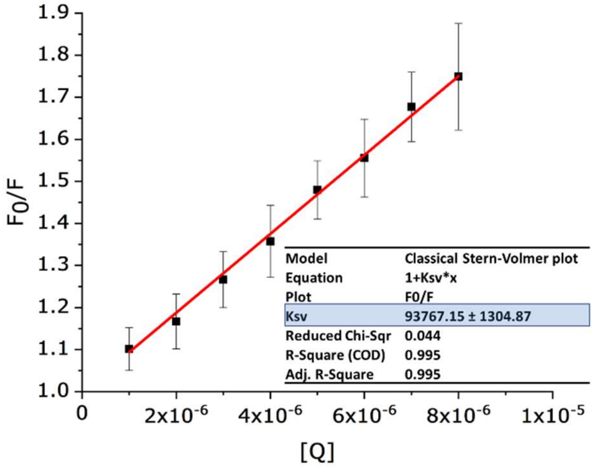

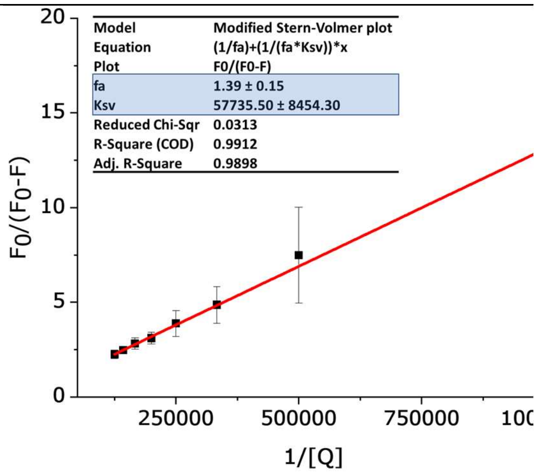

Tb oxo

Classical Stern-Volmer plot	Modified Stern-Volmer plot

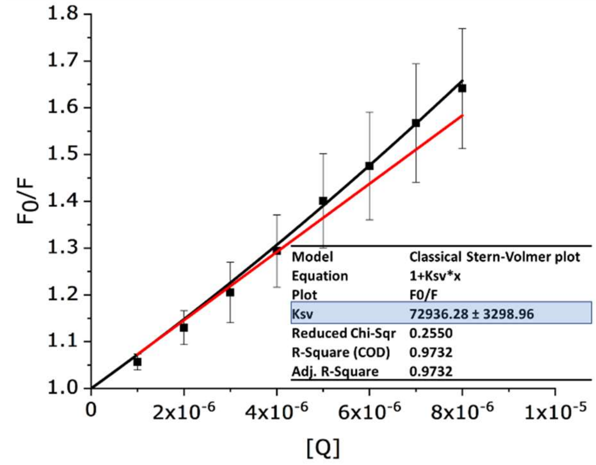

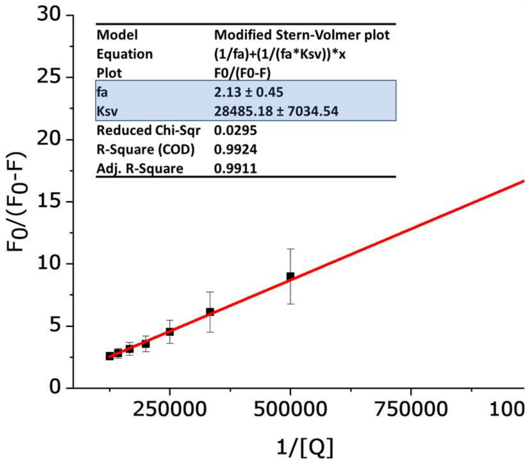

Figure S28. Classical (left) and modified (right) Stern-Volmer plots for each of the HSA interaction studies.

Oxolinic acid

Classical Stern-Volmer plot	Modified Stern-Volmer plot

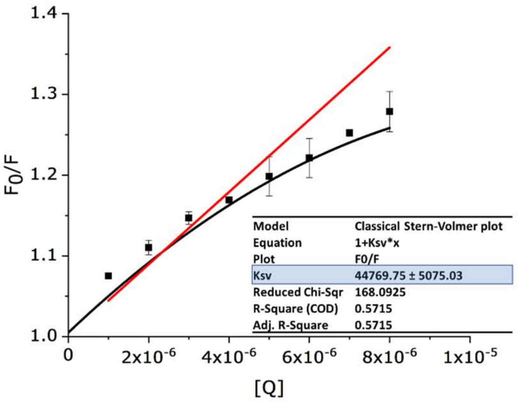

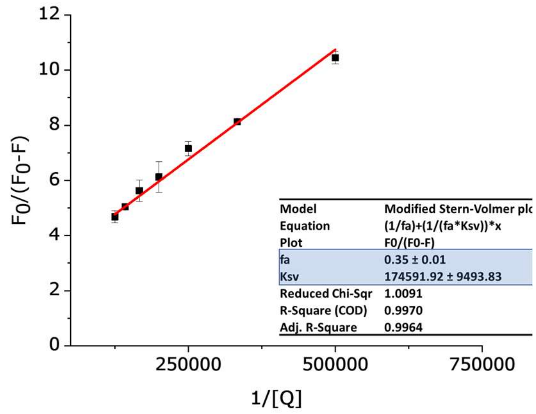

Y oxo

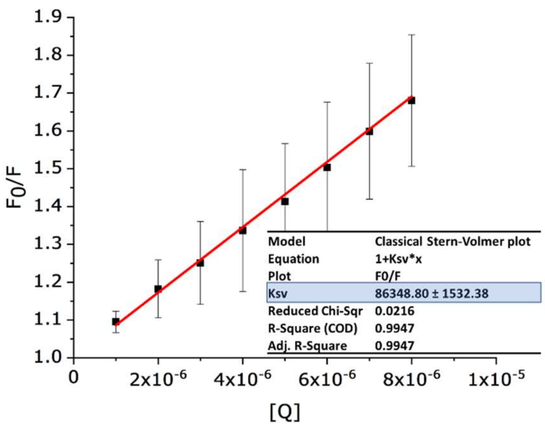

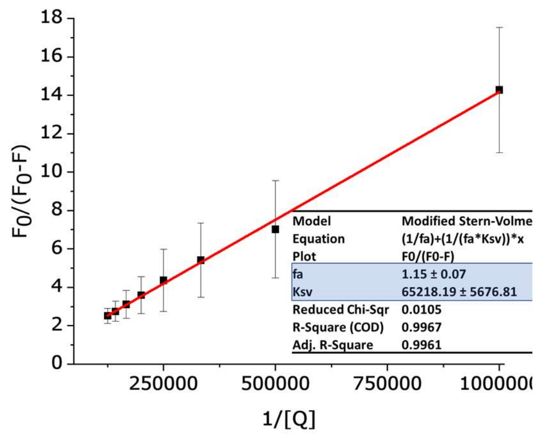

La oxo

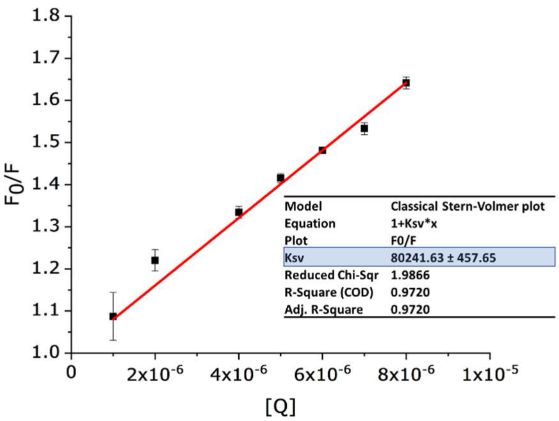

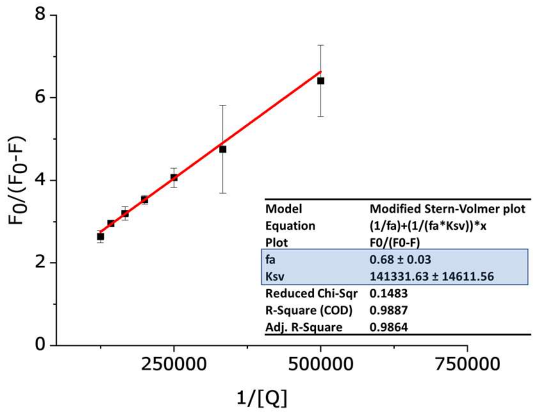

Sm oxo

Classical Stern-Volmer plot | **Modified Stern-Volmer plot**

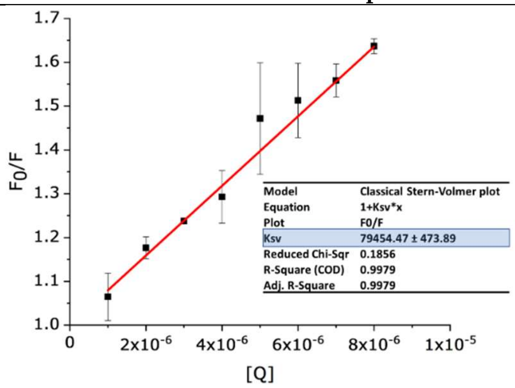

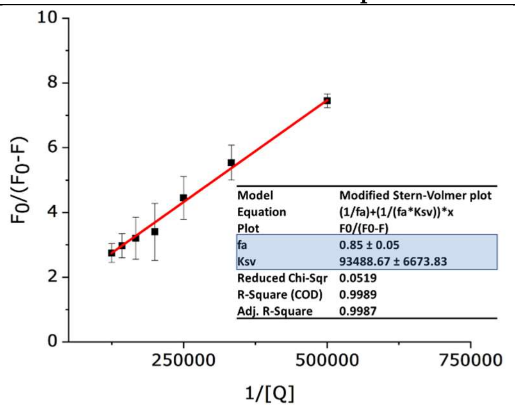

Eu oxo

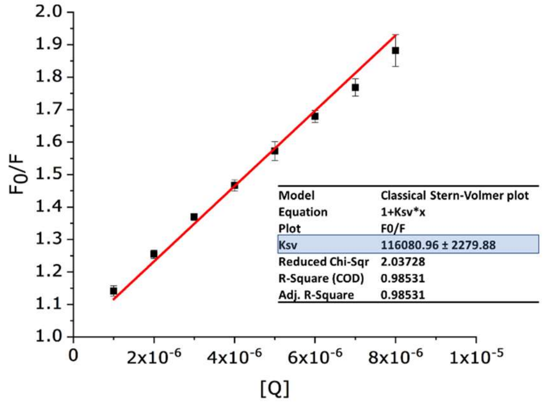

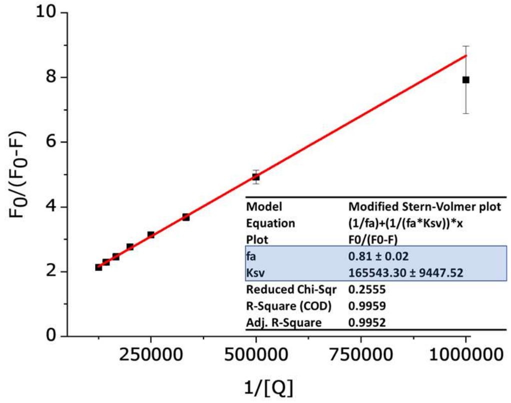

Gd oxo

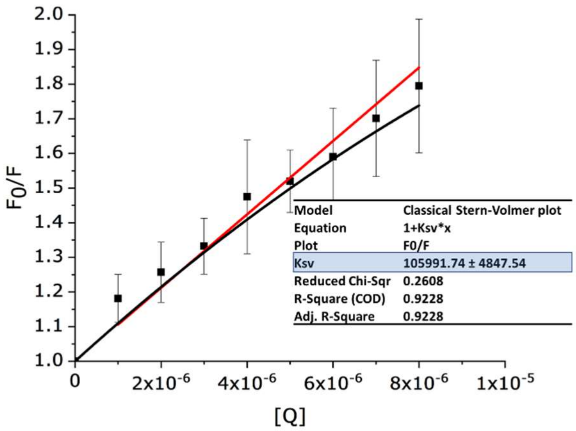

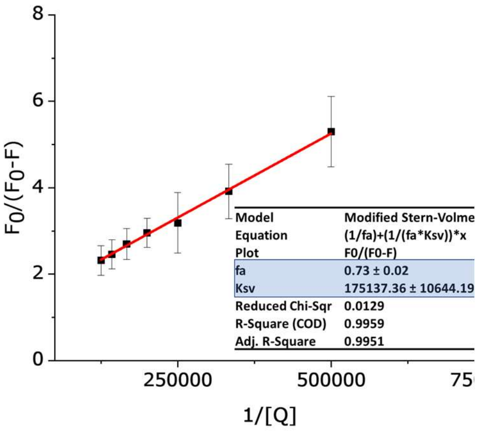

Tb oxo

Classical Stern-Volmer plot	Modified Stern-Volmer plot
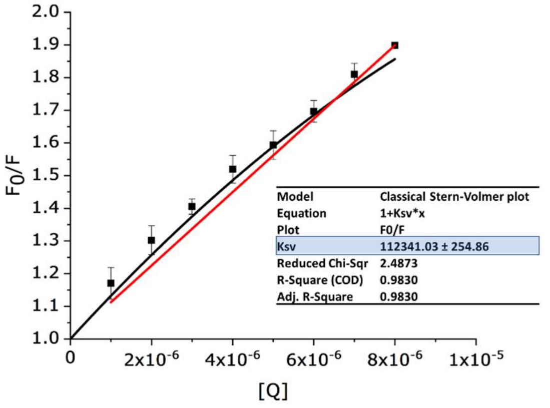	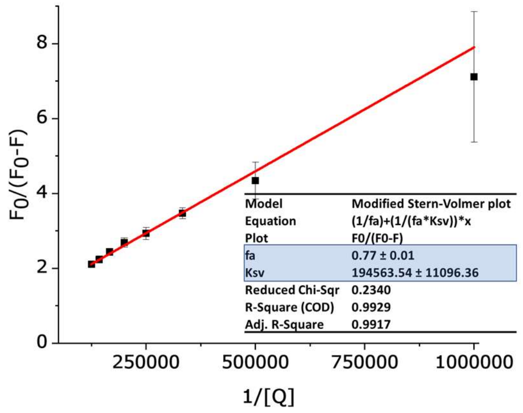

Figure S29. Classical (left) and modified (right) Stern-Volmer plots for the apo-Tf interaction assay.

Oxolinic acid

Ka

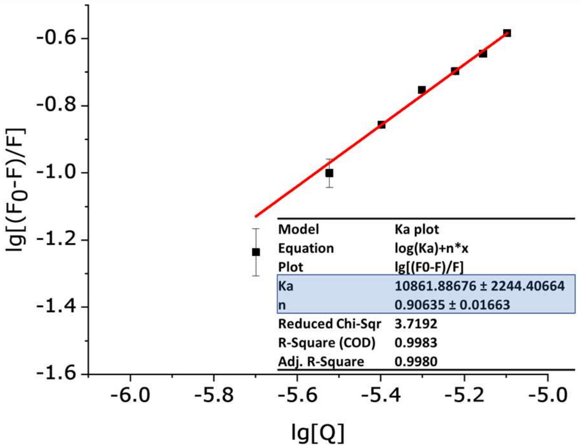

Kd

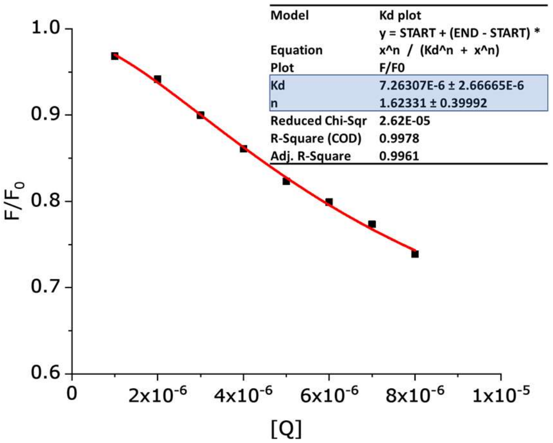

Y oxo

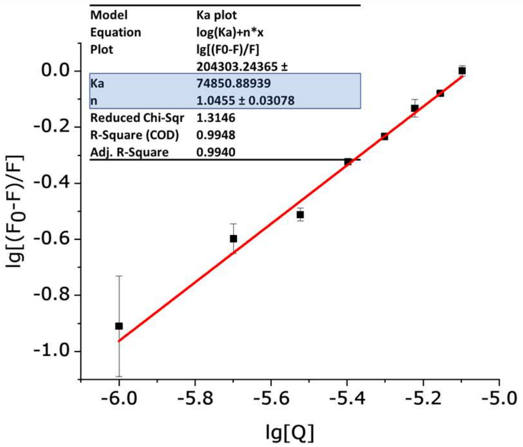

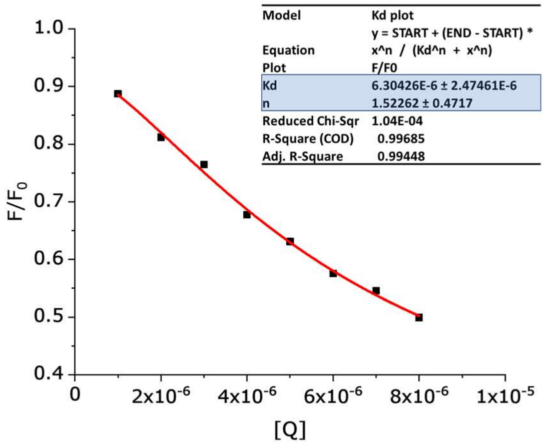

La oxo

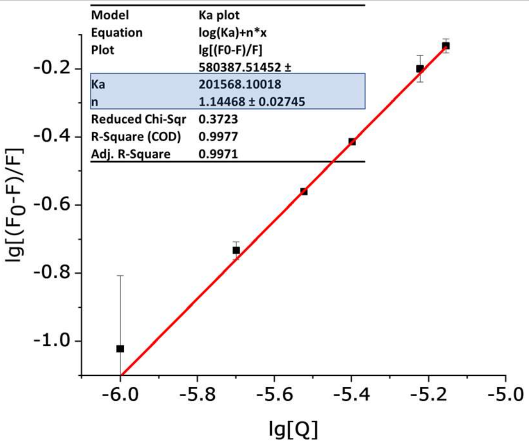

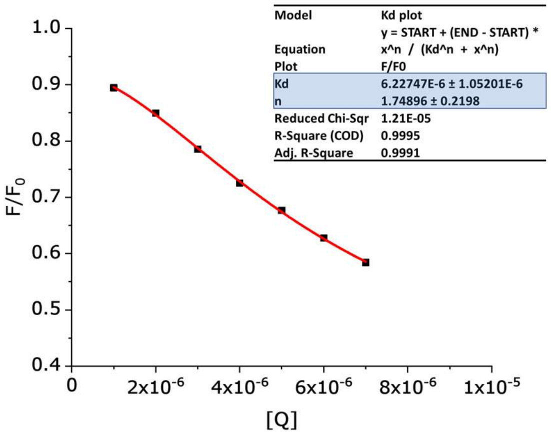

Sm oxo

Ka	Kd

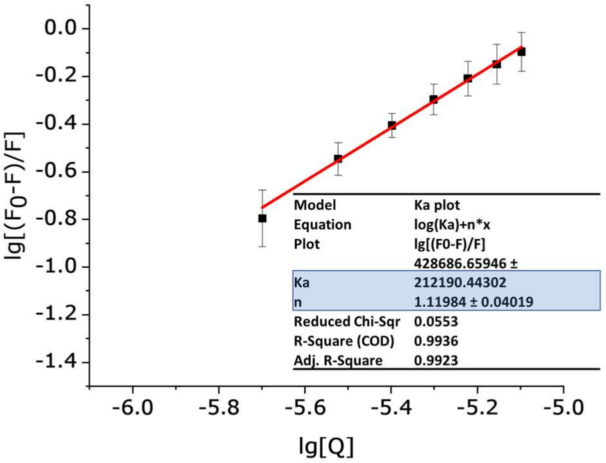

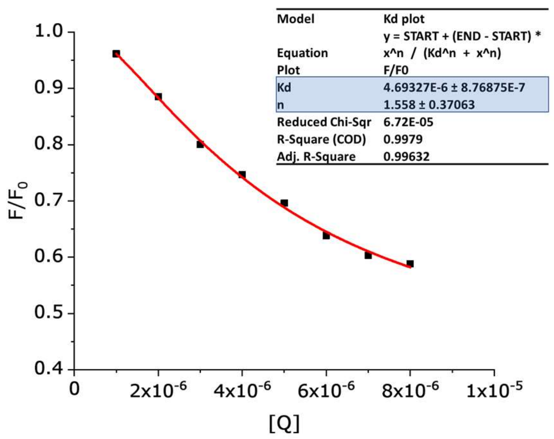

Eu oxo

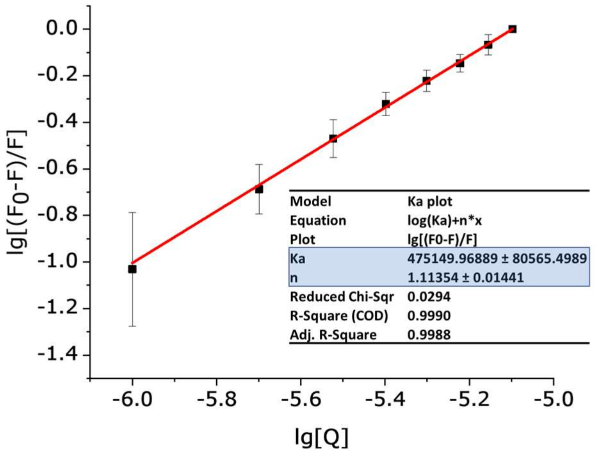

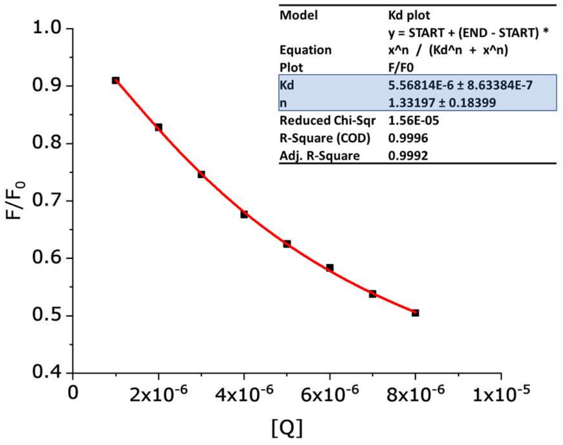

Gd oxo

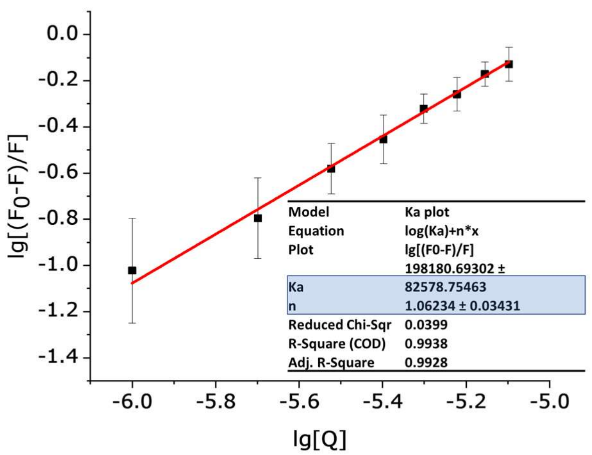

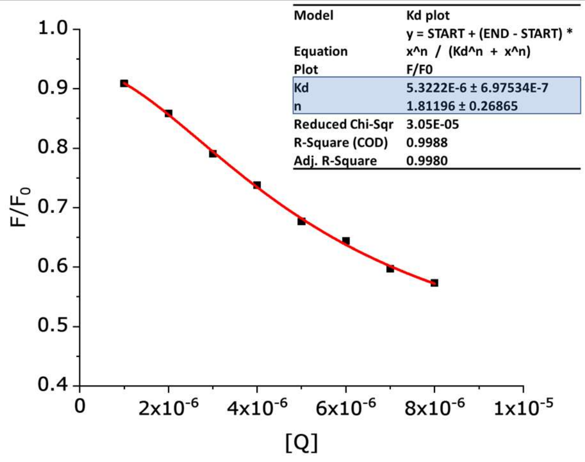

Tb oxo

Ka	Kd
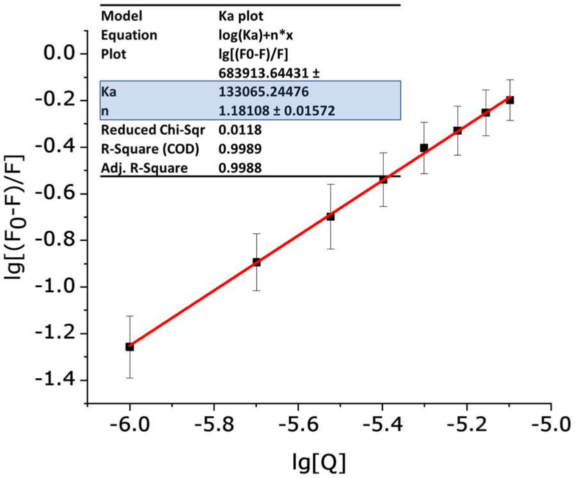	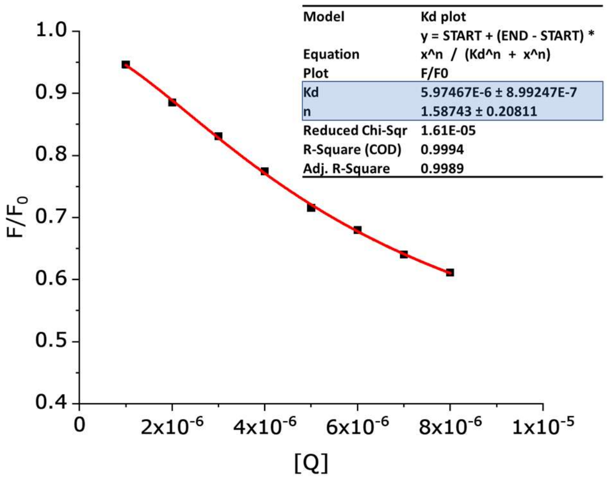

Figure S30. Ka and Kd for each of the studied compound-HSA systems.

Oxolinic acid	
**Ka**	**Kd**

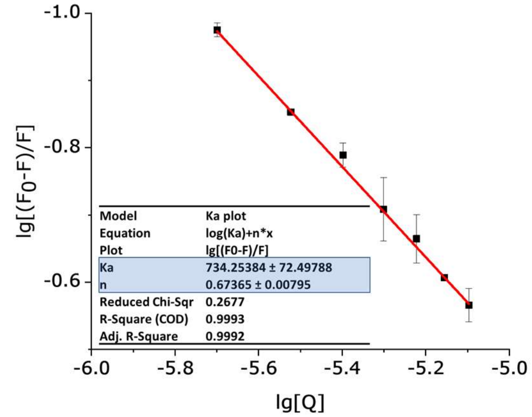

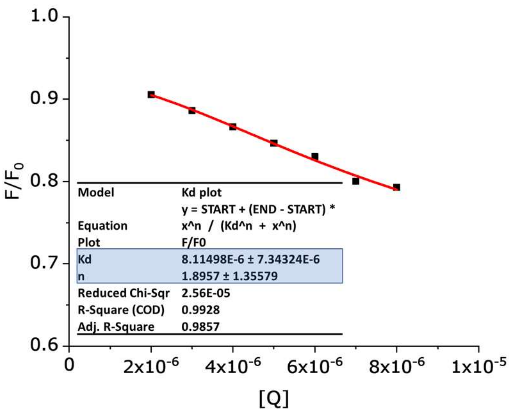

Y oxo

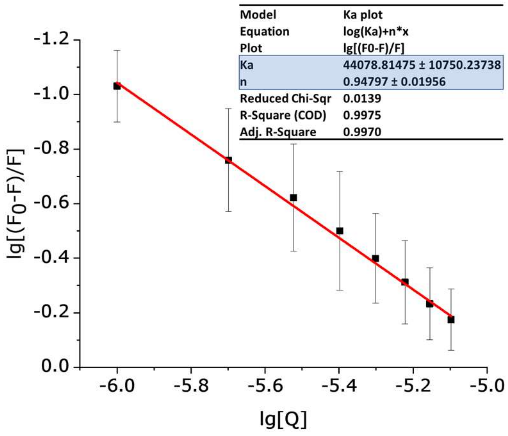

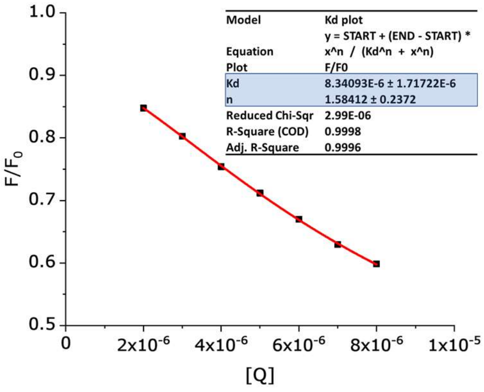

La oxo

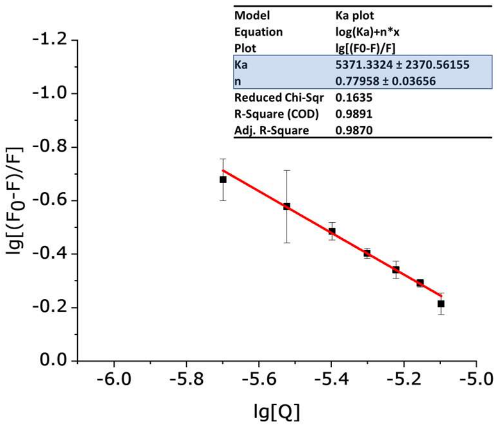

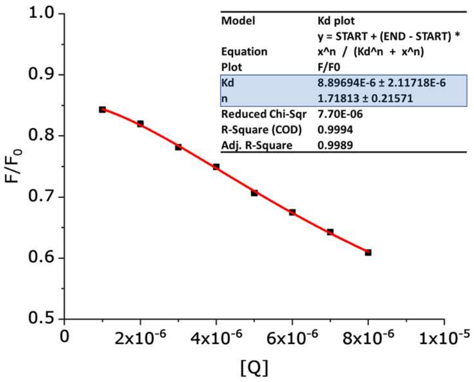

Sm oxo

Ka	Kd

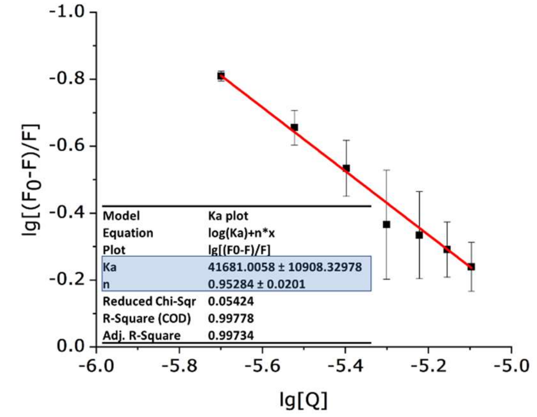

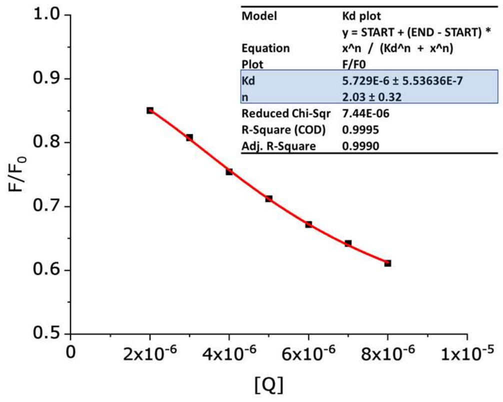

Eu oxo

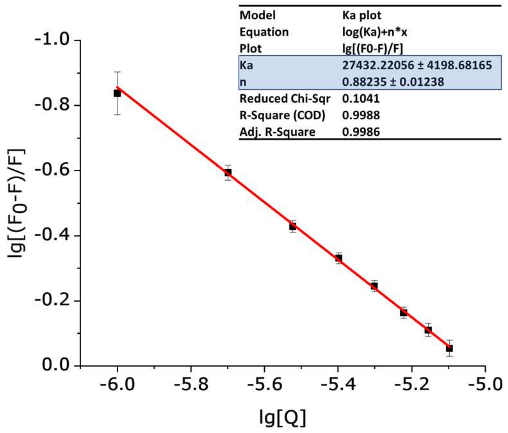

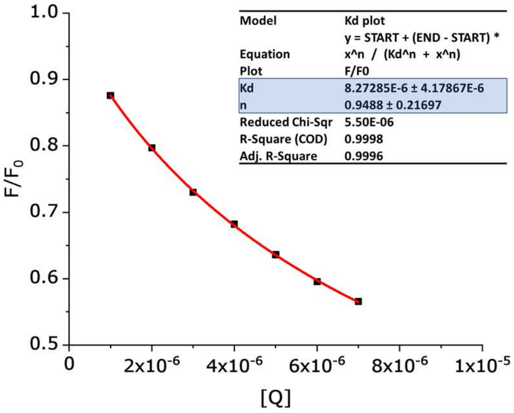

Gd oxo

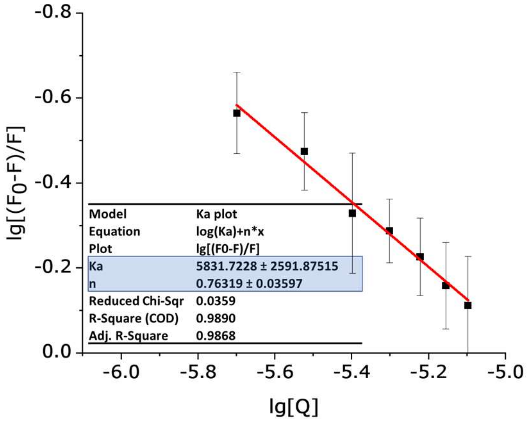

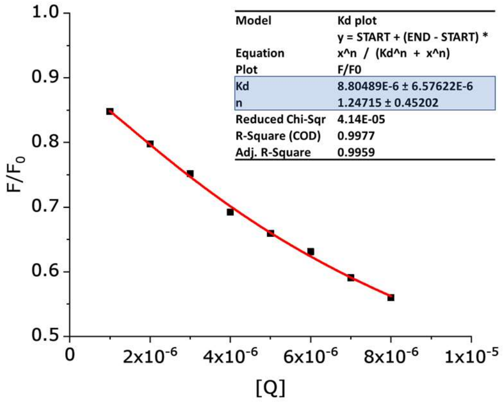

Tb oxo	
Ka	Kd
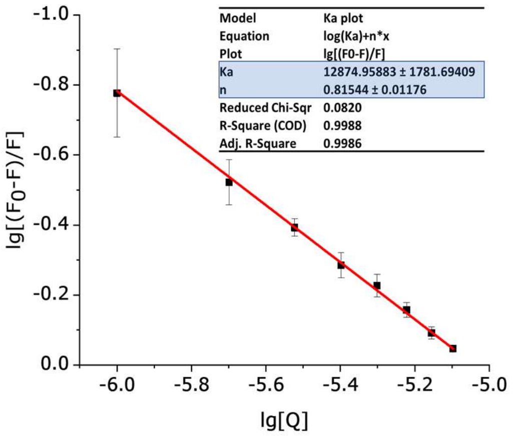	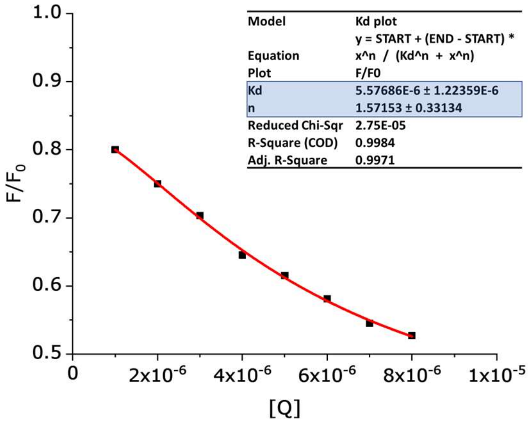

Figure S31. Representation of Ka and Kd constants for each of the apo-Tf interaction systems.

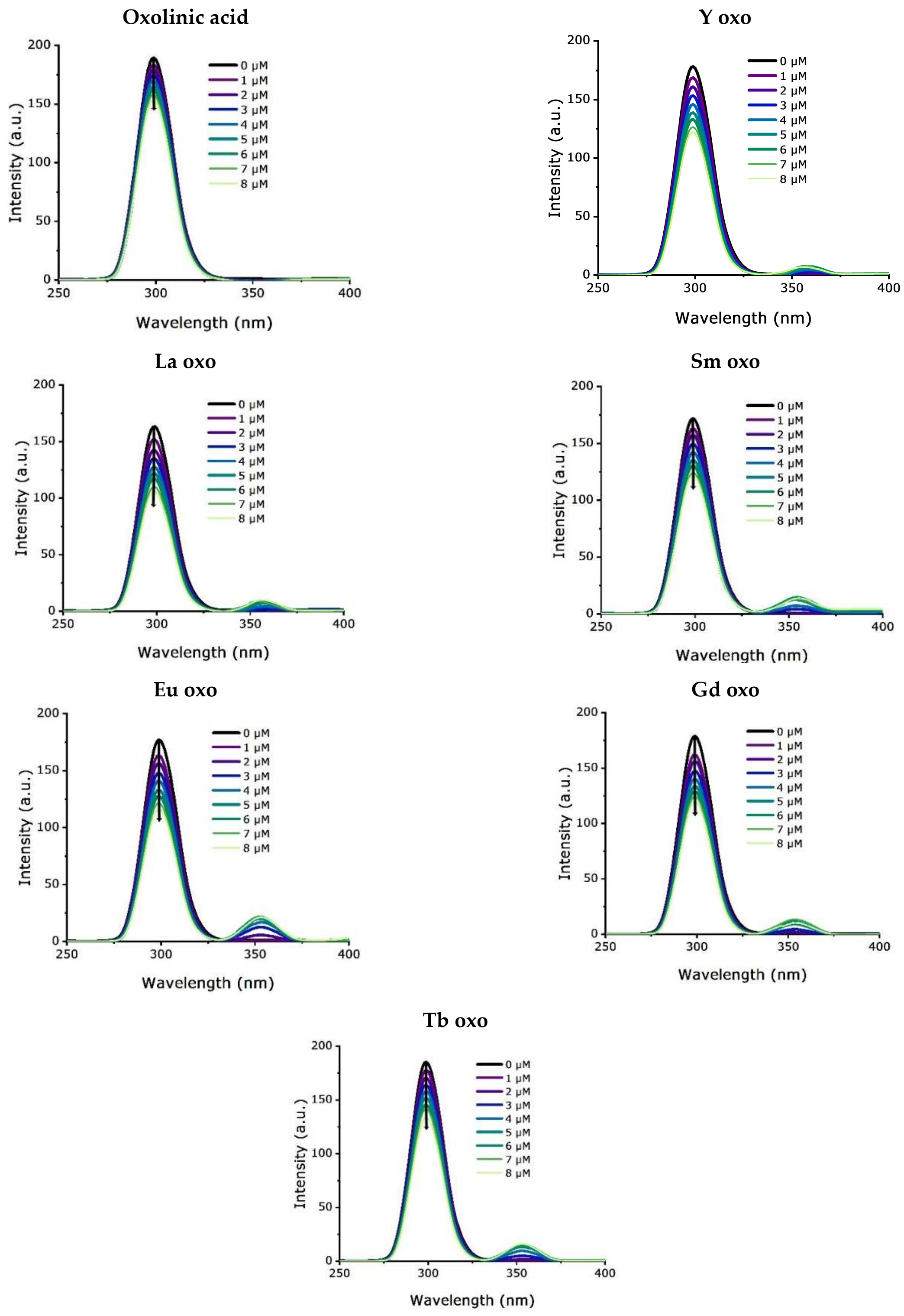

Figure S32. Synchronous spectra for the HSA interaction systems recorded at Δλ= 15 nm. [**HSA**] = 2.5 μM, [**Sm oxo**] = 0, 1, 2, 3, 4, 5, 6, 7, 8 μM.

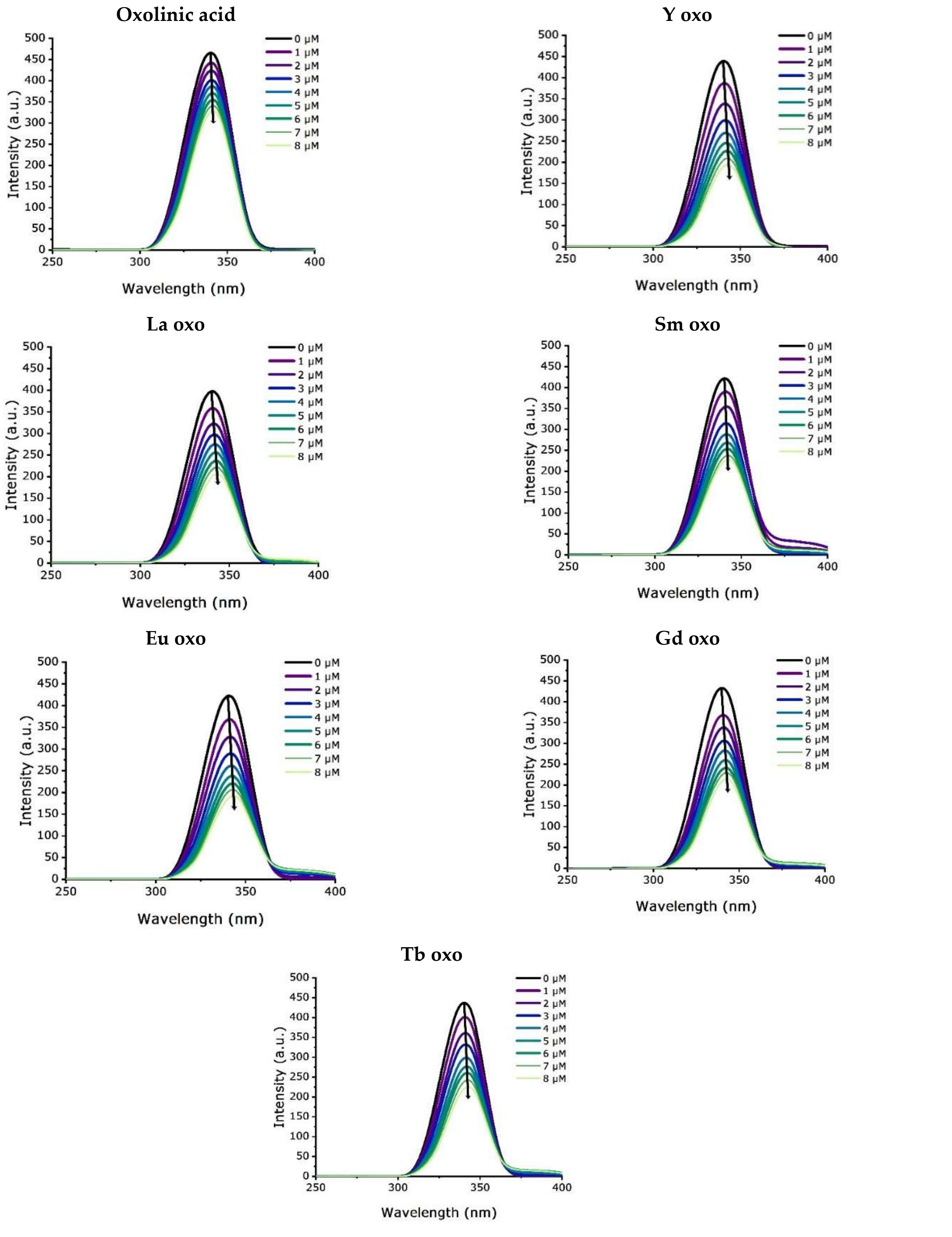

Figure S33. Synchronous spectra for the HSA interaction systems recorded at at $\Delta\lambda$= 60 nm. [**HSA**] = 2.5 μM, [**Sm oxo**] = 0, 1, 2, 3, 4, 5, 6, 7, 8 μM.

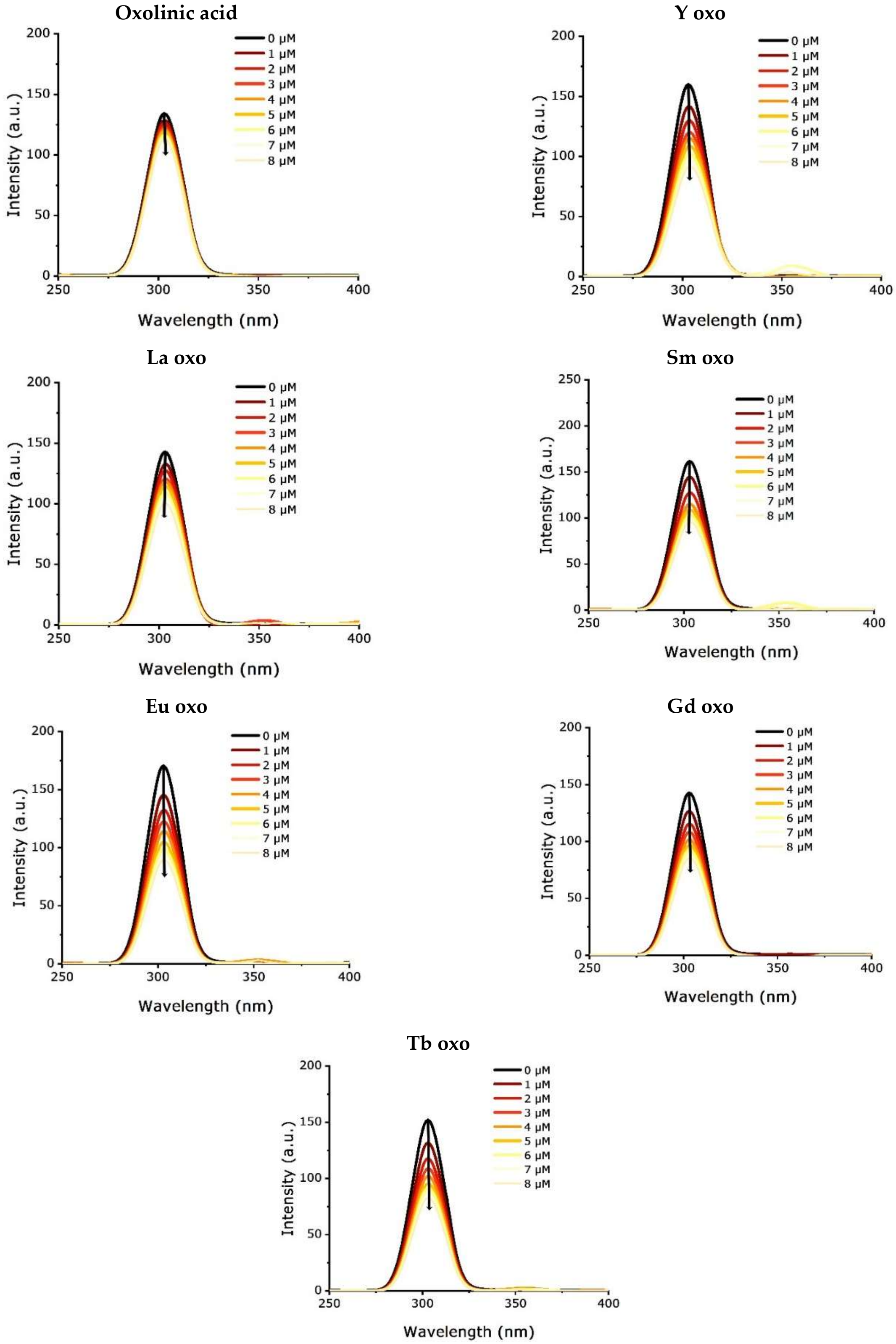

Figure S34. Synchronous spectra of the tested compounds - apo-Tf systems recorded at $\Delta\lambda$=15 nm. [**apo-Tf**] = 1 μM, [**compound**] = 0, 1, 2, 3, 4, 5, 6, 7, 8 μM.

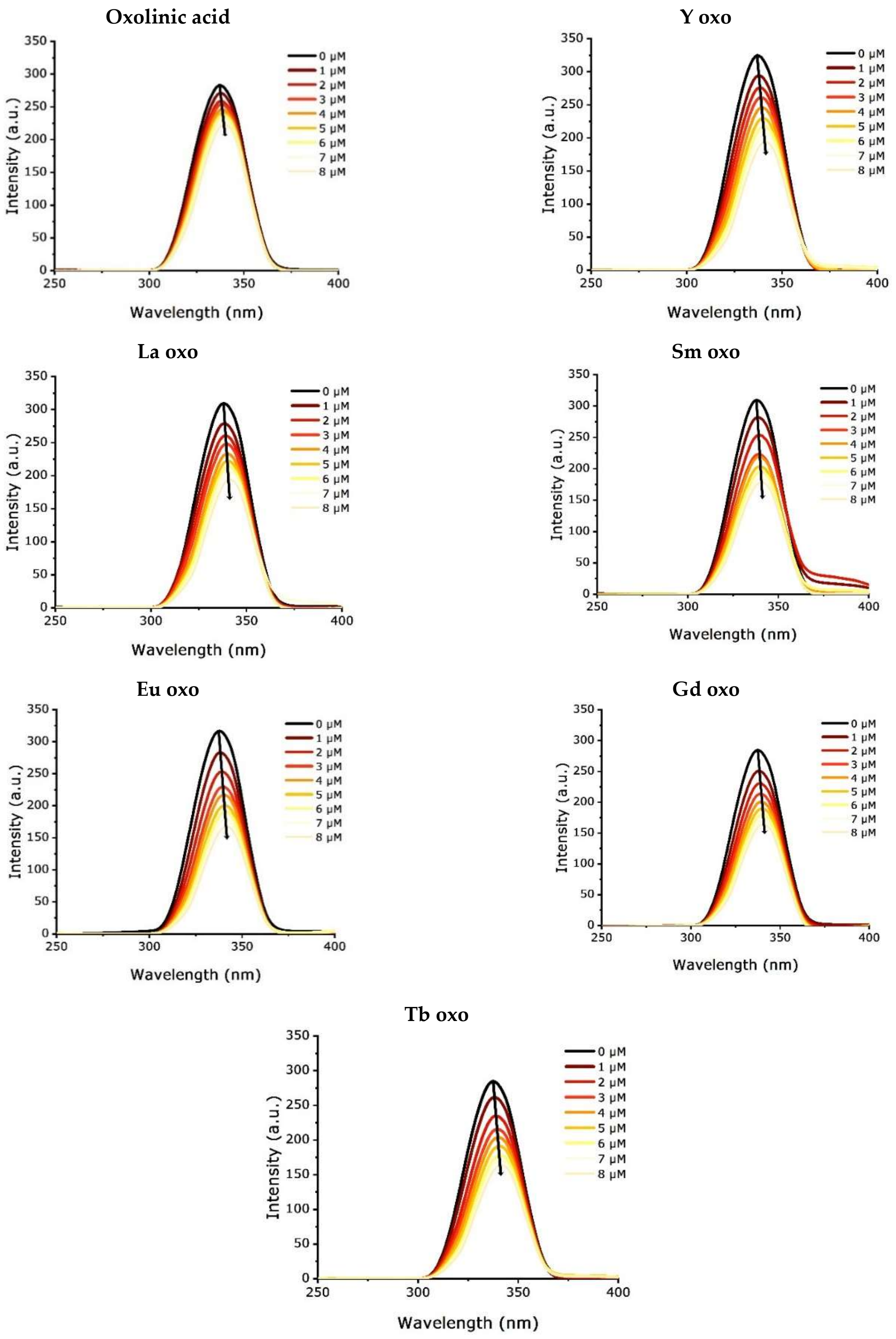

Figure S35. Synchronous spectra of the tested compounds - apo-Tf interaction systems recorded at $\Delta\lambda$=60 nm. [**apo-Tf**] = 1 μM, [**compound**] = 0, 1, 2, 3, 4, 5, 6, 7, 8 μM.
